# Unlocking the Potential of Photoelectrochemical Water Splitting via Heterointerface Charge Polarization

**DOI:** 10.1002/advs.202502384

**Published:** 2025-04-17

**Authors:** Li Xu, Xingming Ning, Jingjing Quan, Chenglong Li, Lan Yao, Qiang Weng, Pei Chen, Zhongwei An, Xinbing Chen

**Affiliations:** ^1^ Key Laboratory of Applied Surface and Colloid Chemistry (MOE) Shaanxi Key Laboratory for Advanced Energy Devices Shaanxi Engineering Laboratory for Advanced Energy Technology International Joint Research Center of Shaanxi Province for Photoelectric Materials Science School of Materials Science and Engineering Shaanxi Normal University Xi'an 710119 P. R. China

**Keywords:** charge polarization, charge transfer dynamics, heterointerface, in situ characterization, photoelectrochemical water splitting

## Abstract

The coupling of semiconductor (SC) and transition metal oxyhydroxide (TMOOH) is a promising approach for solar fuel production. However, the inevitable interfacial charge recombination and sluggish oxygen evolution reactions severely hinder the application of photoelectrochemical (PEC) device. This study demonstrates an innovative charge polarization strategy that simultaneously enhances both long‐range charge transfer and surface catalytic reaction dynamics through the rational construction of CoO_x_/MnO_x_ heterointerface in SC/TMOOH system. Kelvin probe force microscopy, in situ ultraviolet/visible spectroelectrochemistry, and density functional theory calculations indicate that the tunable charge polarization of Co^δ−^ and Mn^δ+^ can affect influences the SC/TMOOH and TMOOH/electrolyte interfaces, primarily through inducing the accelerated charge transfer dynamics (*K*_h_) and diminishing the adsorption of oxygen‐containing intermediates. As anticipated, the BiVO_4_/CoO_x_/MnO_x_/FeNiOOH exhibits an impressive photocurrent of 6.75 mA cm^−2^ at 1.23 V_RHE_, along with a superior photostability. Furthermore, the smart approach can also be harnessed in the BiVO_4_/CoO_x_/CeO_x_/FeNiOOH photoanode. This study provides a novel polarization strategy for the design of optimal photoanodes for PEC water splitting.

## Graphical abstract

This research presents a charge polarization approach aimed at enhancing both long‐range charge transfer and surface catalytic activities by incorporating a CoO_x_/MnO_x_ heterointerface within the semiconductor/transition metal oxyhydroxide system. As anticipated, the BiVO_4_/CoO_x_/MnO_x_/FeNiOOH exhibits an impressive photocurrent of 6.75 mA cm^−2^ at 1.23 V_RHE_, along with a superior photostability.

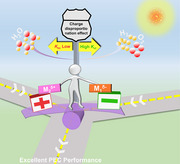

## 1 Introduction

Photoelectrochemical (PEC) water splitting emerges as a promising alternative strategy to achieve the “double carbon” goal.^[^ ^1^ ^]^ This process mainly includes light harvesting, photogenerated charge separation, and surface catalysis.^[^ ^2^ ^]^ Thereinto, the poor charge transport characteristics, along with significant charge recombination and slow kinetics of water oxidation, greatly hinder PEC water splitting efficiency.^[^ ^3^ ^]^

Recently, effective integration of semiconductor (SC) and electrocatalyst is essential for the development of desired PEC devices, which can effectively extract the photogenerated holes and offer the catalytic sites for oxygen evolution at a reduced overpotential.^[^ ^4^ ^]^ Among various electrocatalysts, transition metal oxyhydroxides (TMOOH) have recently gained significant attention due to their excellent stability, earth abundance, and superior surface catalytic efficiency.^[^ ^2^, ^5^ ^]^ For example, Zhang et al.^[^ ^6^ ^]^ reported a straight forward pH‐modulated technique for in situ growth of Fe_x_Ni_1‐x_OOH on BiVO_4_ photoanodes, leading to one of the highest currently known photocurrent density of 5.8 mA cm^−2^ at 1.23 V versus reversible hydrogen electrode (RHE, AM 1.5 G).

Although the considerable efforts made, the recorded photocurrent density of BiVO_4_/electrocatalyst coupling systems is still significantly lower than the theoretical value of 7.5 mA cm^−2^ when exposed to standard one sun illumination and at an external potential of 1.23 V versus RHE. Moreover, the photocurrent densities reported for BiVO_4_‐based photoanodes typically fall within the range of 3.5 to 6.0 mA cm^−2^ (see Table S1, Supporting Information, for high‐efficiency BiVO_4_‐based photoanodes). Only a limited number of studies have achieved photocurrent densities exceeding 6.0 mA cm^−2^. The significant gap primarily arises from the inevitable interface charge recombination, namely, current methods for loading electrocatalysts, such as solvothermal, spin coating, and water baths, are limited and inevitably create some defects during this process, which will significantly lead to the recombination of photogenerated carriers.^[^ ^7^ ^]^

To tackle the challenge of charge recombination at the SC/TMOOH interface, various interfacial regulation layers (IRLs), including black phosphorene,^[^ ^8^ ^]^ porphyrin molecules,^[^ ^9^ ^]^ and transition metal‐based regulation layers^[^ ^10^ ^]^ have been incorporated at this interface to mitigate the inevitable charge recombination. Although the PEC performance in current system showed further improvement over the previously discussed SC/TMOOH coupling system, it still falls short of the anticipated levels. The detailed explanations for this are as follows: 1) the hole transfer dynamics in the IRLs are notably slower than the ultrafast light absorption process (within the timescale of picoseconds to nanoseconds), thereby degrading the long‐term operation of integrated photoanodes; 2) these IRLs mainly possess a singular functional characteristic and are unable to simultaneously expedite charge transfer and boost surface catalysis; 3) the reported IRLs tend to demonstrate short‐range hole transfer behavior due to the lack of sufficient driving force, which consequently leads to a significant depletion of photogenerated charge.

Drawing inspiration from these studies, it is highly alluring but very challenging to construct a heterointerface with charge polarization effect to unlock above bottleneck. The heterointerface, functioning akin to a seesaw, disrupts the original equilibrium via internal charge transfer. This disruption leads to the formation of hole transfer sites with low oxidation states and surface catalytic centers with high oxidation states. Where these newly‐formed sites can concurrently promote the dynamics of long‐range charge transfer and surface catalysis. As a result, the recombination caused by the mismatch in the time scale (ranging from 10^−12^ to 10^0^) is significantly reduced. Of course, in order to further achieve the goal and reveal this mechanism, BiVO_4_ (donated as BV), which features a narrow bandgap, a deep valence band (VB) position, and satisfactory stability, is employed as a photoanode.^[^ ^11^ ^]^ Correspondingly, BV/FeNiOOH can serve as a state‐of‐the‐art prototypical model.

Herein, we present the application of heterointerface charge polarization as an active IRL to enhance the performance of PEC water splitting. Both experiments results and theoretical analyses suggest that the charge polarization effect can simultaneously produce two beneficial sites, especially for charge separation and surface catalysis, respectively. The former greatly reduces the inevitable charge recombination, and the latter weakens the adsorption of oxygen‐containing intermediates. As a result, the integrated system BV/CoO_x_/MnO_x_/FeNiOOH exhibited a photocurrent density of 6.75 mA cm^−2^ and a stability of 30 h under AM 1.5 G illumination. Moreover, this effect also demonstrated good universality in the BV/CoO_x_/CeO_x_/FeNiOOH system.

## 2 Results and Discussion

### 2.1 Morphology and Structure Characterizations

**Figure 1:**
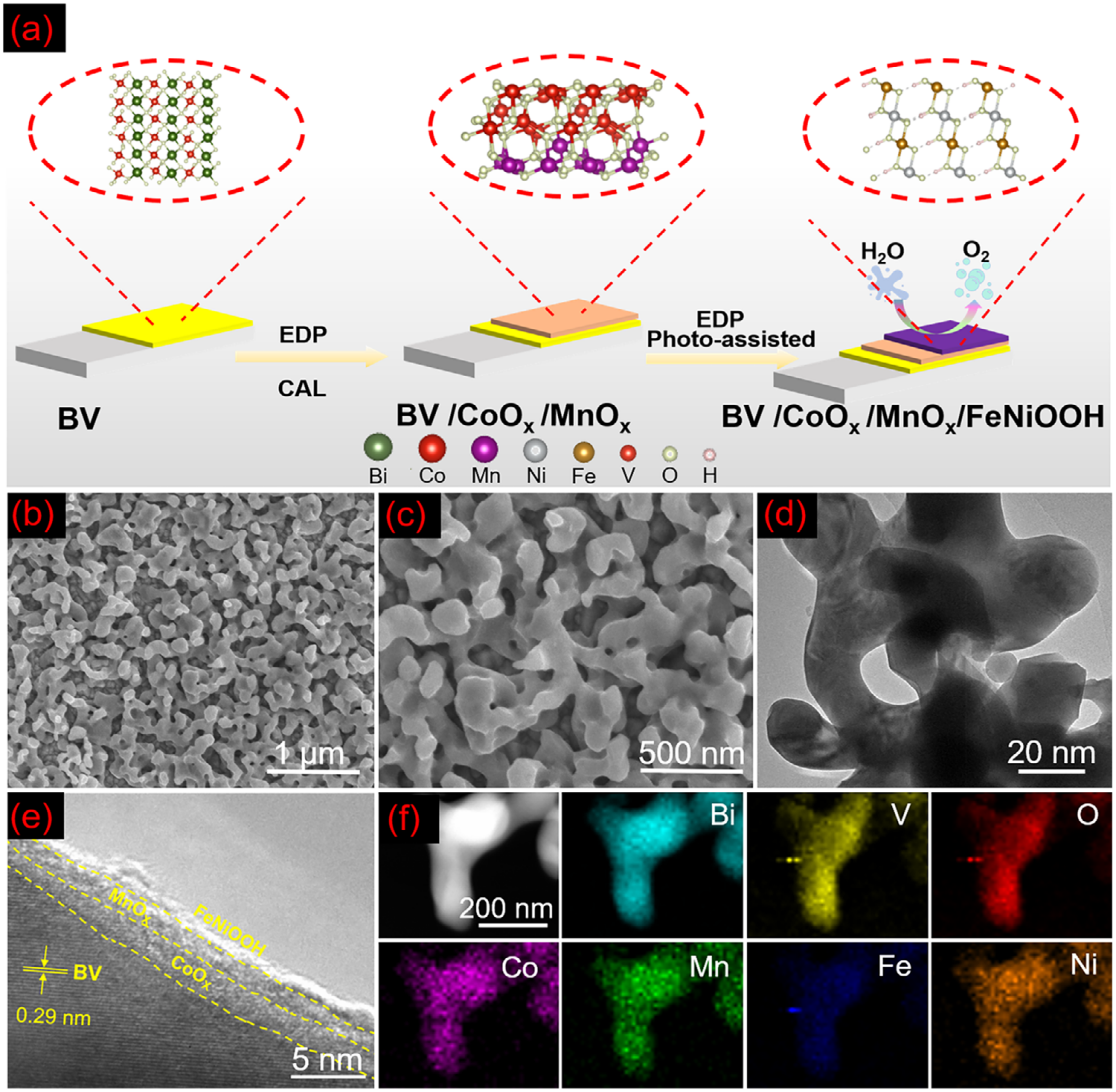
a) Schematic synthesis route of the BV/CoO_x_/MnO_x_/FeNiOOH photoanode. b,c) SEM. d) TEM and e) HR‐TEM. f) EDS mappings of Bi, V, O, Co, Mn, Fe, and Ni.

The BV/CoO_x_/MnO_x_/FeNiOOH array as a desired integrated configuration for PEC water splitting was constructed by typical fabrication process, as shown in **Figure** **1**a. Briefly, a porous BV film was grown on a fluorine‐doped tin oxide (FTO) glass substrate. For surface morphology, advanced characterization techniques like scanning electron microscopy (SEM) and transmission electron microscopy (TEM) were used to explore its morphology of photoanode. As shown in Figure S1 (Supporting Information), BV was uniformly dispersed on the FTO substrate and displayed its unique worm‐like porous structure with an average diameter of 100–300 nm and having a thickness of ≈400 nm. The obvious lattice fringe of 0.29 nm corresponds to the (211) plane of BV^[^ ^7^, ^12^ ^]^ (Figure S2, Supporting Information). The crystal structure of BV was analyzed using X‐ray diffraction (XRD), as illustrated in Figure S3a (Supporting Information), and the BV film exhibits a monoclinic structure (PDF # 14–0688), which aligns with earlier findings.^[^ ^13^ ^]^ Additionally, the Raman characteristic peaks observed at 210.0, 324.0, 366.0, 640.0, 710.0, and 826.0 cm^−1^ provide further evidence for the successful synthesis of BV arrays (Figure S3b, Supporting Information).

Subsequently, the heterointerface moiety (CoO_x_/MnO_x_) is coated on the BV surface via electrodeposition (EDP) and calcination (CAL) processes. Compared with pristine BV, BV/CoO_x_/MnO_x_ presents a relatively rough surface, possibly owing to the growth of the CoO_x_/MnO_x_ moiety. Similar results could also be supported on the SEM images of BV/CoO_x_/MnO_x_ (Figure S4a,b, Supporting Information). Elemental mappings obtained through energy‐dispersive spectroscopy (EDS, Figure S4c, Supporting Information) distinctly indicated that the Bi and V elements were predominantly situated in the central region of the BV/CoO_x_/MnO_x_. In contrast, the distribution of Co and Mn elements was uniform throughout the entire composite, further supporting the shell‐core‐like structure of BV/CoO_x_/MnO_x_. More importantly, the formation of perfect heterointerface could be confirmed by the high‐resolution TEM image (HR‐TEM, Figure S4d, Supporting Information). Typically, the CoO_x_/MnO_x_ nanosheet is ≈4 nm in thickness, and CoO_x_ is only ≈2.5 nm (Figure S5, Supporting Information). Additionally, the findings related to the complementary EDS elemental mappings further demonstrate the construction of the aforementioned heterostructure (Figures S6 and S7, Supporting Information).

X‐ray photoelectron spectroscopy (XPS) measurements were carried out to further investigate the composition and chemical state of different samples. In Figures S8 and S9 (Supporting Information), the XPS survey spectra distinctly identified the presence of Bi, V, O, Co, and Mn elements, preliminary confirming the synthesis of BV/CoO_x_/MnO_x_ photoanodes. The high‐resolution XPS spectra of BV show Bi 4f peaks at 158.17 eV (Bi 4f_7/2_) and 163.41 eV (Bi 4f_5/2_), which indicates the presence of Bi^3+^. Furthermore, the V 2p peaks at 516.11 eV (V 2p_3/2_) and 523.87 eV (V 2p_1/2_) are associated with V^5+^ in the BV sample (Figure S8, Supporting Information).^[^ ^14^ ^]^ Furthermore, the XPS spectrum for Co 2p reveals two primary peaks at Co 2p_3/2_ (780.50 and 782.27 eV) and Co 2p_1/2_ (796.22 and 797.69 eV), along with satellite features at 786.80 and 803.85 eV, indicating that cobalt is in the Co^3+^ and Co^2+^ oxidation state.^[^ ^15^ ^]^ Similarly, the peak fitting for Mn 2p has a mixed‐valence state, which includes Mn^2+^ (641.25 and 653.19 eV), Mn^3+^ (642.48 and 654.51 eV), and Mn^4+^ (644.23 and 655.77 eV), corresponding to Mn 2p_3/2_ and Mn 2p_1/2_, respectively (Figure S9, Supporting Information).^[^ ^16^ ^]^

In addition, there are no discernible signals in the XRD patterns or Raman spectra (Figure S10, Supporting Information), mainly due to the minimal loading amount, extremely thin thickness, and uniform distribution, which aligns with the previously mentioned EDS elemental mappings (Figure S4c, Supporting Information). Significantly, the positions of the corresponding peaks in the Raman spectra remain unchanged, suggesting that CoO_x_/MnO_x_ did not alter the intrinsic structure of BV (as supported by the XPS results discussed in Supporting information). Similar results can be confirmed in the BV/CoO_x_ and BV/MnO_x_ systems (Figures S11–S15, Supporting Information).

Afterward, to alleviate the sluggish process of water oxidation, FeNiOOH (Figures S16 and S17, Supporting Information), as an excellent OER catalysts, was coated on the surfaces of BV/CoO_x_/MnO_x_ through photo‐assisted EDP method. Combining SEM and TEM images of FeNiOOH layer as well as the results of EDS mapping, XRD, and Raman spectra (Figure 1b–f; Figure S18, Supporting Information), we can confirm that the loading of FeNiOOH on the surface of various BV‐based photoanode, thereinto, BV/CoO_x_/MnO_x_/FeNiOOH photoelectrodes are also included.

### 2.2 Photoelectrochemical Properties

**Figure 2:**
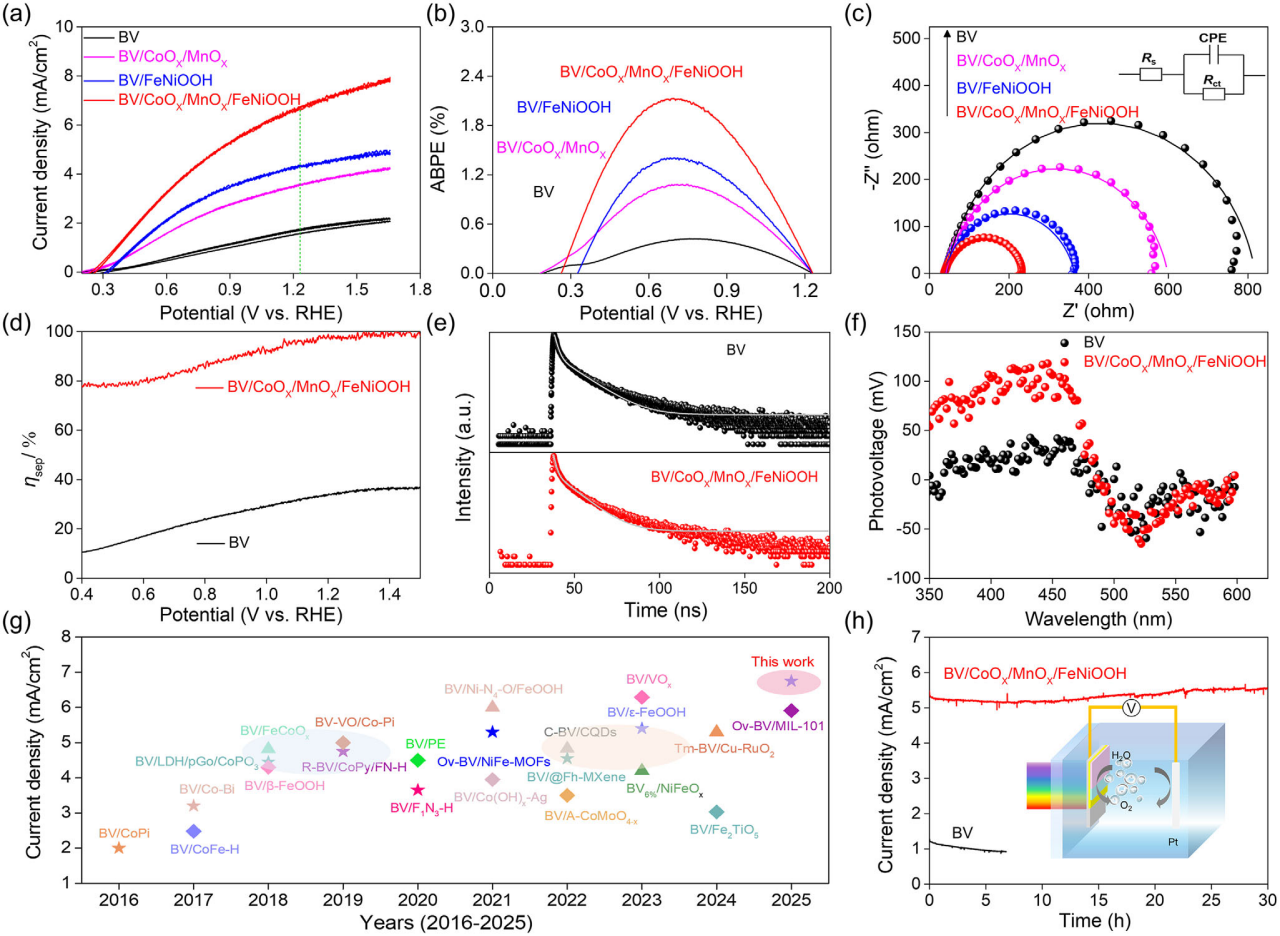
a) Linear‐sweep voltametric (LSV) curves. b) ABPE results. c) EIS Nyquist plots under illumination. d) Surface charge separation efficiency (*η*_sep_). e) TRPL curves. f) SPV spectra. g) Photocurrent densities based on BV‐based photoanodes at the 1.23 V_RHE_ in recent years (2016–2025). h) *I‐t* stability tests.

The PEC water splitting performance of the BV‐based photoanode was evaluated in a three‐electrode system under AM 1.5 G illumination conditions (100 mW cm^−2^, solar simulator). Herein, a solar simulator equipped with a total‐reflection mirror and an AM 1.5 G filter was employed to assess the performance of the PEC system. The spectrum of the solar simulator was analyzed using a spectrometer, and the results revealed a close match with the AM 1.5 G standard spectrum (Figure S19, Supporting Information). As illustrated in **Figure** **2**a, the pure BV photoanode exhibited a weak photocurrent density of 1.74 mA cm^−2^ (1.23 V versus RHE) due to poor surface catalytic activity and severe surface charge recombination (discussion in introduction) of BV. To address this issue, loading of OER catalysts onto BV is effective and necessary to boost the transfer of photogenerated holes to electrolyte. As expected, by integrating FeNiOOH, the BV/FeNiOOH photoanode enhances the photocurrent density from 1.74 to 4.29 mA cm^−2^ at 1.23 V versus RHE. Nevertheless, the photocurrent density of BV/FeNiOOH obtained is still far less its theoretical value (7.5 mA cm^−2^), which is mainly contributed to the severe charge recombination at the BV/FeNiOOH interface (It is important to highlight that certain spikes are evident in a chopped‐light test, Figure S28a (Supporting Information), indicating significant charge recombination, as demonstrated in our prior report^[^ ^7b^ ^]^). Interestingly, when CoO_x_/MnO_x_ layers ≈4 nm (Figure S4d, Supporting Information) thick were incorporated at the BV/FeNiOOH interface, the optimized BV/CoO_x_/MnO_x_/FeNiOOH photoanode demonstrates an impressive photocurrent density of 6.75 mA cm^−2^ at 1.23 V versus RHE (Figure 2a; Figure S20, Supporting Information), which is close to its theoretical photocurrent density of 7.5 mA cm^−2^. As well, the obtained photocurrent density of the BV/CoO_x_/MnO_x_/FeNiOOH photoanode is the highest among all these BV‐based photoanodes (Figure 2a; Figures S21a and S22a, Supporting Information), implying that the introduction of CoO_x_/MnO_x_ heterointerface can significantly promote PEC water splitting performance.

Based on the *J–V* curve, the applied bias photon‐to‐current efficiency (ABPE) values of the BV‐based photoanodes were assessed. As presented in Figure 2b and Figure S22b (Supporting Information), the ABPE value of BV/CoO_x_/MnO_x_/FeNiOOH reaches 2.12% at 0.67 V versus RHE, surpassing the individual BV (0.42%), BV/CoO_x_ (0.84%), BV/MnO_x_ (0.68%), BV/CoO_x_/MnO_x_ (1.08%) and BV/FeNiOOH (1.40%). The improved energy conversion efficiency of relevant photoanodes is supported by the incident photon to current conversion efficiency (IPCE) value at a specific wavelength range (Figure S23a, Supporting Information).

The interface characteristics of BV‐based photoanodes were investigated using electrochemical impedance spectroscopy (EIS) under illumination. The Nyquist results were fitted with an equivalent circuit as presented in Figure 2c and Figures S21b and S22c (Supporting Information), and corresponding values are listed in Table S2 (Supporting Information). The BV/CoO_x_/MnO_x_/FeNiOOH exhibits a lower charge‐transfer resistance (*R*_ct_, 194 Ω) than that of BV (775 Ω), BV/CoO_x_ (618 Ω), BV/MnO_x_ (670 Ω), BV/CoO_x_/MnO_x_ (557 Ω), and BV/FeNiOOH (327 Ω), indicating that the introduction of CoO_x_/MnO_x_ heterointerfaces enhances the charge transfer dynamics, which is consistent with its elevated photocurrent density and measured surface charge separation efficiency (*η*_sep_, Figure 2d; Figure S23b, Supporting Information). Moreover, the time‐resolved photoluminescence (TRPL) spectroscopy was employed to explore the decay kinetic of photogenerated carrier lifetimes. As illustrated in Figure 2e and Table S3 (Supporting Information), the average lifetimes of the photogenerated carriers for the BV/CoO_x_/MnO_x_/FeNiOOH (4.45 ns) is longer than pure BV (2.43 ns), suggesting the lowest charge carrier recombination rate for target photoanode, which is consistent with the findings of reduced fluorescence quenching (Figure S23c, Supporting Information) and is advantageous for these holes to engage in surface water oxidation reactions.

To further uncover the surface photovoltaic properties, surface photovoltage (SPV) was carried out. Form Figure 2f, both the BV and BV/CoO_x_/MnO_x_/FeNiOOH exhibit positive surface photovoltage under illumination, meaning that the enrichment of holes at the surface.^[^ ^11^, ^17^ ^]^ The surface photovoltage of BV/CoO_x_/MnO_x_/FeNiOOH is significantly greater than that of BV, elucidating that substantial accumulation of photogenerated holes on the surface FeNiOOH, which is further corroborated by the longer carrier lifetimes (Figure 2e). Given the above mentioned evidence of elevated photocurrent, we assess the PEC performance of the BV/CoO_x_/MnO_x_/FeNiOOH in comparison to previously reported BV‐based photoanodes (Figure 2g), and observe that our designed photoanode shows considerable competitiveness (Table S1, Supporting Information).

Except for the high solar conversion efficiency, the photostability cannot be ignored for practical application. As exhibited in Figure 2h, photocurrent of the bare BV suffers from an obvious decrease from 1.29 to 0.92 mA cm^−2^ within 6.7 h during PEC water splitting process, which is mainly attributed to severe photocorrosion.^[^ ^18^ ^]^ Remarkably, after the decoration of CoO_x_/MnO_x_, the BV/CoO_x_/MnO_x_/FeNiOOH photoanode exhibits outstanding stability at 1.0 V_RHE_ for 30 h. After the stability test, SEM image, Raman spectra, and LSV results of the BV/CoO_x_/MnO_x_/FeNiOOH photoanode show no obvious difference from that before the stability test (Figures S24 and S25, Supporting Information), highlighting its exceptional stability. Furthermore, H_2_ and O_2_ production in the BV/CoO_x_/MnO_x_/FeNiOOH system was measured by an online gas chromatography. The yields of H_2_ and O_2_ are close to 2:1 ratio of the water splitting reaction, exhibiting a Faradaic efficiency of nearly 97%. suggesting that nearly all the photocurrents were effectively utilized for the purpose of water splitting (Figure S26, Supporting Information).

### 2.3 Charge Transfer Kinetics Analysis

In general, the PEC efficiency of photoelectrode is determined by the following three factors: 1) light harvesting efficiency (*ƞ*_abs_); 2) charge separation efficiency (*ƞ*_sep_); 3) surface catalysis efficiency (*ƞ*_sur_). To clarify an in‐depth understanding of the reasons of CoO_x_/MnO_x_ heterointerface for the improvement in PEC water splitting performance, UV–vis diffuse reflectance spectroscopy was used to explore its light‐capturing ability. Compared with BV, an overlapping absorbance curves can be observed in the Figure S27 (Supporting Information), proving that charge separation efficiency and surface catalysis efficiency are mostly responsible for the improvement in PEC performance.

**Figure 3:**
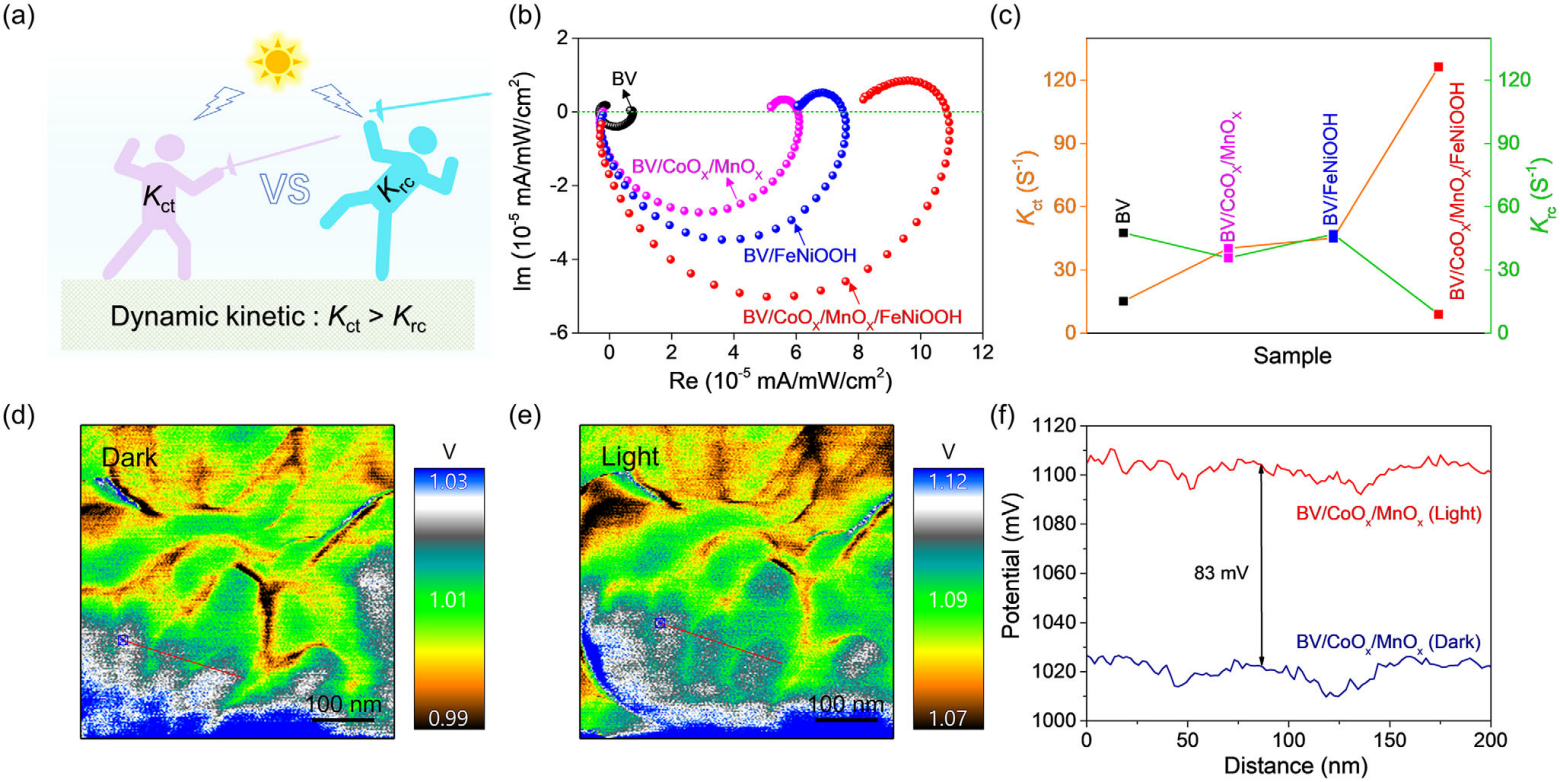
a) The principle of the IMPS manufacturing. b) IMPS complex plots of different samples. c) Charge transfer rate constant (*K*_ct_) and recombination rate constant (*K*_rc_). d,e) KPFM results images in dark (d) and under illumination (e) for the BV/CoO_x_/MnO_x_. f) Surface potential values in dark and under illumination for the BV/CoO_x_/MnO_x_.

Intensity modulated photocurrent spectroscopy (IMPS), developed by Peter and colleagues, was measured to investigate charge separation/recombination (**Figure** **3**a).^[^ ^19^ ^]^ Where *K*_ct_ refers to the charge transfer rate constant, while *K*_rc_ denotes the charge recombination rate constant. When *K*_ct_ exceeds *K*_rc_, the prevailing mechanism is the charge separation, conversely, if *K*_rc_ is greater, charge recombination predominates. As shown in Figure 3b,c, the *K*_rc_ of BV is significantly larger than the *K*_ct_ due to severe charge recombination and sluggish surface catalysis. After coupling with the FeNiOOH electrocatalyst, *K*_ct_ is remarkably enhanced, but it remains smaller than *K*_rc_ (Figure 3c) because of the inevitable interfacial charge recombination, as evidenced by the fast current decay observed in the *I‐t* curves (Figure S28a, Supporting Information). Interestingly, when CoO_x_/MnO_x_ heterointerface was inserted into the layer of FeNiOOH and BV, the *K*_ct_ is greater than *K*_rc_ implying that the rationally designed heterointerface can effectively suppress interface charge recombination, which is in good agreement with the slow decay trend in the *I–t* curve (Figure S28a,b, Supporting Information). In addition, the relevant transient time (*τ*_d_) of different samples can be calculated using the formula *τ*_d_ = 1/(2π*f*_IMPS_), where *f*_IMPS_ stands for the frequency at the lowest point.^[^ ^20^ ^]^ Typically, the *τ*_d_ values show the decreasing trend: BV > BV/CoO_x_/MnO_x_ > BV/FeNiOOH > BV/CoO_x_/MnO_x_/FeNiOOH (Figure S28c and Table S4, Supporting Information). Notably, the *τ*_d_ value of BV/CoO_x_/MnO_x_/FeNiOOH (0.34 ms) is much less than that of BV/FeNiOOH (0.50 ms), indicating that the heterointerface accelerates the hole transfer dynamics, consistent with the elevated *K*_ct_ value and reduced resistance (Figure S29, Supporting Information).

To better understand the mechanisms that contribute to the significant improvement in charge separation efficiency, Kelvin probe force microscopy (KPFM) was employed to investigate the interfacial charge transfer capability under the dark and light conditions. Figure 3d and Figure S30a (Supporting Information) exhibit the topographic images of the BV/CoO_x_/MnO_x_ photoanodes and reflect relatively low potential in the darkness. Under light conditions, the positive surface potential of the BV/CoO_x_/MnO_x_ photoanodes was evidently increased (Figure 3e; Figure S30b, Supporting Information). Specifically, the line scanning of the surface potential under both light exposure and darkness exceeds 83 mV (Figure 3f), which significantly validates the accumulation of holes on the surface under visible light irradiation. It also indicates the production of a powerful driving force between the CoO_x_/MnO_x_ and the BV photoanode. Combining IMPS with KPFM demonstrations, we can conclusively verify the highly efficient charge separation/transfer, where there photogenerated holes can migrate from BV to CoO_x_/MnO_x_, correspondingly, these electrons are then passed onto the FTO surface, as also certified by a suitable intermediate energy level for hole transfer (Figure S31, Supporting Information).

### 2.4 Visualization of Charge Transfer Behavior

**Figure 4:**
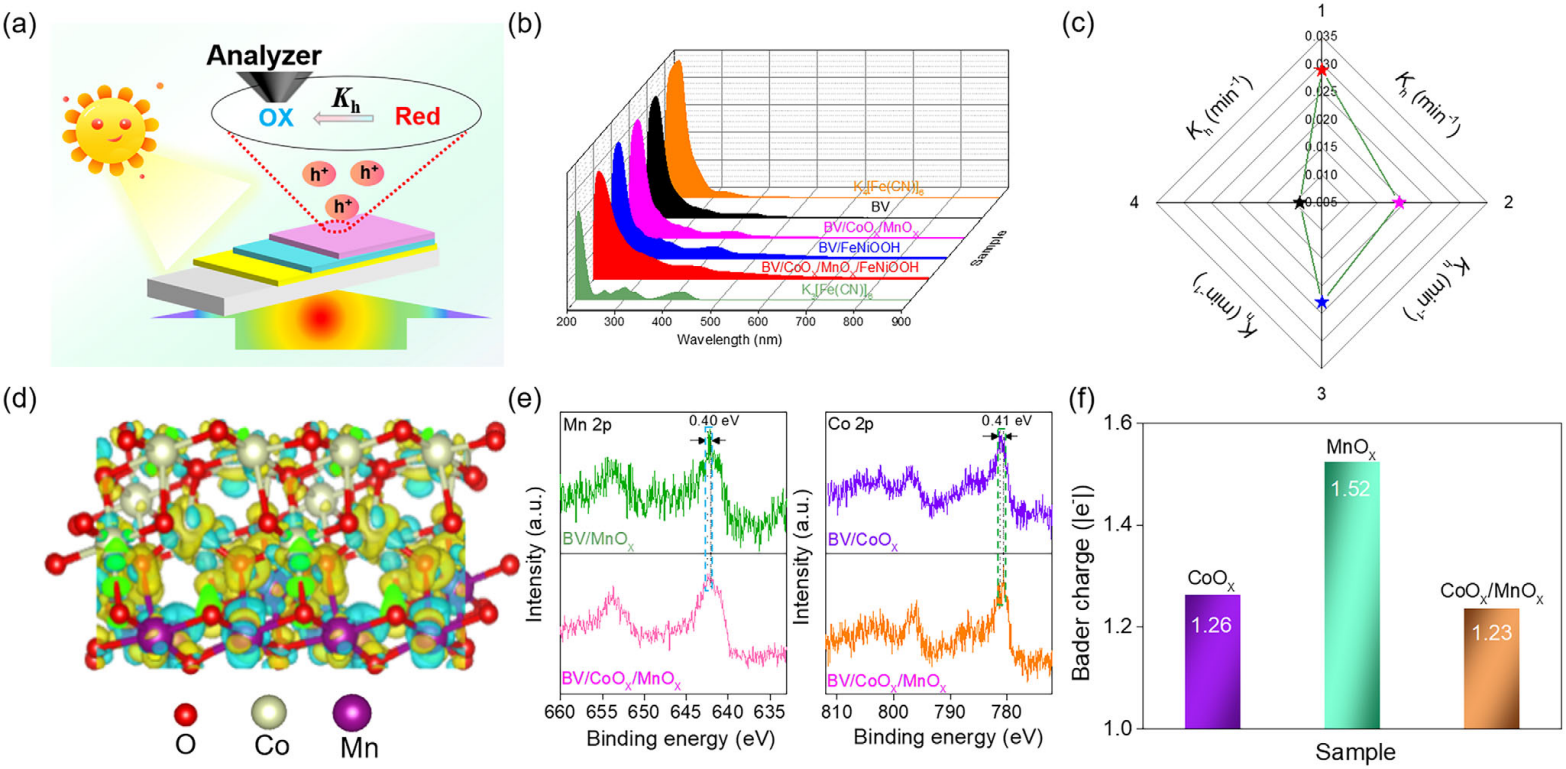
a) Schematic illustration of the UV/vis‐SEC. b) Absorbance of K_4_[Fe(CN)_6_] with BV‐based photoanodes under irradiation. c) Hole transfer kinetics (*K*_h_) of the different samples (1, 2, 3, and 4, respectively, represent BV/CoO_x_/MnO_x_/FeNiOOH, BV/CoO_x_/MnO_x_, BV/FeNiOOH, and BV). d) The charge density difference of CoO_x_/MnO_x_. e) Mn 2p and Co 2p. f) The Bader charge of the different samples.

In spite of the aforementioned positive results, the process through which photogenerated holes undergo long‐distance transport to reach the surface of the electrocatalyst remains a mystery. Accordingly, an in situ ultraviolet/visible spectroelectrochemistry (UV/vis‐SEC) platform (**Figure** **4**a), potassium ferrocyanide (K_4_[Fe(CN)]_6_, donated as Fe^2+^) as a soft probe molecule,^[^ ^9^, ^21^ ^]^ was carried out to visualize the long‐range charge transfer process. The corresponding absorption peaks for Fe^2+^ and K_3_[Fe(CN)_6_] (named as Fe^3+^) are presented separately in Figure 4b. Under AM 1.5 G light irradiation, the characteristic absorption peak of Fe^3+^ (ca. 400 nm) significantly increases as time increases (Figure 4b; Figure S32, Supporting Information), while a decrease in the characteristic absorption peak of Fe^2+^ at 220 nm is also observed. This phenomenon occurs because the photogenerated holes are inclined to move toward the surface and participate in the oxidation reaction of Fe^2+^ (Fe^2+^ + h^+^ = Fe^3+^). Conversely, in the absence of BV‐based photoelectrodes, the peak intensity at 400 or 220 nm remains relatively stable over time (Figure S33, Supporting Information), as the holes are challengeable to reach the surface of the photoanode and initiate the oxidation process. These control experiments further confirm the validity of this platform for quantitatively analyzing the long‐range charge transfer behavior and apparent rate constant (*K*_h_).

As illustrated in Figure S34 (Supporting Information), UV/Vis‐SEC enables the direct quantifying the dynamic of photogenerated holes by measuring the concentrations of Fe^2+^ or Fe^3+^ during the reaction (herein, the *C*_t_ can be converted according to Beer‐Lambert's law^[^ ^22^ ^]^). By fitting above dates, the *K*_h_ for various photoanodes is determined, and reveals a decreasing trend: BV/CoO_x_/MnO_x_/FeNiOOH (0.029 min^−1^) > BV/FeNiOOH (0.023 min^−1^) > BV/CoO_x_/MnO_x_ (0.019 min^−1^) > BV (0.009 min^−1^). Notably, the heterointerface modulated integrated systems of BV/CoO_x_/MnO_x_/FeNiOOH display the highest *K*_h_ value among all samples, being 1.26 times greater than that of BV/FeNiOOH, 1.53 times higher for BV/CoO_x_/MnO_x_, and 3.22 times higher for BV, respectively (Figure 4c; Figure S35, Supporting Information). Together with cyclic voltammetry (CV) result and preliminary analysis of interface dynamics from our group, we find that CoO_x_/MnO_x_ represents an innovative hole transfer mechanism, differing from the previously established passivation and hole storage layers (Figure S36, Supporting Information), which facilitates hole transfer from the BV to the FeNiOOH through a long‐range charge transfer behavior, thereby effectively suppressing interfacial charge recombination. Moreover, the rapid kinetics (*K*_ct_ or *K*_h_) of charge transfer between the SC and TMOOH is dependent on the enhanced PEC performance, which opens a new perspective and offers robust theoretical insights for researchers aiming to optimize the SC/TMOOH interface.

To further clarify the effect of CoO_x_/MnO_x_ on hole transfer, a DFT calculation was performed. The models of CoO_x_, MnO_x_, and CoO_x_/MnO_x_ were developed and illustrated in Figure S37 (Supporting Information). The charge density difference substantiated the electron transfer from MnO_x_ to CoO_x_ through the close contact heterointerface, leading to electrons accumulation on CoO_x_ (more yellow regions, Figure 4d). XPS findings with the obviously negative shift in Mn 2p (Figure 4e) experimentally confirm the role of MnO_x_ in the BV supported CoO_x_/MnO_x_ heterointerface as the electron donor, obtaining an electron deficient Mn (Mn^δ+^) in the CoO_x_/MnO_x_. In contrast, a positive shift in Co 2p XPS peaks (Figure 4e) for CoO_x_/MnO_x_ directly displays the formation of new electron‐rich Co (Co^δ−^) centers accompanied by a progressively increased electron density. We also calculated the Bader charge differences between CoO_x_ and MnO_x_ with the charge polarization matching well with the trend shown in the experimental findings (Figure 4f). Further CV results with varying oxidation‐reduction peaks validate the intrinsic properties of heterointerface (Figure S36, Supporting Information). Above these results highlighting the significant influence of heterointerface in prompting charge polarization. Interestingly, these electron‐rich Co^δ−^ sites created by polarization effect can significantly boost the charge transfer and surface charge separation efficiency. With the introduction of the FeNiOOH layer, XPS results show a negative shift in Mn 2p (Figure S38, Supporting Information). This outcome confirms that the polarization effect of the CoO_x_/MnO_x_ heterointerface in BV/CoO_x_/MnO_x_/FeNiOOH system still plays an important role. For the Mn^δ+^ centers, we will discuss in the following OER process.

### 2.5 Surface Catalytic Analysis

**Figure 5:**
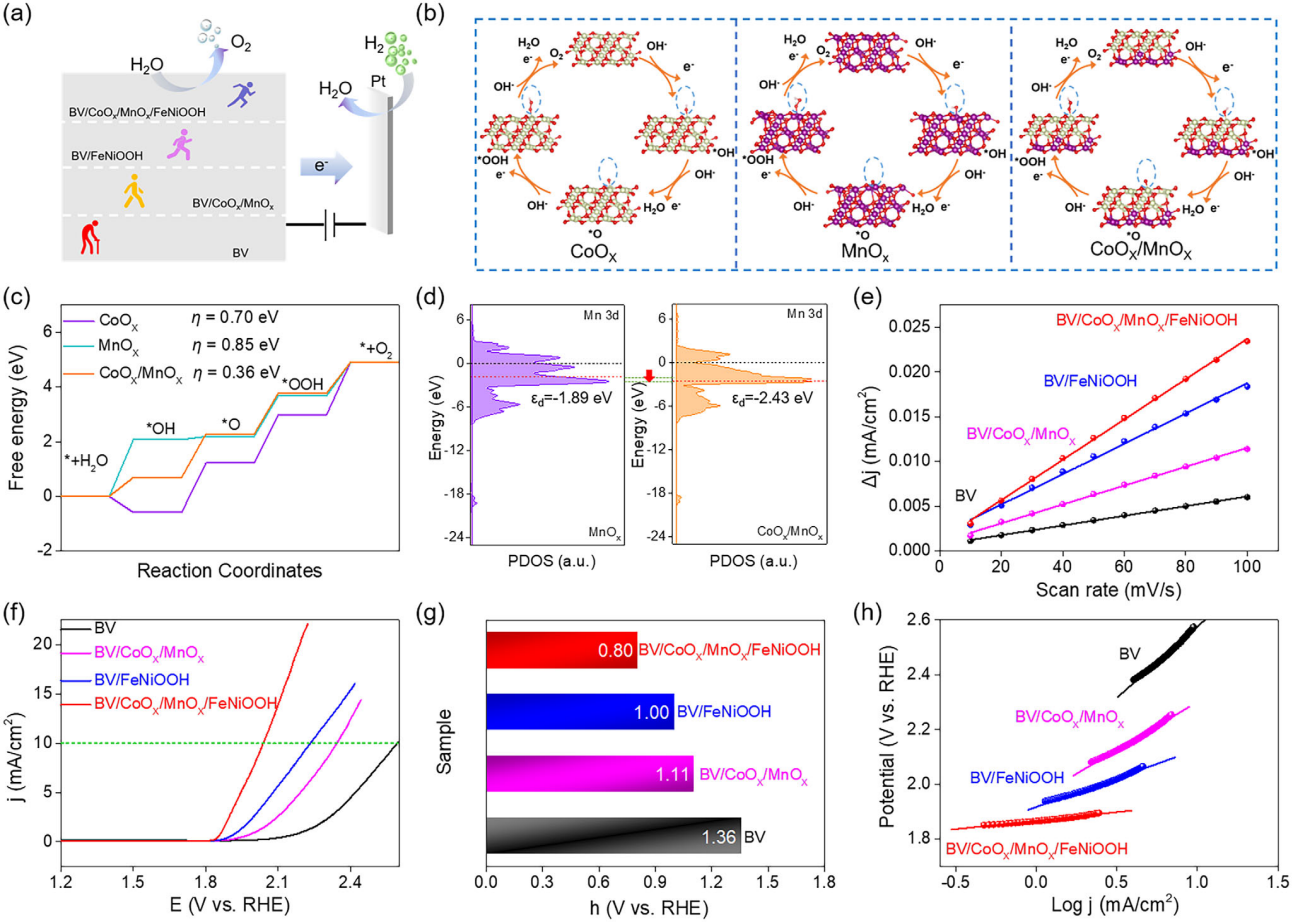
a) The OER process. b) Chemisorption models and c) corresponding Gibbs free energies. d) PDOS for *Mn‐3d* orbitals of the MnO_x_ and CoO_x_/MnO_x_. e) ECSA evaluation. f) LSV curves. g) The overpotential values at a current density of 10 mA/cm^2^. h) Tafel plots.

In addition to effectively weaken interface charge recombination, it is still unknown whether heterointerface influence surface catalytic activity. Therefore, the electrochemical performance of BV, BV/CoO_x_, BV/MnO_x_, BV/CoO_x_/MnO_x_, BV/FeNiOOH, and BV/CoO_x_/MnO_x_/FeNiOOH toward OER was investigated through a typical three‐electrode system (**Figure** **5**a). Figure S39a (Supporting Information) shows the *iR* corrected linear sweep voltammetry (LSV) curves of all samples. Figure S39b (Supporting Information) presents a bar diagram illustrating the overpotentials needed for each catalyst to reach a given current density. As for surface OER, the BV/CoO_x_/MnO_x_ catalyst has also displayed superior OER activity with an ultralow over‐potential of 1.11 V at 10 mA cm^−2^ compared with other catalyst (Figure S39b, Supporting Information). Moreover, Tafel slopes can be used to reveal the surface catalytic reaction kinetics (defined as *K*_ec_), where the corresponding *K*_ec_ (Figure S39c, Supporting Information) of the BV/CoO_x_/MnO_x_ catalyst (349.33 mV dec^−1^) outperform their counterparts including BV (520.80 mV dec^−1^), BV/CoO_x_ (373.23 mV dec^−1^), and BV/MnO_x_ (429.26 mV dec^−1^). According on Nyquist plots, the *R*_ct_ of BV/CoO_x_/MnO_x_ is smaller than that of other samples, suggesting a boosted OER charge transfer kinetics (Figure S39d and Table S5, Supporting Information). To assess the electrochemically active surface area (ECSA, Figure S40, Supporting Information), the double layer capacitance (C_dl_) values for each catalyst were determined. It can be seen from the Figure S41 (Supporting Information) that the BV/CoO_x_/MnO_x_ catalyst possesses a higher C_dl_ value of 105.58 µF cm^−2^ and thus greater OER activity in contrast to other samples.

After verifying the outstanding electrocatalytic OER performance of BV/CoO_x_/MnO_x_, we also proceeded to investigate its structure by DFT simulation. Figure 5b illustrates the elementary reaction steps involved in the OER process and these include different intermediates (*OH, *O, and *OOH). Based on the findings from the calculations, a big energy barrier (*η*) of 0.85 eV was achieved for the MnO_x_ with the generation of *OH serving as the potential rate‐determining step (RDS). Similarly, the CoO_x_ system exhibits a reduced energy barrier with a RDS of 0.70 eV for the transition from *OOH to O_2_, in contrast to the MnO_x_. Interestingly, a lower energy barrier of CoO_x_/MnO_x_ is obtained after simultaneous introduction of CoO_x_ and MnO_x_. The energy barrier of CoO_x_/MnO_x_ for the RDS of the transition from *OH to *O is significantly decreased to 0.36 eV with a standard potential of 0 V (Figure 5c).

Moreover, the polarization effect results in a downshift of the d‐band center of Mn atoms in CoO_x_/MnO_x_ (−2.43 eV) compared to that in MnO_x_ (−1.89 eV, Figure 5d). This alteration leads to an increased occupancy of antibonding states, subsequently reducing the adsorption of oxygen‐containing species and facilitating O_2_ desorption.^[^ ^23^ ^]^ More importantly, the polarization effect induces more Mn^δ+^ species (for more details refer to Figure S9c,d, Supporting Information), and further promotes the dynamics of oxygen evolution. In summary, both experimental and computational results have validated the polarization effect can enhance the PEC water splitting performance by facilitating interfacial charge transfer and improving surface catalysis. Of note, after decoration of FeNiOOH, which is the best catalyst for OER, the OER performance of the integrated system BV/CoO_x_/MnO_x_/FeNiOOH will be further improved, as indicated by the findings from the ECSA, LSV, Tafel slope, and EIS analyses (Figure 5e–h; Figures S42 and S43a and Table S6, Supporting Information), which contribute to interfacial interaction (FeNiOOH, Figure S43b, Supporting Information), causing electron transfer and resulting in higher conductivity (Figures S39d and S43a, Supporting Information), which is beneficial for boosting the oxygen evolution dynamics.^[^ ^24^ ^]^

### 2.6 Universality Analysis

**Figure 6:**
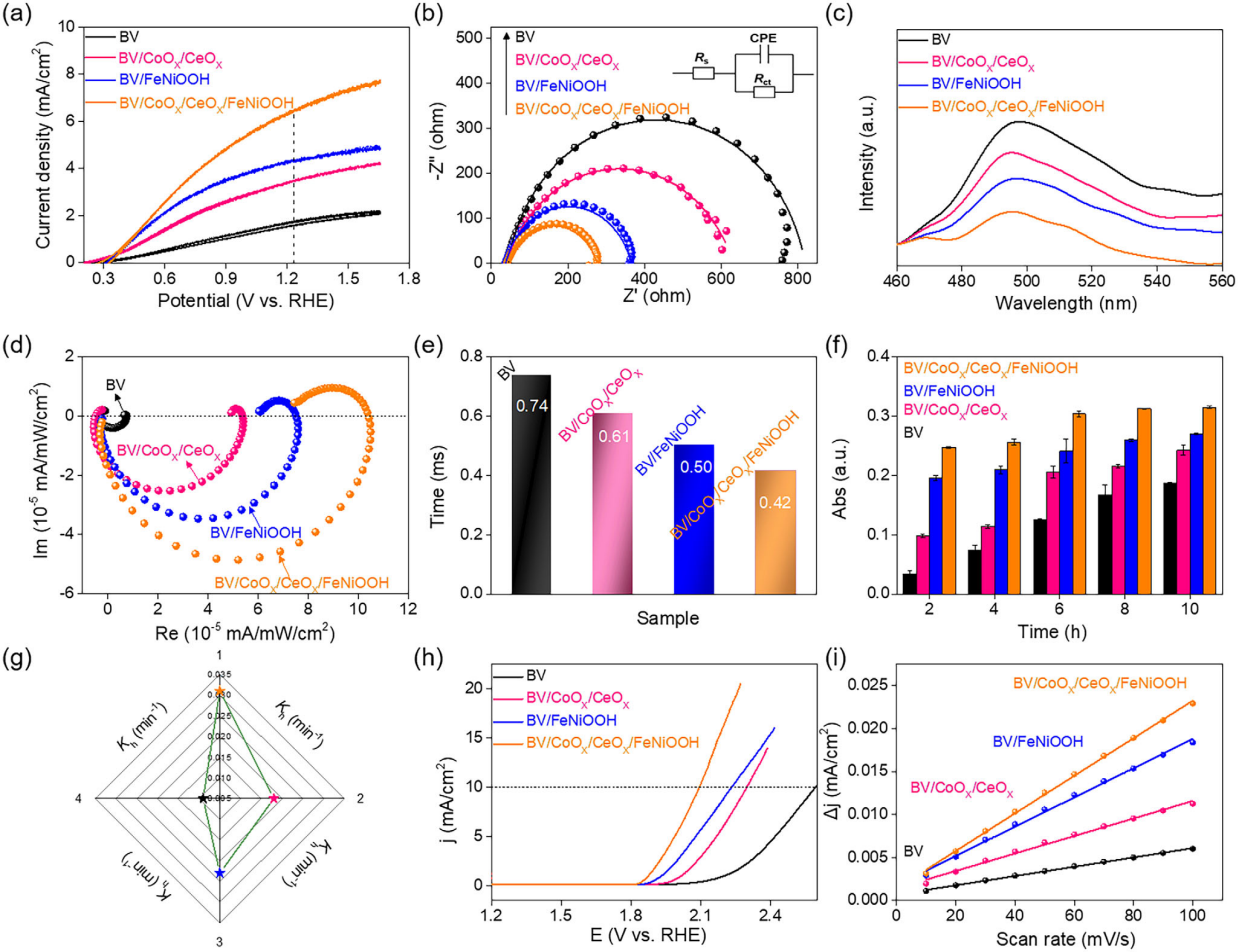
a) LSV curves. b) EIS. c) PL spectra of different samples. d) IMPS response and e) transient time of different samples. f) Results of time‐dependent absorption spectra. g) Hole transfer kinetics (1, 2, 3, and 4, respectively, represent BV/CoO_x_/CeO_x_/FeNiOOH, BV/CoO_x_/CeO_x_, BV/FeNiOOH, and BV). h) LSV curves. i) ECSA evaluation.

It is also very interesting that a charge polarization of the CoO_x_/CeO_x_ displays a remarkable effect on the charge separation and surface catalysis (it should be noted that the similar polarization effect can be revealed by different redox behaviors, Figure S44, Supporting Information). Typically, the LSV findings indicate that the BV/CoO_x_/CeO_x_/FeNiOOH photoanode demonstrates superior photocurrent densities (6.45 mA cm^−2^ at 1.23 V_RHE_) when compared to BV based counterparts, respectively (**Figure** **6**a; Figures S45–S50a, Supporting Information). The anticipated PEC performance is corroborated by the results from ABPE (Figures S50b and S51a, Supporting Information), EIS (Figure 6b; Figure S50c and Table S7, Supporting Information), IPCE (Figure S51b, Supporting Information), and PL (Figure 6c).

To further validate the suppression of interface charge recombination attributed to polarization effect by inducing rapid hole transfer, comprehensive studies were performed on the photoanodes utilizing UV–vis diffuse reflectance spectroscopy (Figure S52, Supporting Information), IMPS, and UV–vis/SEC. As illustrated in Figure 6d,e, Figure S53, and Table S8 (Supporting Information), the introduction of the heterointerface results in *K*_ct_ being significantly greater than that of *K*_rc_, following the trend: BV/CoO_x_/CeO_x_/FeNiOOH > BV/FeNiOOH > BV/CoO_x_/CeO_x_ > BV, which aligns with the *K*_h_ results (Figure 6f,g; Figures S54 and S55, Supporting Information).

The polarization curve reveals that BV/CoO_x_/CeO_x_/FeNiOOH has a lower overpotential, *K*_ec_, EIS, and higher C_dl_ value in comparison to BV/FeNiOOH, BV/CoO_x_/CeO_x_, BV/CoO_x_, BV/CeO_x_, and BV (Figure 6h,i; Figures S56–S59 and Table S9, Supporting Information). This suggests that the development of the charge polarization enhances the rapid kinetics of the OER process in BV/CoO_x_/CeO_x_/FeNiOOH. Taken together, the well‐designed charge polarization of heterointerface construction can simultaneously modulate charge transfer behavior at photoanode/electrocatalyst/electrolyte interfaces.

## 3 Conclusion

In this work, we have demonstrated that the heterointerface charge polarization effect can be successfully extended to SC/TMOOH system for PEC water splitting. Notably, the optimized BV/CoO_x_/MnO_x_/FeNiOOH photoanode exhibits a substantial photocurrent density of 6.75 mA cm^−2^ at 1.23 V versus RHE, accompanied with an excellent photostability. By the aid of IMPS, SPV, KPFM, in situ UV–vis/SEC, OER, and DFT calculations, we confirmed that the expected performance was attributed to these electron‐rich Co^δ−^ sites induced by polarization effect, significantly boosting the long‐range charge transfer dynamics. Specifically, BV/CoO_x_/MnO_x_/FeNiOOH exhibits the highest hole transfer kinetic rate constant (1.26 × 10^2^ s^−1^), which is over eight times greater than that of BV (0.15 × 10^2^ s^−1^). Additionally, the Mn^δ+^ sites can further optimize the TMOOH/electrolyte interface aiming to reduce the adsorption of oxygen‐containing intermediates. And the *K*_ec_ of BV/CoO_x_/MnO_x_/FeNiOOH is decreased from 520.80 to 62.06 mV dec^−1^. More interestingly, this method was successfully extended to CoO_x_/CeO_x_ heterointerface and exhibited high PEC performance. This study has pointed out a new polarization strategy to modulated the local charge density in heterointerface to generate more active sites, realizing the highly efficient PEC water splitting.

## Conflict of Interest

The authors declare no conflict of interest.

## Supporting information



Supporting Information

## Data Availability

The data that support the findings of this study are available from the corresponding author upon reasonable request.

## References

[1] a) X. Li , Z. Wang , A. Sasani , A. Baktash , K. Wang , H. Lu , J. You , P. Chen , P. Chen , Y. Bao , S. Zhang , G. Liu , L. Wang , Nat. Commun. 2024, 15, 9127; 39443493 10.1038/s41467-024-53426-8 PMC11499990

[2] a) A. Kormányos , E. Kecsenovity , A. Honarfar , T. Pullerits , C. Janáky , Adv. Funct. Mater. 2020, 30, 2002124; 32774199 10.1002/adfm.202002124 PMC7405979

[3] a) X. Chen , X. Li , Y. Peng , H. Yang , Y. Tong , M.‐S. Balogun , Y. Huang , Adv. Funct. Mater. 2025, 35, 2416091;

[4] a) F. A. L. Laskowski , M. R. Nellist , J. Qiu , S. W. Boettcher , J. Am. Chem. Soc. 2019, 141, 1394; 30537811 10.1021/jacs.8b09449

[5] a) S. Wang , P. Chen , Y. Bai , J.‐H. Yun , G. Liu , L. Wang , Adv. Mater. 2018, 30, 1800486; 10.1002/adma.201800486 29602201

[6] B. Zhang , X. Huang , Y. Zhang , G. Lu , L. Chou , Y. Bi , Angew. Chem., Int. Ed. 2020, 59, 18990. 10.1002/anie.202008198 32666681

[7] a) T. W. Kim , K.‐S. Choi , Science 2014, 343, 990; 24526312 10.1126/science.1246913

[8] K. Zhang , B. Jin , C. Park , Y. Cho , X. Song , X. Shi , S. Zhang , W. Kim , H. Zeng , J. H. Park , Nat. Commun. 2019, 10, 2001. 31043598 10.1038/s41467-019-10034-1 PMC6494903

[9] X. Ning , B. Lu , Z. Zhang , P. Du , H. Ren , D. Shan , J. Chen , Y. Gao , X. Lu , Angew. Chem., Int. Ed. 2019, 58, 16800. 10.1002/anie.201908833 31486209

[10] a) S. Ye , C. Ding , R. Chen , F. Fan , P. Fu , H. Yin , X. Wang , Z. Wang , P. Du , C. Li , J. Am. Chem. Soc. 2018, 140, 3250; 29338218 10.1021/jacs.7b10662

[11] a) W. Ding , M. Feng , Z. Zhang , F. Fan , L. Chen , K. Zhang , J. Colloid Interface Sci. 2025, 682, 1140; 39671948 10.1016/j.jcis.2024.12.028

[12] L. Xu , M. Li , F. Zhao , J. Quan , X. Ning , P. Chen , Z. An , X. Chen , Appl. Catal. B Environ. Energy 2024, 359, 124503.

[13] a) X. Sun , J. Chen , J. Zhai , H. Zhang , S. Dong , J. Am. Chem. Soc. 2022, 144, 23073; 36503222 10.1021/jacs.2c10445

[14] H. Wu , L. Zhang , A. Du , R. Irani , R. van de Krol , F. F. Abdi , Y. H. Ng , Nat. Commun. 2022, 13, 6231. 36266344 10.1038/s41467-022-33905-6 PMC9585101

[15] X. Ning , D. Yin , Y. Fan , Q. Zhang , P. Du , D. Zhang , J. Chen , X. Lu , Adv. Energy Mater. 2021, 11, 2100405.

[16] Q. Tan , X. Li , B. Zhang , X. Chen , Y. Tian , H. Wan , L. Zhang , L. Miao , C. Wang , Y. Gan , J. Jiang , Y. Wang , H. Wang , Adv. Energy Mater. 2020, 10, 2001050.

[17] K. Tian , L. Jin , A. Mahmood , H. Yang , P. An , J. Zhang , Y. Ji , Y. Li , D. Li , S. Liu , J. Yan , Adv. Funct. Mater. 2024, 34, 2410548.

[18] a) J. Yang , C. Deng , Y. Lei , M. Duan , Y. Yang , X. Chen , S. Yang , J. Li , H. Sheng , W. Shi , C. Chen , J. Zhao , Angew. Chem., Int. Ed. 2024, 62, 202416340; 10.1002/anie.202416340 39330922

[19] a) F. Li , H. Yang , Q. Zhuo , D. Zhou , X. Wu , P. Zhang , Z. Yao , L. Sun , Angew. Chem., Int. Ed. 2021, 60, 1976; 10.1002/anie.202011069 PMC7894348 33051952

[20] M. Li , M. Saqib , L. Xu , C. Li , J. Quan , X. Ning , P. Chen , Q. Weng , Z. An , X. Chen , J. Mater. Chem. A 2024, 12, 19259.

[21] Y. Ma , C. A. Mesa , E. Pastor , A. Kafizas , L. Francàs , F. Le Formal , S. R. Pendlebury , J. R. Durrant , ACS Energy Lett. 2016, 1, 618.

[22] S. Pishgar , S. Gulati , J. M. Strain , Y. Liang , M. C. Mulvehill , J. M. Spurgeon , Small Methods 2021, 5, 2100322. 10.1002/smtd.202100322 34927994

[23] W. Huang , J. Li , X. Liao , R. Lu , C. Ling , X. Liu , J. Meng , L. Qu , M. Lin , X. Hong , X. Zhou , S. Liu , Y. Zhao , L. Zhou , L. Mai , Adv. Mater. 2022, 34, 2200270. 10.1002/adma.202200270 35278337

[24] a) M. Chen , H. Li , C. Wu , Y. Liang , J. Qi , J. Li , E. Shangguan , W. Zhang , R. Cao , Adv. Funct. Mater. 2022, 32, 2206407;

