# Supplementary material for: Unlocking the Potential of Photoelectrochemical Water Splitting via Heterointerface Charge Polarization
Source: Adv Sci (Weinh). 2025 Apr 17;12(26):2502384. doi: 10.1002/advs.202502384 (PMC12245032; doi:10.1002/advs.202502384)
Supplement: Supplementary file 1 — Supporting Information [file ADVS-12-2502384-s001.docx]

**Supporting Information**

**Unlocking the Potential of Photoelectrochemical Water Splitting via Heterointerface Charge Polarization**

Li Xu, Xingming Ning*, Jingjing Quan, Chenglong Li, Lan Yao, Qiang Weng, Pei Chen*, Zhongwei An, Xinbing Chen*

Key Laboratory of Applied Surface and Colloid Chemistry (MOE), Shaanxi Key Laboratory for Advanced Energy Devices, Shaanxi Engineering Laboratory for Advanced Energy Technology, International Joint Research Center of Shaanxi Province for Photoelectric Materials Science, School of Materials Science and Engineering, Shaanxi Normal University, Xi’an, 710119, PR China.

Email: ningxingming@snnu.edu.cn (X. Ning), chenpei@snnu.edu.cn (P. Chen), and chenxinbing@snnu.edu.cn (X. Chen).

**Experimental Procedures**

**Chemical reagents and Instruments:** Fluorine-doped SnO_2_ (FTO) substrates (14 Ω per square) were purchased from Wuhan Jinge Solar Energy Technology Co., Ltd. And before using, the FTO substrates were ultrasonically cleaned for 15 min each in deionized water, ethanol, and acetone, respectively. Bismuth nitrate pentahydrate (Bi(NO_3_)_3_·5H_2_O, 98.0%) and Manganese nitrate tetrahydrate (Mn(NO_3_)_2_·4H_2_O, 98.0%) were obtained from Alfa Aesar (China) Chemicals Co., Ltd. Sodium sulfite (Na_2_SO_3_, ≥ 97.0%), p-Benzoquinone (≥ 98.0%), Nickel(II) nitrate hexahydrate (Ni(NO_3_)_2_·6H_2_O, ≥ 98.00%), Potassium hydroxide (KOH, AR), Potassium chloride (KCl, ≥ 99.5%), Nitric acid (HNO_3_, AR), Ethanol absolute (C_2_H_6_O, ≥ 99.7%), and Acetone (C_3_H_6_O, ≥ 99.5%) were purchased from Sinopharm Chemical Reagent Co., Ltd. Vanadyl acetylacetonate (VO(acac)_2_, 98.0%), Dimethyl sulfoxide (DMSO, > 99.0%), Potassium iodide (KI, AR), Potassium ferrocyanide (K_4_Fe(CN)_6_, ≥ 99.0%), Potassium ferricyanide (K_3_Fe(CN)_6_, ≥ 99.5% ), Boric acid (H_3_BO_3_, 99.0%), Iron sulfate heptahydrate (FeSO_4_·7H_2_O, ≥ 99.0%), and Cobalt nitrate hexahydrate (Co(NO_3_)_2_·6H_2_O, 99.0%) were purchased from Shanghai Aladdin Biochemical Technology Co., Ltd. Cerium(Ⅲ) nitrate hydrate (Ce(NO_3_)_3_·xH_2_O, 99.998%) was obtained from Shanghai Macklin Biochemical Co., Ltd. All the reagents were used without any purification process.

**Preparation of BV photoanodes.** The BV photoanodes were obtained by following method. Dissolved 3.32 g of KI in 50 mL of deionized water, and then slowly added 0.97 g of Bi(NO_3_)_3_·5H_2_O to the above solution. The pH value of 1.70 was modulated by using HNO_3_. And then the solution was mixed with 20 mL of ethanol (99.99%) including 0.50 g p-Benzoquinone. Stirred on the mixer for 5 min to form the precursor solution. And electrodeposition onto FTO using a three electrode system (-0.1 V vs Ag/AgCl for 300 s). Subsequently, the solution containing 4 mL of DMSO with 0.21 g vanadyl acetylacetonate was dripped onto these BiOI films, and heated to 450 °C in an air atmosphere and hold for 2 h (ramp rate 2 °C/min). Finally, the prepared photoanodes were soaked in 1 M K_3_BO_3_ solution for 30 min. And the obtained BV photoanodes were rinsed with deionized water and dried in air.

**Preparation of CoO_x_/MnO_x_ and CoO_x_/CeO_x_ nanosheets.** Firstly, using electrodeposition to load Co(OH)_2_ electrodes onto the surface of FTO. Specifically, 16 mM Co(NO_3_)_2_·6H_2_O was prepared in deionized water. And the deposition was carried out at constant potential of -0.4 V vs Ag/AgCl for 50 s. And then the Mn(OH)_x_ and Ce(OH)_x_ electrodes were prepared for electrodeposition onto the surface of Co(OH)_2_, respectively. 16 mM Mn(NO_3_)_2_·6H_2_O and Ce(NO_3_)_3_·xH_2_O were dissolved in deionized water. And the Mn(OH)_x_ and Ce(OH)_x_ electrodes deposition were performed at constant potential of -0.4 V vs Ag/AgCl for 50 s. The obtained Co(OH)_2_/Mn(OH)_x_ and Co(OH)_2_/Ce(OH)_x_ photoanodes were rinsed with deionized water and dried in ambient air, and heated in a muffle furnace at 300 °C for 2 h (ramp rate 2 °C/min).

**Preparation of BV/CoO_x_/MnO_x_ and BVCoO_x_/CeO_x_ photoanodes.** The BV/CoO_x_/MnO_x_ and BVCoO_x_/CeO_x_ photoanodes were deposited onto the surface of BV photoanodes by the above process.

**Preparation of BV/CoO_x_/MnO_x_/FeNiOOH and BVCoO_x_/CeO_x_/FeNiOOH photoanodes.** The BV/CoO_x_/MnO_x_/FeNiOOH and BVCoO_x_/CeO_x_/FeNiOOH photoanodes were prepared by a simple photoelectrodeposition method. The electrolyte was taken through a mixed aqueous solution of Ni(NO_3_)_2_·6H_2_O (75 mM) and FeSO_4_·7H_2_O (25 mM). The photoelectrodeposition voltage set for the electrochemical workstation was 0.3 V vs Ag/AgCl for 600 s.

**Materials characterizations.** Transmission electron microscopy (TEM) and high-resolution TEM (HR-TEM, Tecnai G2 F30) measurements were performed at 200 kV and elements mapping.The morphology of all samples were observed by field emission scanning electron microscopy (SEM, SU8020). X-ray diffraction analysis (XRD, SmartLab (9)) measurements were carried out at 3KW. X-ray photoelectron spectroscopy (XPS, Escalab Xi^+^) were performed by using Al Kα. Raman spectroscopy (inVia Reflex) were measured equipped with 532 nm laser. Photoluminescence (PL) spectra were measured using FL 8500 (excitation of 355 nm). Time-resolved photoluminescence (TRPL) spectra were tested on Fluorolog-QM (HORIBA). UV/Vis spectroscopy were taken on a UV-3600 from 400 to 600 nm (Shimadzu). The production of H_2_ and O_2_ was detected with a gas chromatograph (9790 II, Fuli, Zhejiang) every 10 min. Kelvin probe force microscopy (KPFM) was probed by Oxford Instruments Asylum Research/Cypher S. The contact potentialdiﬀerence (CPD), namely, surface potential, of the sample was measured using KPFM in the contact mode. The excitation light source is 405 nm laser. The surface photovoltage (SPV) is tested using an autonomous setup (Bruker Dimension ICON-SPVM101 coupling system).

**Electrochemical measurements.** By using three-electrode system (all samples as work electrode (WE), Ag/AgCl and platinum plates were used as reference electrodes and auxiliary electrodes, respectively.), electrochemical measurements were conducted on CHI760E and the electrolyte used 1M K_3_BO_3_ (pH 9.5). The cyclic voltammertry curves of the different samples were obtained with different scan rates, such as 10-100 mV/s. Oxygen evolution reaction (OER) measurement was carried out under dark conditions, with 90% *iR* compensation applied.

**Photoelectrochemical measurements.** Photoelectrochemical tests of all photoanodes were composed of a standard three-electrode configuration (scan rate of 50 mV/s). The light source containingn AM 1.5 G filter (PLS-SXE300D, 300 W Xenon arc lamp) was used as a simulated solar to calibrate AM 1.5 G, corresponding to 100 mW/cm^2^. All the potentials vs RHE were converted from the potentials vs Ag/AgCl according to the Nernst equation: *E*_(RHE)_ = *E*_(Ag/AgCl)_ + 0.0591 pH + 0.197. The following photoelectrochemical measurements were carried out on Autolab M204. The EIS was conducted by 0 V vs Ag/AgCl. In the equivalent randles circuit, *R*_ct_, *R*_s_, and CPE represent charge transfer resistance, series resistance, and constant phase element, respectively. IMPS measurements were performed by photoechem system (LED, 470 nm). The frequency range measured by IMPS response diagram is from 10 KHz to 0.1 Hz. And the *I-t* curves were conducted by applying same potential and light source (LED, 470 nm) with IMPS.

The $\text{η}_{\text{sep}}$ of different samples can be achieved by the equation:^[1]^

$$\text{η}_{\text{sep}}\left( \text{\%} \right)\text{=}\frac{\text{J}_{\text{(H}_{\text{2}}\text{O)}}}{\text{J}_{\text{(N}{\text{a}_{\text{2}}\text{SO}}_{\text{3}})}}\text{×100}$$

Where the values of $\text{J}_{\text{(H}_{\text{2}}\text{O)}}$ and $\text{J}_{\text{(N}{\text{a}_{\text{2}}\text{SO}}_{\text{3}})}$ represent the photocurrent density tested in 1M K_3_BO_3_ (pH 9.5) without and with Na_2_SO_3_, respectively.

The calculation formula of ABPE is as follows:^[2]^

$$\text{ABPE=}\frac{\text{J}\text{×}\left( \text{1.23-}\text{V}_{\text{bis}} \right)}{\text{P}_{\text{light}}}\text{×100}$$

where *J* (mA/cm^2^) is the photocurrent density, *V*_bis_ (vs. RHE) is the applied bias voltage, and *P*_light_ (100 mW/cm^2^) is AM 1.5 G illumination power density.

IPCE is calculated by the following equation:^[3]^

$$\text{IPCE=}\frac{\text{I }\text{×1240}}{\text{P}\text{×}\text{λ}}\text{×100}$$

Where *I* (mA/cm^2^) presents the current density from set wavelength, *λ* (nm) and *P* (mW/cm^2^) are the wavelength and power density of the incident light at specific wavelength, respectively.

**Ultraviolet/visible-spectroelectrochemistry (UV/vis-SEC) measurements.** UV/vis-SEC measurements were conducted by our home-built equipment. Herein, the substrate electrodes modified with different samples (BV, BV/FeNiOOH, BV/CoO_x_/MnO_x_, BV/CoO_x_/MnO_x_/FeNiOOH, BV/CoO_x_/CeO_x_, and BV/CoO_x_/CeO_x_/FeNiOOH) were regarded as WE. Meanwhile, Ag/AgCl and platinum wire were treated as reference electrode and auxiliary electrode, respectively. In addition, a soft probe molecule (i.e., K_4_[Fe(CN)_6_]) is employed as electrolyte. Under light irradiation (AM 1.5 G), BV will generate electrons and holes, and these photogenerated electrons will directly migrate to FTO substrate, while the corresponding holes will transfer from the valance band (VB) of BV to the surface of integrated photoanode. Owing to the redox potential of [Fe(CN)_6_]^3-^/[Fe(CN)_6_]^4-^ is 0.36 V_RHE_, therefore, these accumulated holes are easily to oxidize [Fe(CN)_6_]^4-^ to [Fe(CN)_6_]^3-^, resulting in concentration changes of electrolyte in different systems. During the reaction, the different of concentration can be monitoring with UV/vis-SEC by observed new peaks and changes in the position or intensity from absorbance maximum. Where absorbance can be closely corresponded to the concentration according to the Beer-Lambert Law.

**Computational method:** The Vienna Ab Initio Simulation Package (VASP) was provided with the simulation calculations.^[4]^ In the DFT modeling, the CoO_x_ and MnO_x_ slab structures were constructed based on the (311) facets of CoO_x_ and MnO_x_, with varying Co, Mn, and O atomic ratios (lattice mismatch<5%). Using these optimized slabs, we further built CoO_x_/MnO_x_ heterolayers. The optimized slabs were constrained within a lattice constant of a =11.45228 Å b = 9.91800 Å, c = 21.28886 Å for CoO_x_, MnO_x_, and CoO_x_/MnO_x_, respectively. To model the catalytic OER process, we fixed the bottom layer while allowing the upper layer to relax. The generalized gradient approximation (GGA) showed in the form of the Perdew-Burke-Ernzerhof (PBE) function was employed to calculate exchange-correlation interactions.^[5]^ The energy cutoff of 450 eV and the Monkhorst-Pack k-point of 3 × 3 × 1 were set in this work. DFT-D3 method, developed by grimme et al.^[6]^ was applied to consider the long-range dispersion interactions between adsorbates and surface. In addition, when the electronic self-consistent iteration and force were reached separately 10^-5^ eV and 0.02 eV/Å, the geometry optimization and energy calculation are completed.

The Gibbs free energy (ΔG) for OER process was calculated as:^[7]^

Δ*G =* Δ*E +* Δ*E_ZPE_ –T*Δ*S*

Where ΔE is the energy change, Δ*E_ZPE_* and Δ*S* present the energy difference and entropy between the adsorbed state and gas phase, and T is the temperature (298.15 K). *E_ZPE_* and the entropies of free molecules were obtained by summing the vibration frequency of the designed model in the above reaction system: *E_ZPE_* = 1/2∑h*v*_i_ and NIST database, respectively.

The OER process is divide into the four fundamental reactions as following:

（1）*+H_2_O → *OH + H^+^ + e^-^

（2）*OH → *O + H^+^ + e^-^

（3）*O + H_2_O → *OOH + H^+^ + e^-^

（4）*OOH → O_2_ + H^+^ + e^-^ + *

OOH*, O*, and OH * present the OOH, O and OH moieties on the adsorption site.

Results and Discussion


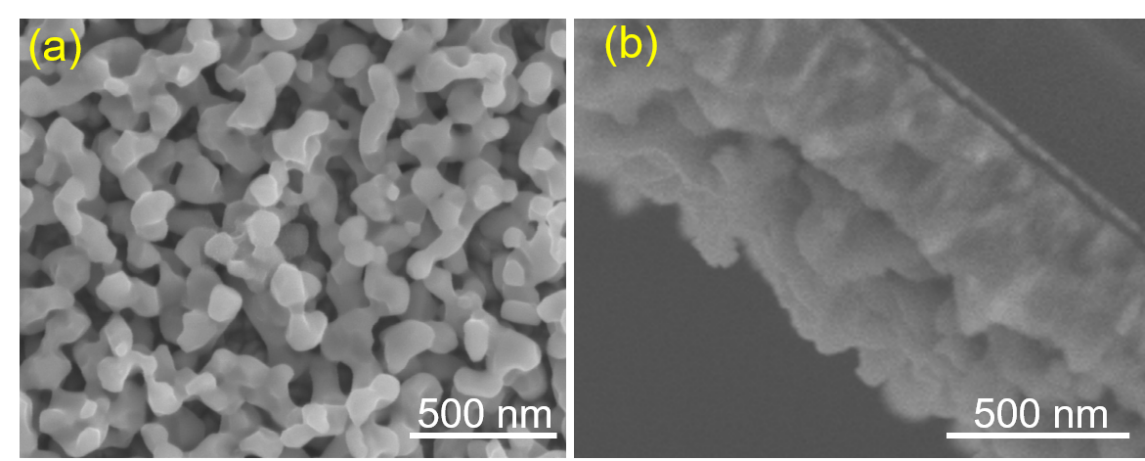


**Figure S1.** (a) Scanning electron microscopy (SEM) image and (b) top view SEM image of bare BV.

Figure S1 illustrates that the nanoporous BV nanofilm is uniformly spread across the surface of the FTO.


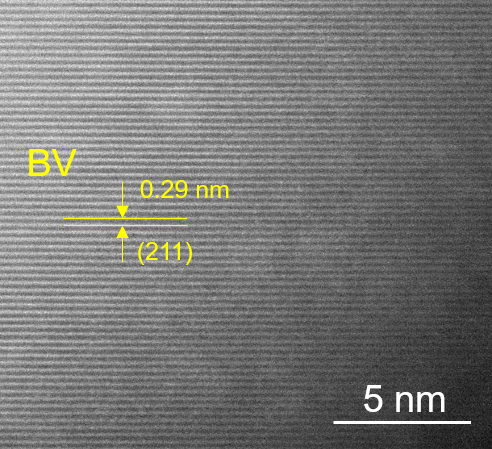


**Figure S2.** High-resolution transmission electron microscopy (HR-TEM) image of BV.

Figure S2 distinctly shows that the nanocrystal possesses a lattice spacing of 0.29 nm that corresponds to the (211) plane of BV phase.


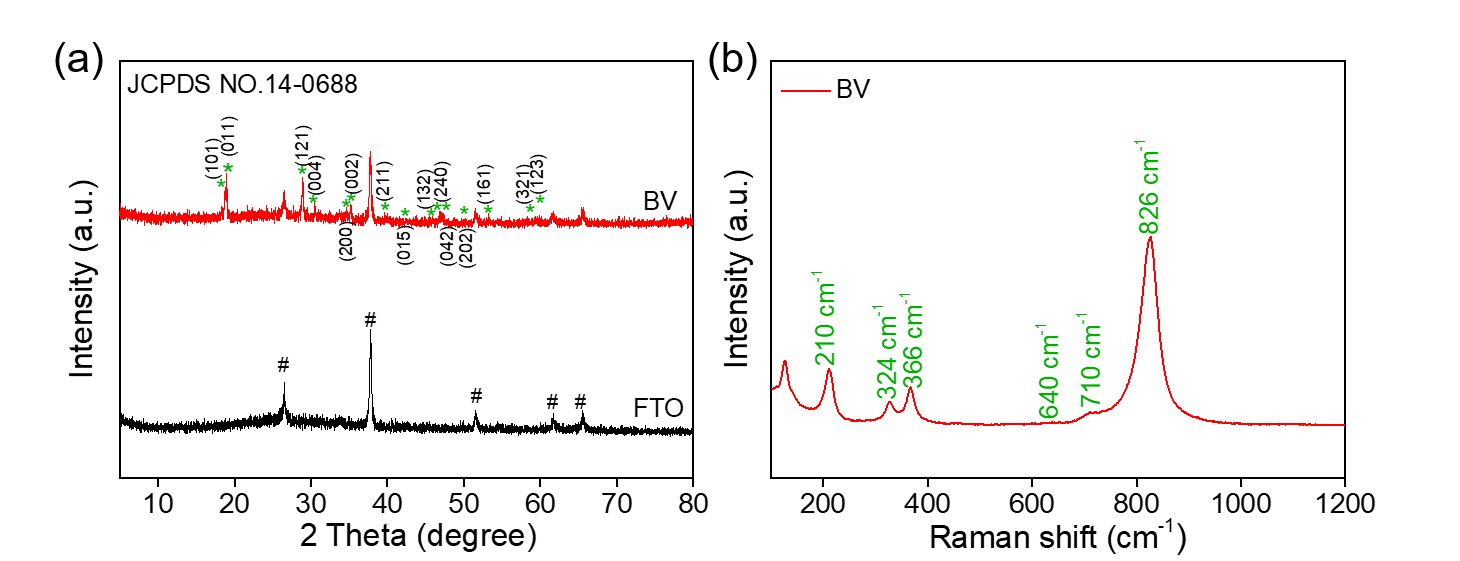


**Figure S3.** (a) X-ray diffraction (XRD) patterns of FTO and bare BV. (b) Raman spectra of BV.

The diffraction peaks shown in Figure S3 can be assigned to monoclinic BV (JCPDS PDF #14-0688) and FTO, respectively.


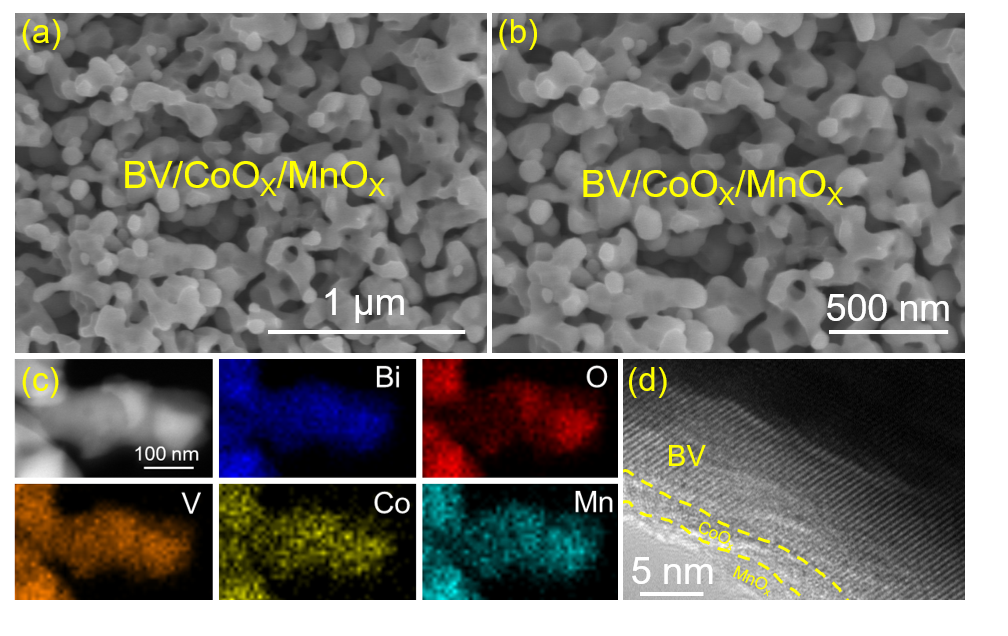


**Figure S4.** (a,b) SEM images, (c) element mappings, and (d) HR-TEM image of BV/CoO_x_/MnO_x_.

EDS elemental mapping images provide additional evidence for the presence of CoO_x_/MnO_x_ on BV.


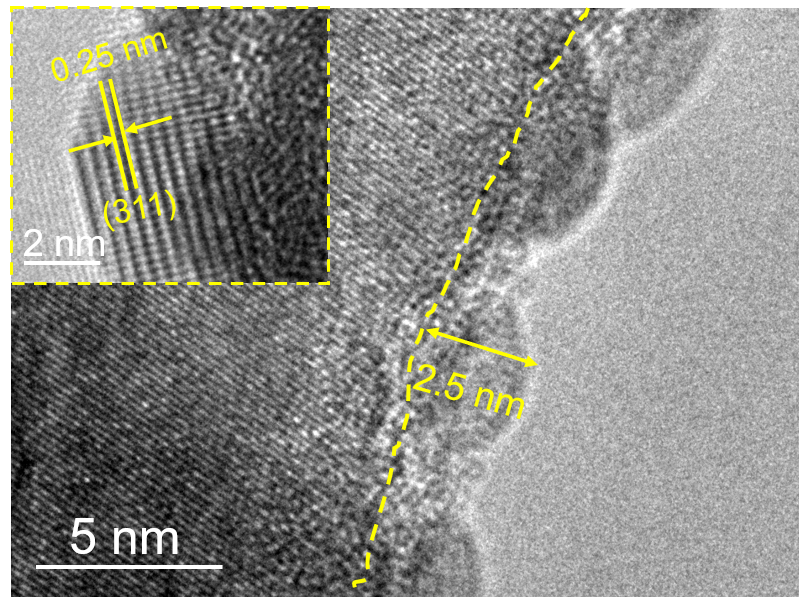


**Figure S5.** HR-TEM images of BV/CoO_x_.

From Figure S5, we can see that CoO_x_ with a thickness of about 2.5 nm is loaded on the surface of BV. Moreover, a lattice spacing of 0.25 nm corresponds to the (311) plane of CoO_x_ phase.^[8]^


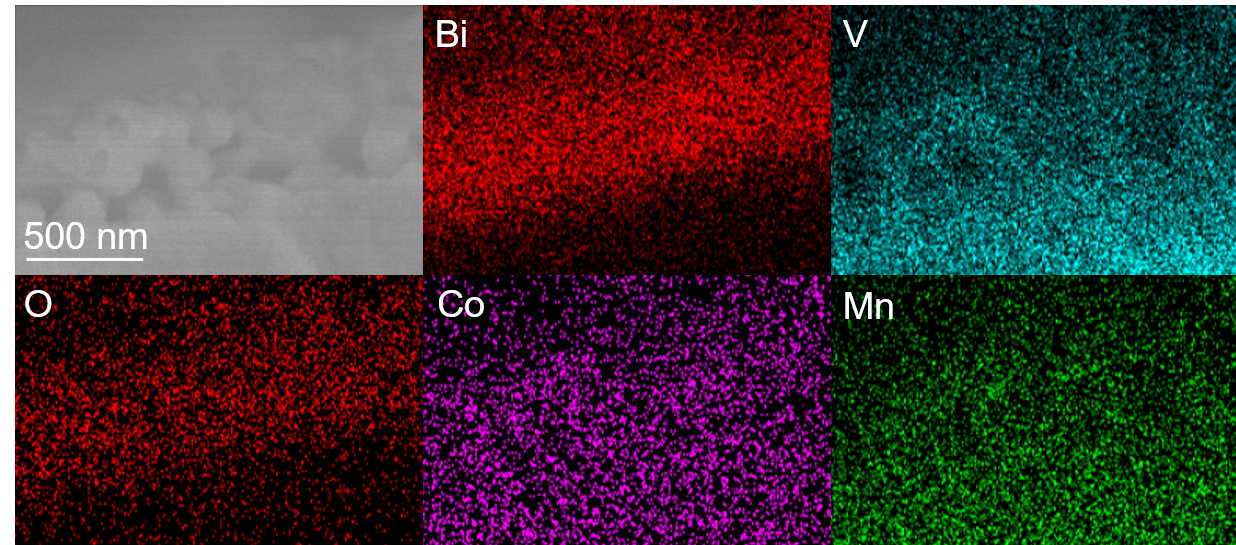


**Figure S6.** SEM-energy disperse spectroscopy (EDS) cross-sectional elemental mapping images of BV/CoO_x_/MnO_x_.

As presented in Figure S6, we can clearly see that the elements of Bi, V, O, Co, and Mn are uniformly distributed on the surface of BV.


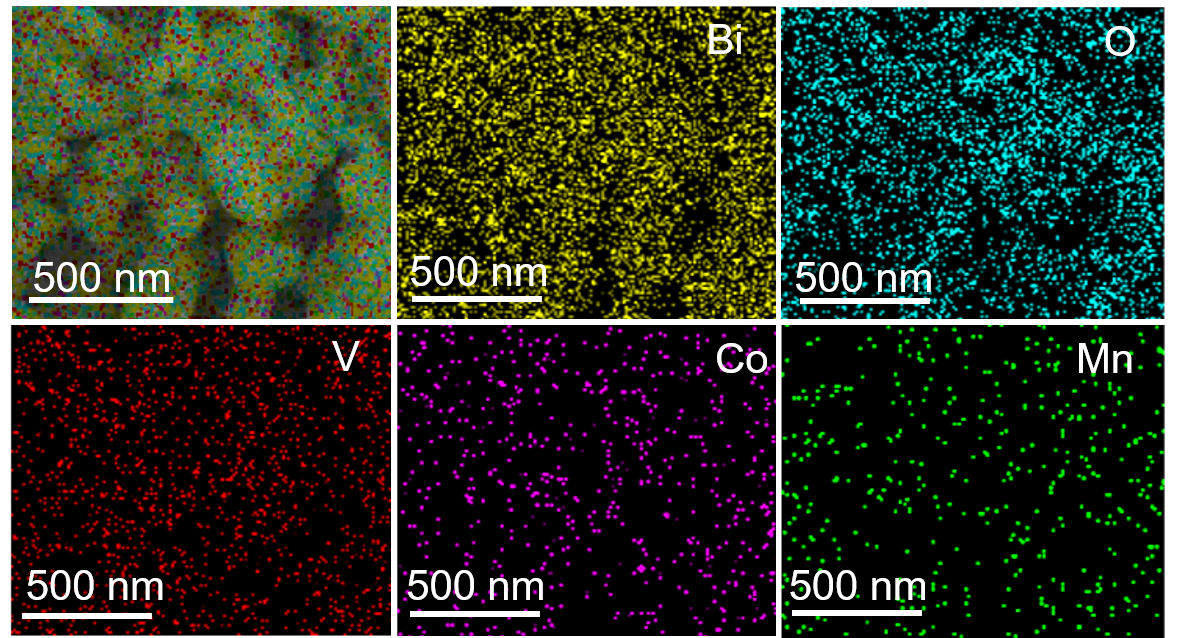


**Figure S7.** SEM-EDS elemental mapping images of BV/CoO_x_/MnO_x_.

EDS mapping further confirms the formation of heterointerface of CoO_x_/MnO_x_.


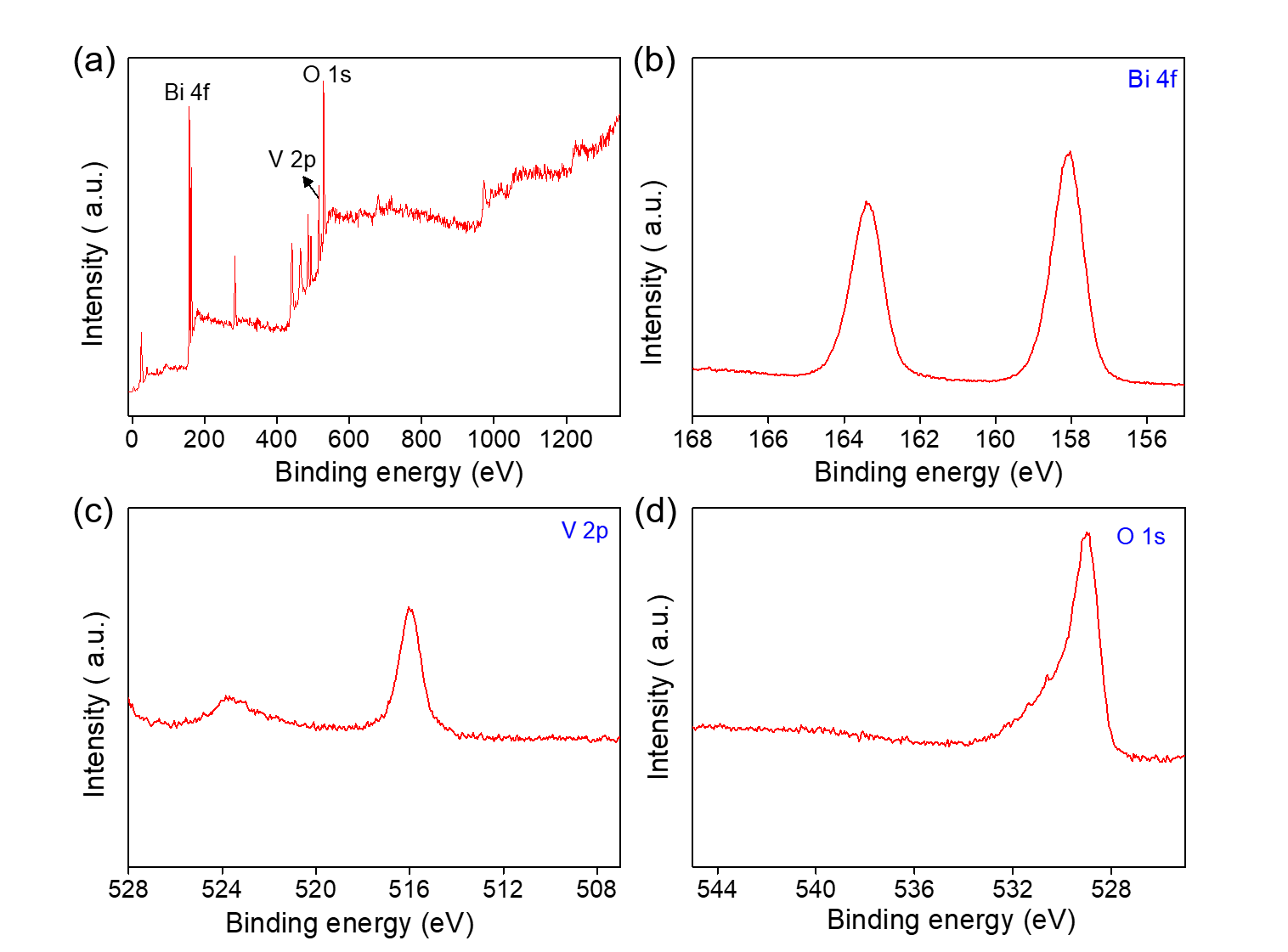


**Figure S8.** (a) X-ray photoelectron spectroscopy (XPS) survey spectra (b-d) XPS spectra of Bi 4f, V 2p, and O 1s for BV.

Figure S8 displays the characteristic peaks of Bi 4f (b), V 2p (c), and O 1s (d) for BV, which align with earlier report.^[9]^


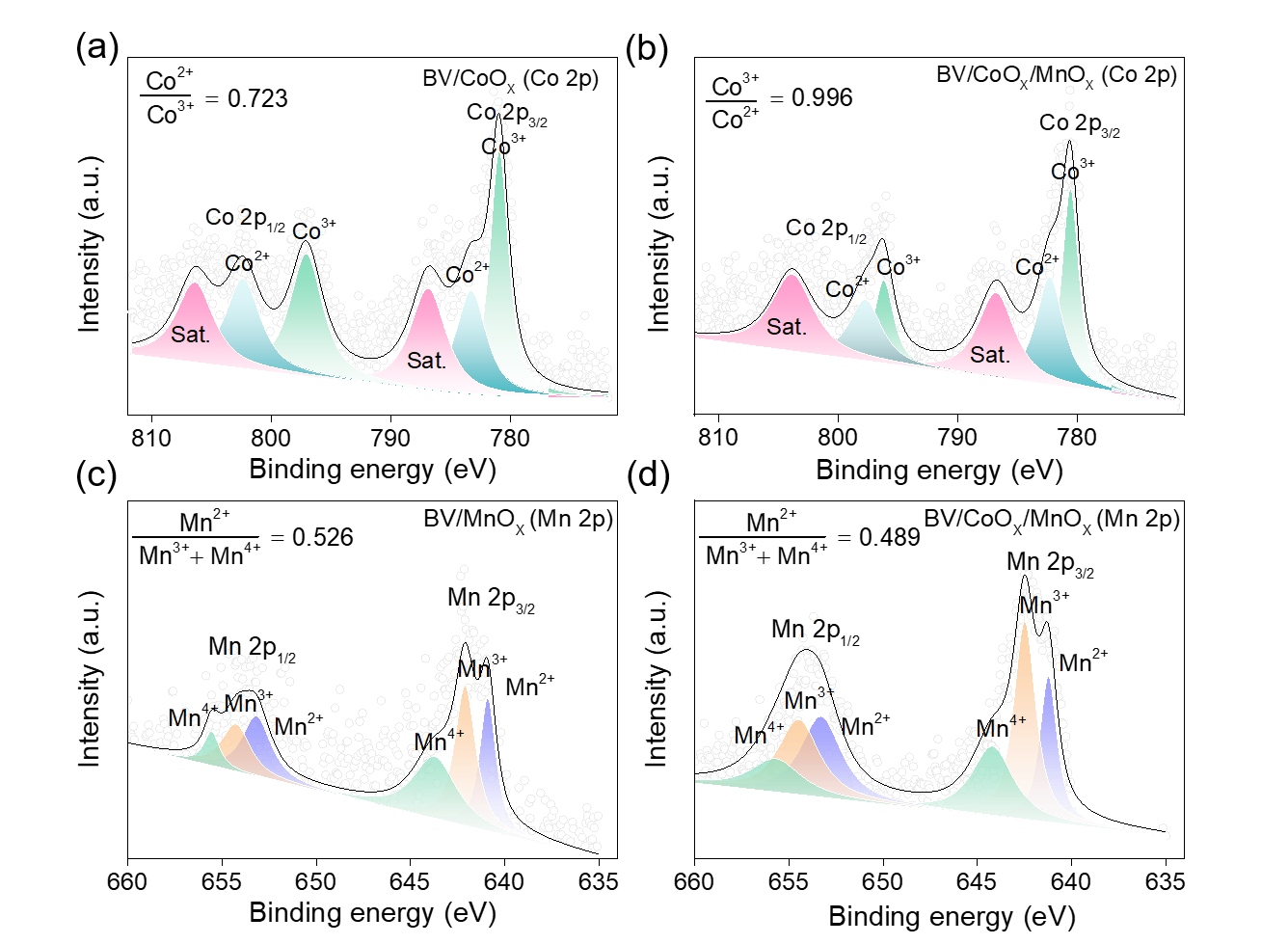


**Figure S9.** (a,b) XPS spectra of Co 2p for BV/CoO_x_ and BV/CoO_x_/MnO_x_ samples. (c,d) XPS spectra of Mn 2p for BV/MnO_x_ and BV/CoO_x_/MnO_x_ photoanodes.


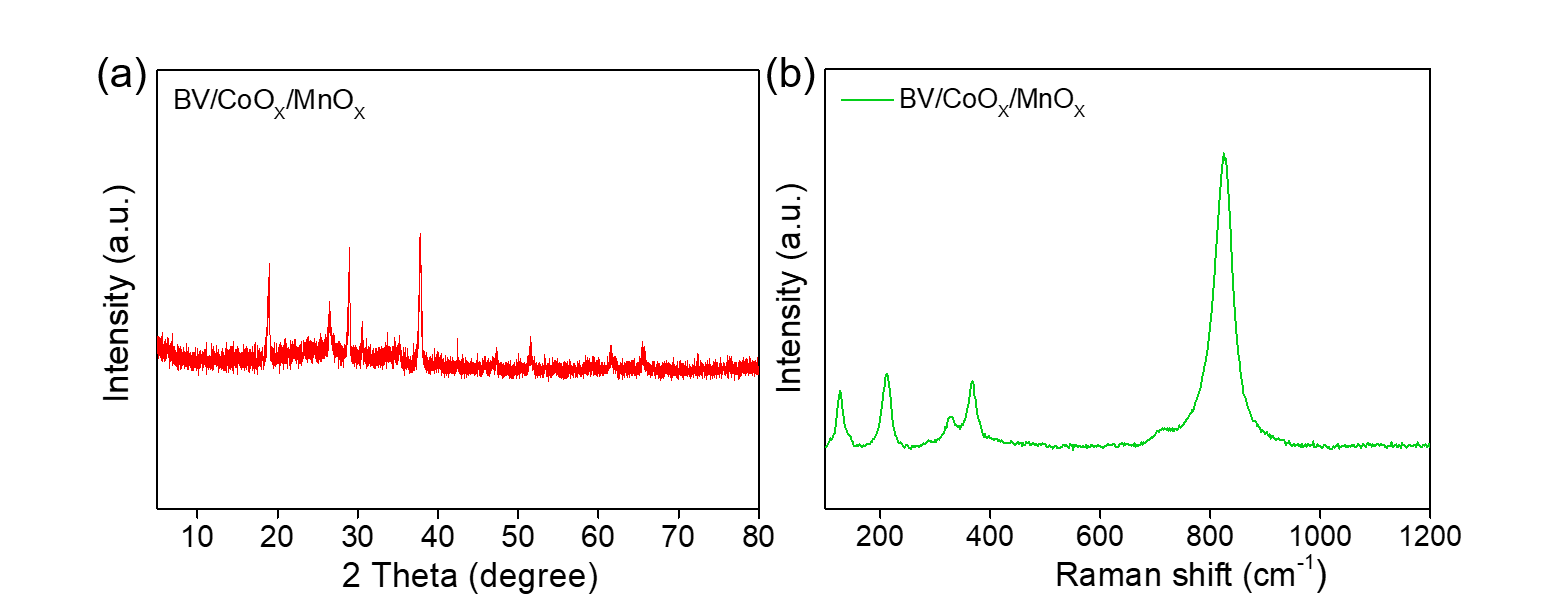


**Figure S10.** (a) XRD patterns and (b) Raman spectra of BV/CoO_x_/MnO_x_.

As described in Figure S10, no distinct characteristic peaks for CoO_x_/MnO_x_ can be observed by XRD spectra and Raman analysis, indicating that the CoO_x_/MnO_x_ is uniformly distributed on the surface of BV.


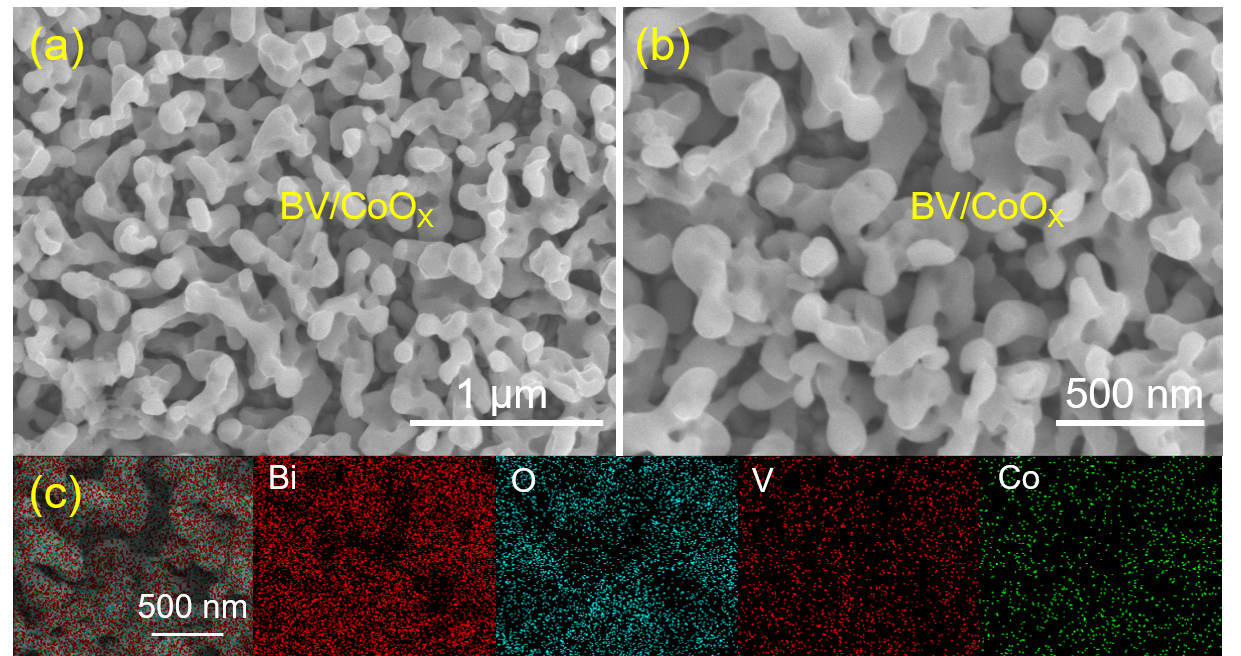


**Figure S11.** (a,b) SEM images of BV/CoO_x_. (c) SEM-EDS elemental mapping images of BV/CoO_x_.

The SEM images demonstrate that the BV/CoO_x_ system has been successfully prepared by comparing the surface roughness before and after the deposition of BV. SEM-EDS confirms that CoO_x_ is uniformly distributed on the surface of BV (Figure S11).


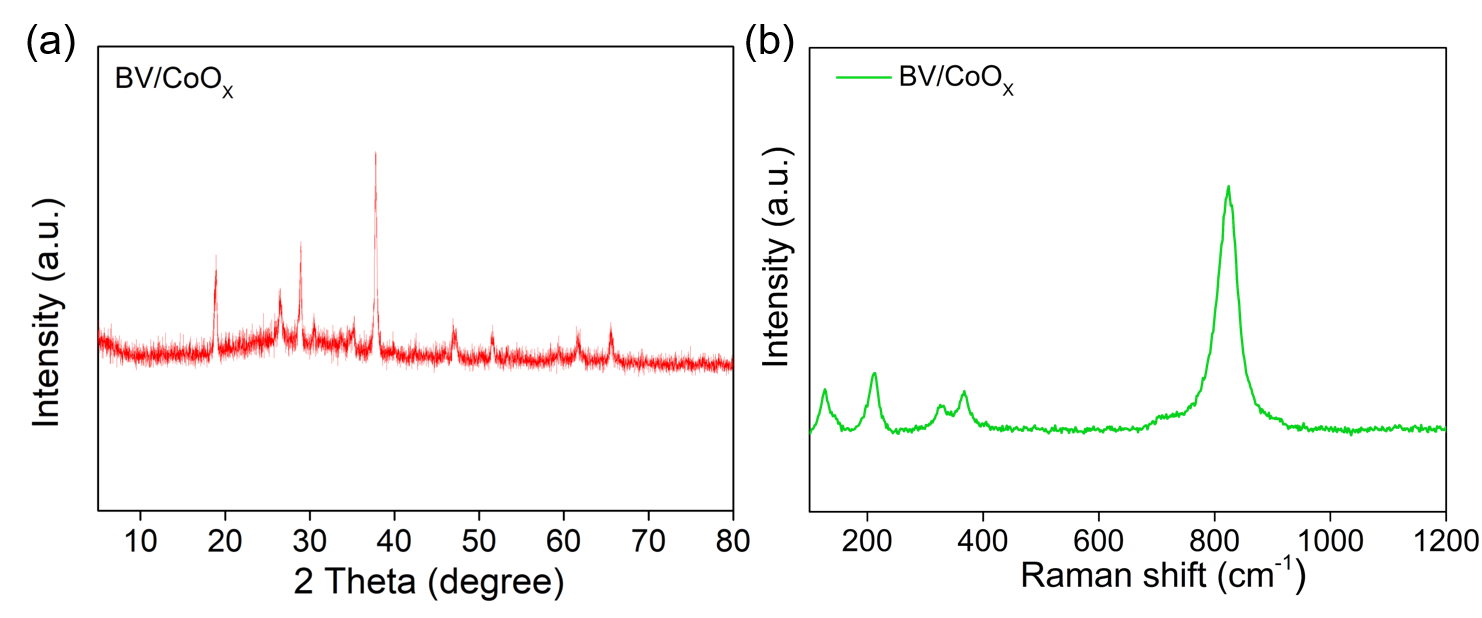


**Figure S12.** (a) XRD patterns and (b) Raman spectra of BV/CoO_x_.

In Figure S12, XRD spectra and Raman analysis show no distinct characteristic peaks for CoO_x_, indicating that the ultrathin CoO_x_ is uniformly distributed on the surface of BV.


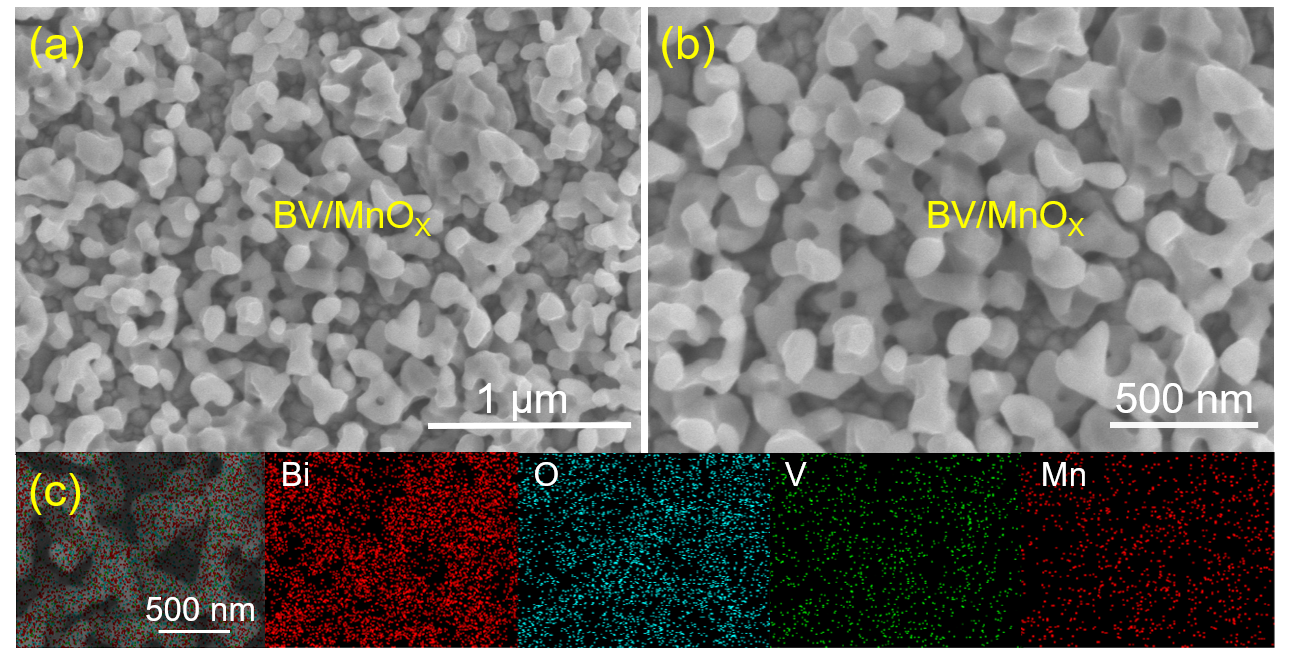


**Figure S13.** (a,b) SEM images of BV/MnO_x_. (c) SEM-EDS elemental mapping images of BV/MnO_x_.

The SEM images clearly demonstrate that the BV/MnO_x_ system has been successfully formed by comparing the surface roughness before and after the deposition of BV. SEM-EDS confirms that MnO_x_ is uniformly distributed on the surface of BV (Figure S13).


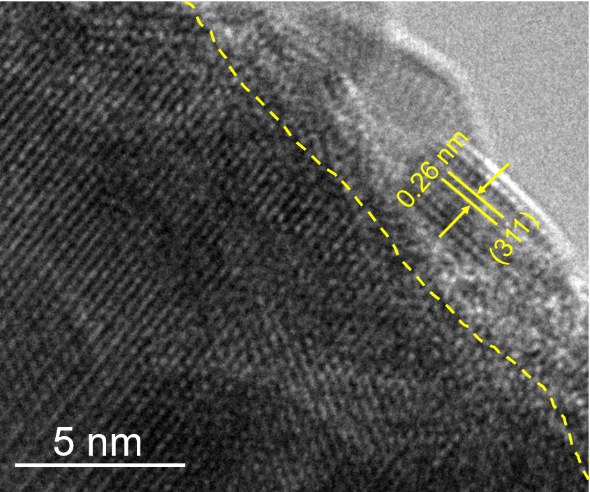


**Figure S14.** HR-TEM image of BV/MnO_x_.

In Figure S14, the lattice fringe with a spacing of 0.26 nm corresponds to the spacing of (311) planes of MnO_x_.^[10]^


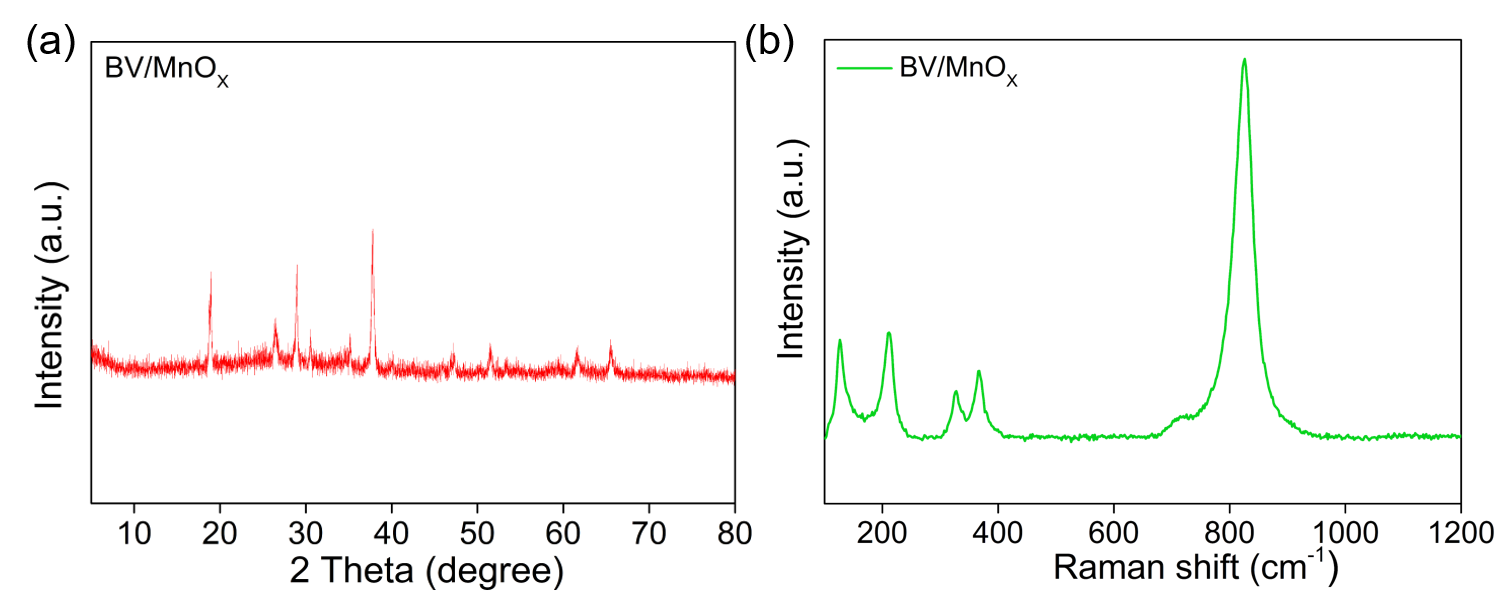


**Figure S15.** (a) XRD patterns and (b) Raman spectra of BV/MnO_x_.

XRD spectra and Raman spectroscopy do not reveal any distinct characteristic peaks for MnO_x_ (see Figure S15), indicating that the ultrathin MnO_x_ is evenly distributed on the surface of BV.


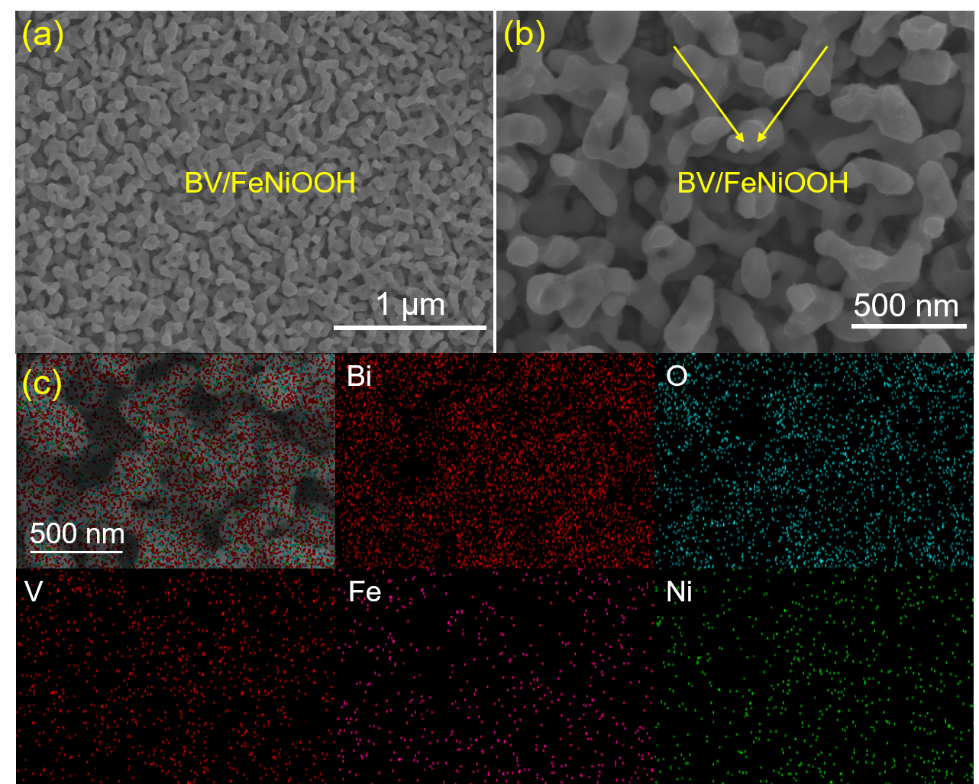


**Figure S16.** (a,b) SEM images of BV/FeNiOOH. (c) SEM-EDS elemental mapping images of BV/FeNiOOH.

SEM-EDS confirms that FeNiOOH is uniformly distributed on the surface of BV (Figure S16).


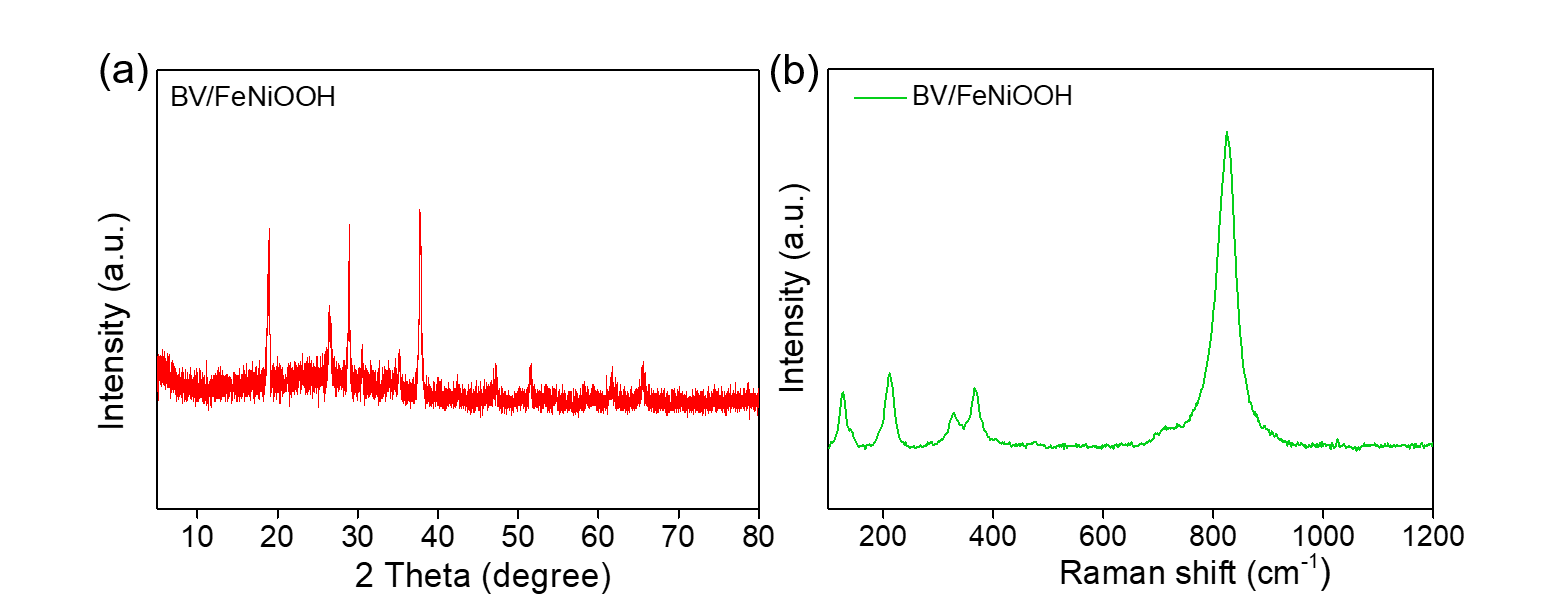


**Figure S17.** (a) XRD patterns and (b) Raman spectra of BV/FeNiOOH.

Similarly, the XRD spectra and Raman spectroscopy do not display any specific characteristic peaks for FeNiOOH (refer to Figure S17), suggesting that the ultrathin FeNiOOH is uniformly distributed on the surface of BV.


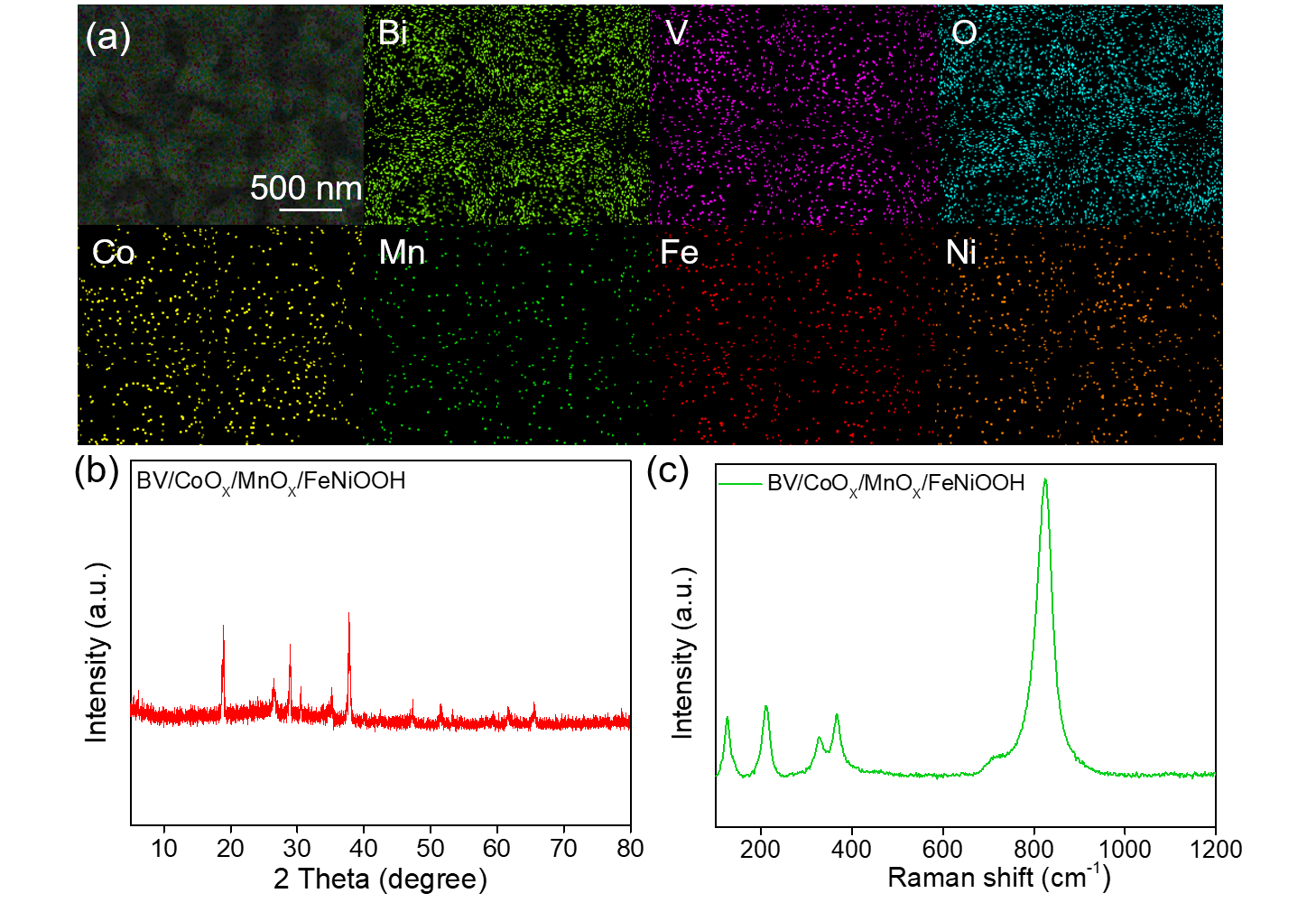


**Figure S18.** (a) SEM-EDS elemental mapping images, (b) XRD patterns and (c) Raman spectra of BV/CoO_x_/MnO_x_/FeNiOOH.

As shown in Figure S18b, the XRD pattern of the BV/CoO_x_/MnO_x_/FeNiOOH photoanodes do not exhibit any signals from CoO_x_/MnO_x_ or FeNiOOH, likely due to the minimal loading amount, the extremely thin layer, and the uniform distribution, as corroborated by the result of Raman in Figure S18c.





**Figure S19.** The spectra comparison of our solar simulator with the standard AM 1.5 G.

As illustrated in Figure S19, the spectra obtained from our solar simulator is closely correspond to the standard AM 1.5 G, which aligns with prior report.^[11]^


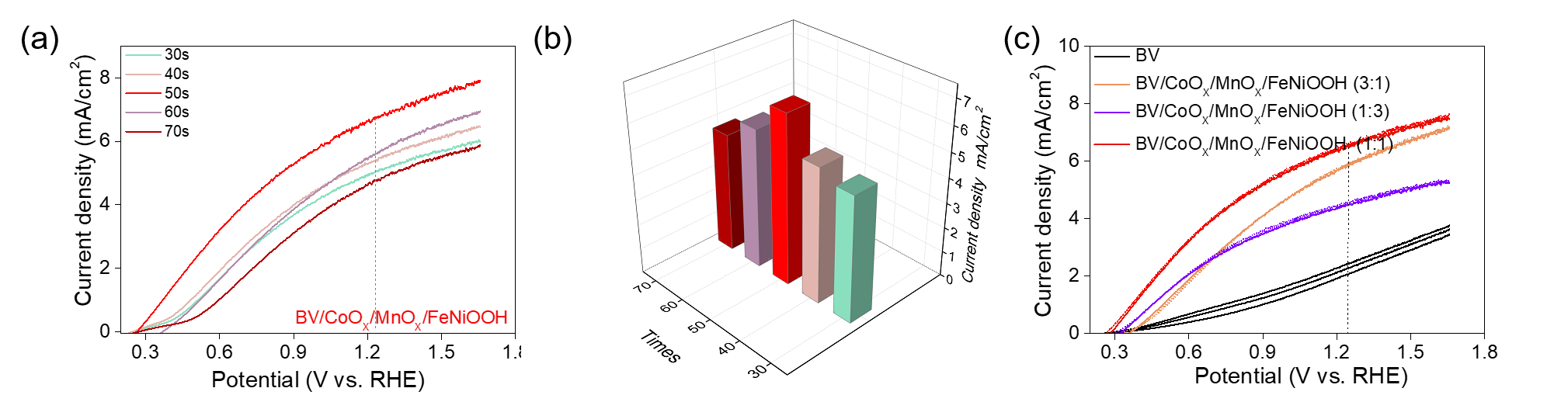


**Figure S20.** (a) Photocurrent densities of BV/CoO_x_/MnO_x_/FeNiOOH with different electrodeposition-times.(b) Photocurrent density values at different deposition times at 1.23 V_RHE_. (c) Photocurrent density versus potential curves of different photoanodes.

As presented in Figure S20a,b, with the deposition time increasing, the photocurrent shows a trend of increasing first and then decreasing, which reflects the influence of different thicknesses. Figure S20c demonstrates the repeatability of various samples, and the photocurrent density for BV/CoO_x_/MnO_x_/FeNiOOH (1:1, 50s) can achieve up to 6.75±0.1 mA/cm^2^ at 1.23 V_RHE_.


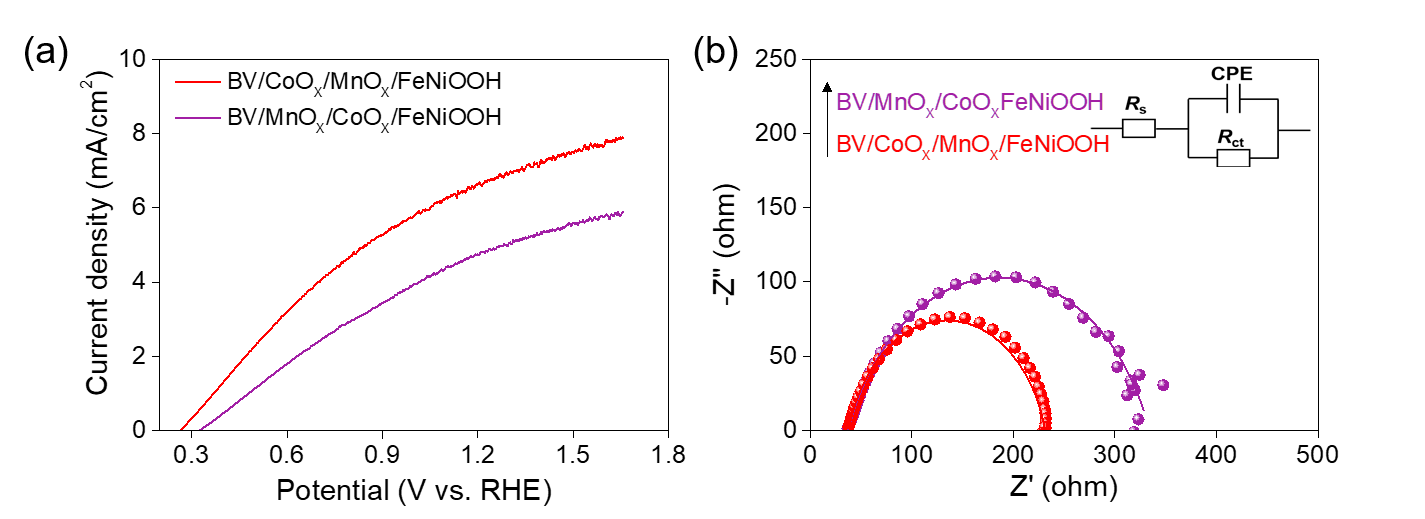


**Figure S21.** (a) Linear-sweep voltametric (LSV) curves. (b) EIS for different photoanodes.

When the order of CoO_x_/MnO_x_ is changed to MnO_x_/CoO_x,_ a negative result is confirmed by the results of LSV (Figure S21a) and EIS (Figure S21b).


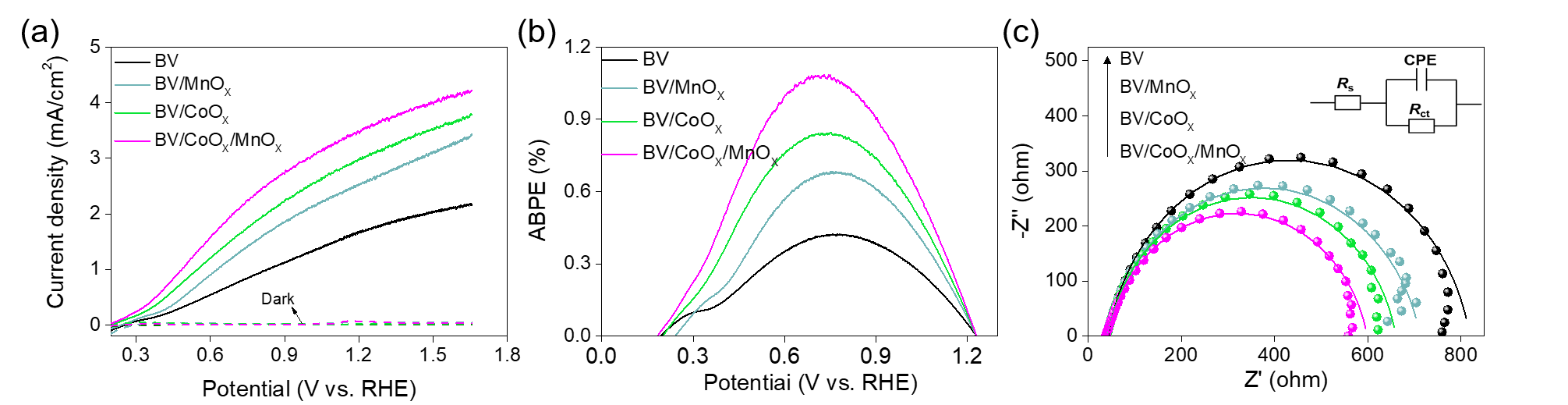


**Figure S22.** (a) LSV curves. (b) ABPE results. (c) EIS for different photoanodes.

According to the LSV data illustrated in Figure S22a, the highest ABPE achieved by the BV/CoO_x_/MnO_x_ photoanodes was 1.08% at 0.73 V_RHE_, significantly surpassing the BV/CoO_x_ (0.84%), BV/MnO_x_ (0.68%), and pristine BV (0.42%), which can also be confirmed by the result of EIS (Figure S22c).


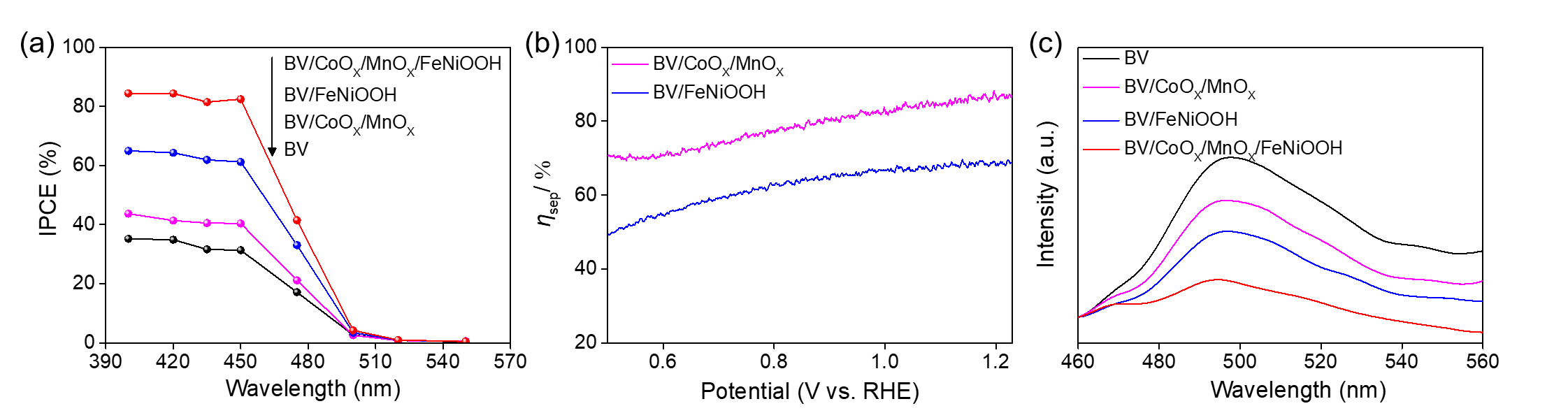


**Figure S23.** (a) Incident photo to current conversion efficiencies (IPCE) of different photoanodes s at 1.23 V vs. RHE. (b) Surface charge separation efficiency (*η*_sep_). (c) Photoluminescence (PL) spectra of different samples.


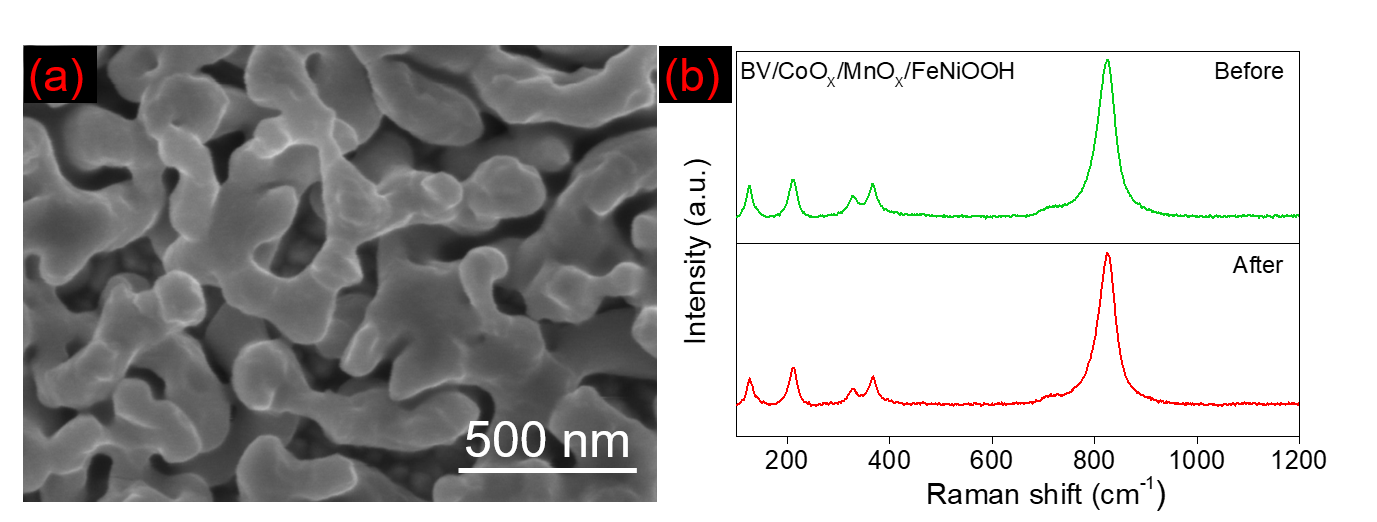


**Figure S24.** (a) SEM image and (b) Raman spectra of BV/CoO_x_/MnO_x_/FeNiOOH after PEC stability tests, respectively.

XRD and Raman results reveal that the structure of the BV/CoO_x_/MnO_x_/FeNiOOH photoanode is no apparent change after the PEC reaction (Figure S24), indicating its remarkable stability.


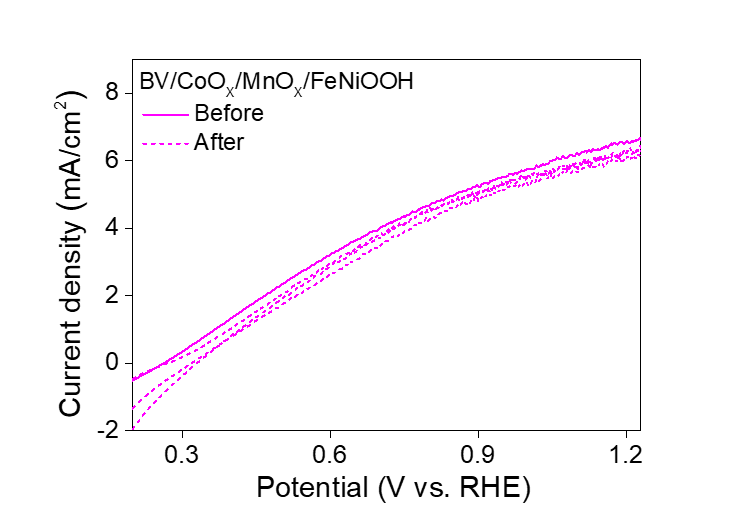


**Figure S25.** Photocurrent density of BV/CoO_x_/MnO_x_/FeNiOOH sample before and after stability.


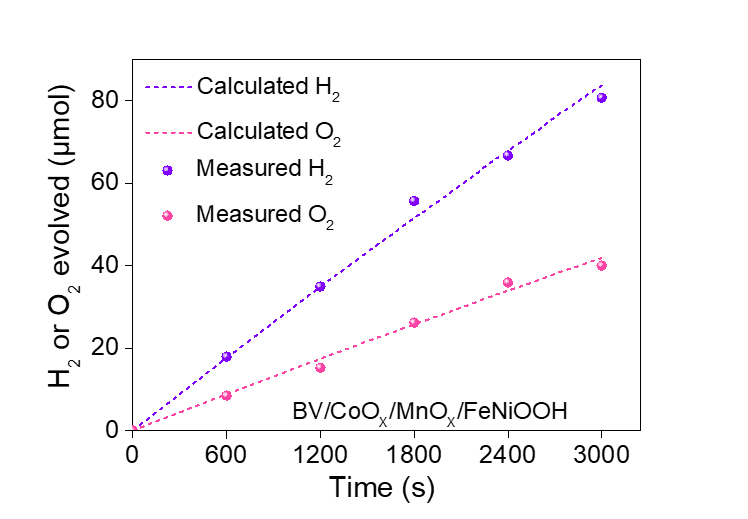


**Figure S26.** Plots of the theoretical charge number obtained from the stability curves collected at 1 V vs. RHE and the actual quantities of H_2_ and O_2_ evolution in K_3_BO_3_ electrolyte under AM 1.5 G illumination.

The quantities of H_2_ and O_2_ produced by BV/CoO_x_/MnO_x_/FeNiOOH were analyzed using an online gas chromatography (Figure S26).


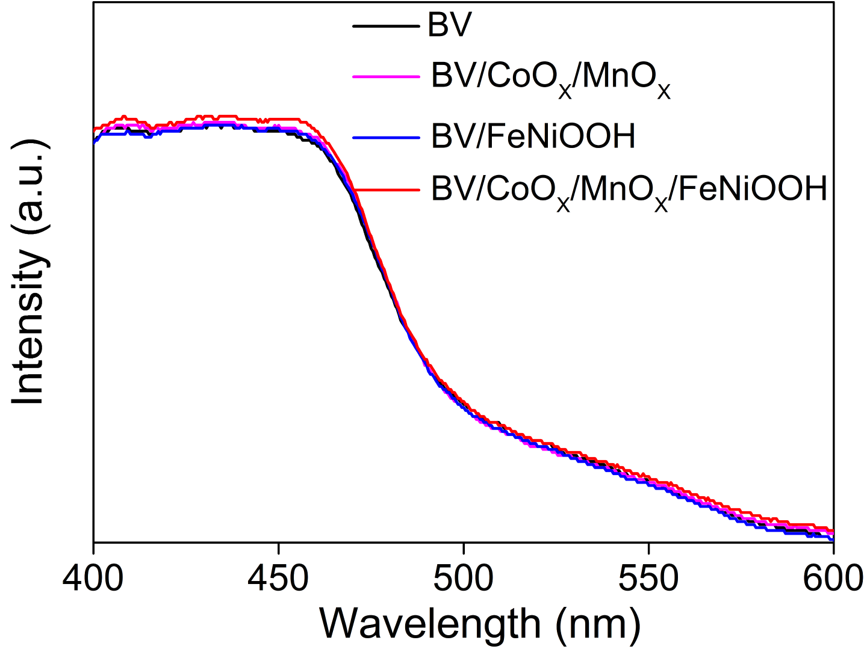


**Figure S27.** UV/Vis spectra of different samples.

The UV/vis absorption spectra (Figure S27) indicate that the addition of the CoO_x_/MnO_x_ and the FeNiOOH does not significantly affect the light absorption and PEC performance of BV-based photoanodes.


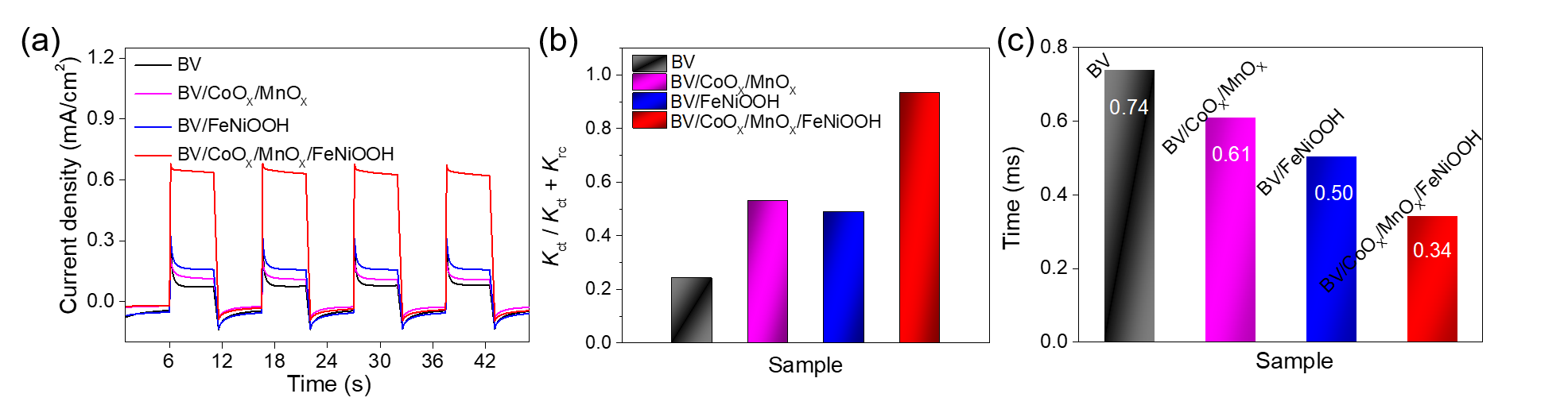


**Figure S28.** (a) Transient photocurrent curves of all photoanodes. (b) Charge transfer efficiency and (c) transit times of all samples.


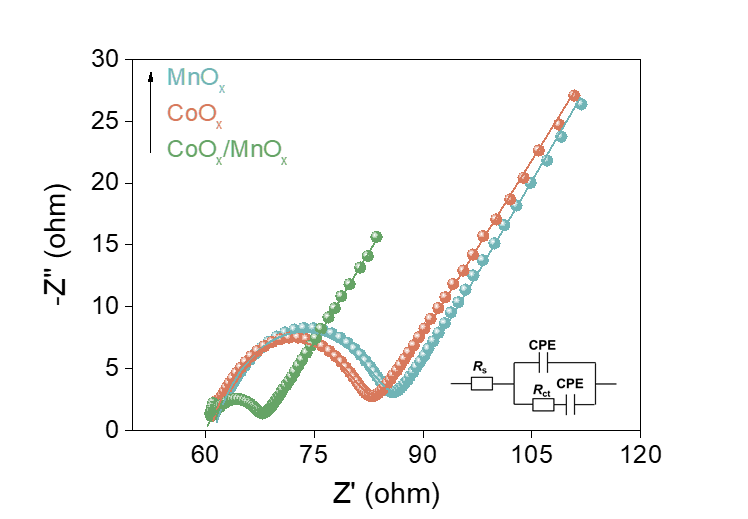


**Figure S29.** Electrochemical impedance spectroscopy of different photoanodes under dark (10mM K_3_[Fe(CN)]_6_/10mM K_4_[Fe(CN)]_6_ in 0.1M KCl).


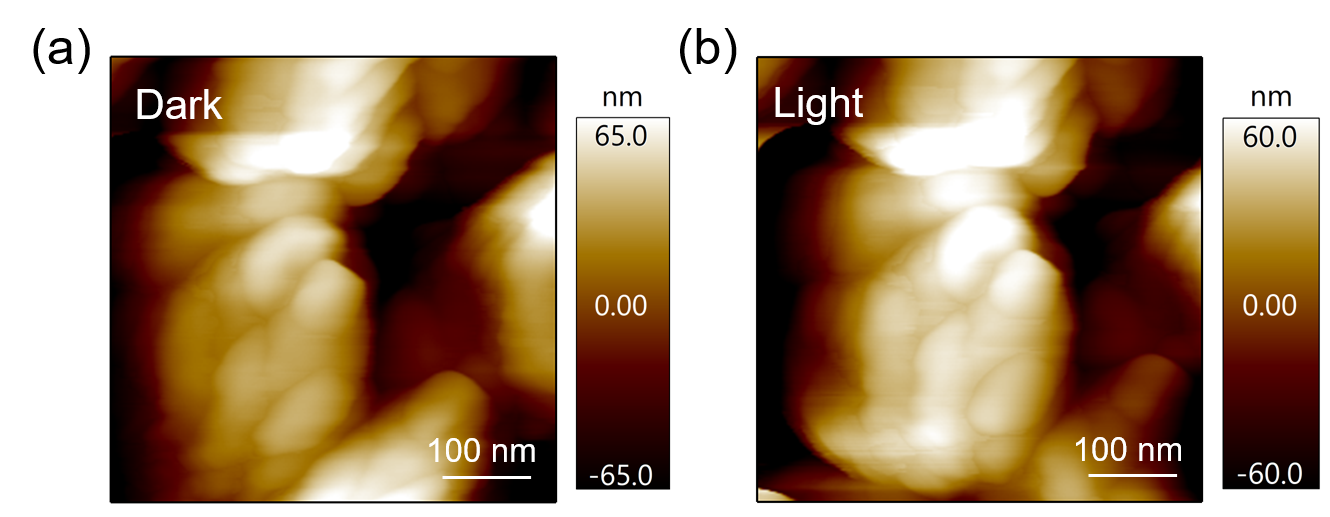


**Figure S30.** Kelvin probe force microscopy (KPFM) images of BV/CoO_x_/MnO_x_ samples on FTO substrates (a) in the dark and (b) under irradiation.


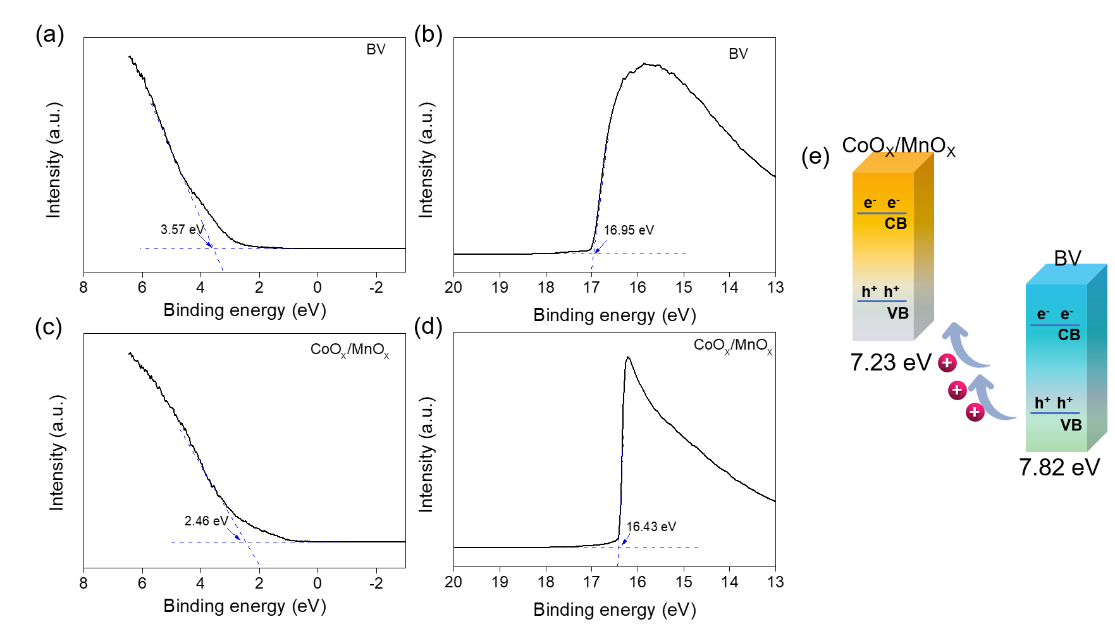


**Figure S31.** (a,b) UPS spectra of BV. (c,d) UPS spectra of CoO_x_/MnO_x_. (e) Energy band diagram of BV/CoO_x_/MnO_x_/FeNiOOH.

W_F_ = hν – (E_Cutoff_ – E_Fermi_)

Where, W_F_ is the work function; hν is the photon energy (21.2 eV); E_Cutoff_ is the binding energy of the secondary cutoff edge. E_Fermi_ is the binding energy of Fermi level.


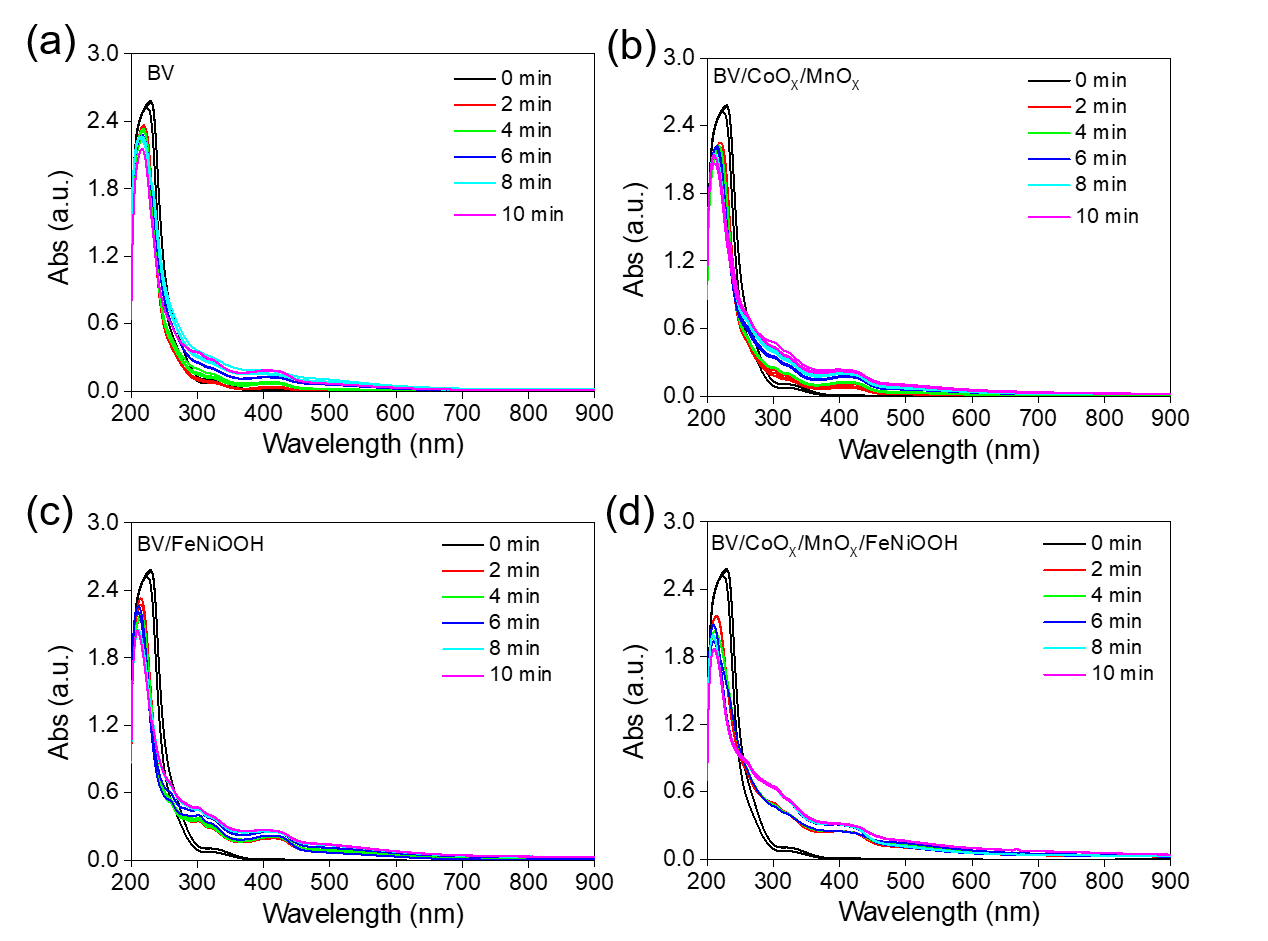


**Figure S32.** Time-dependent absorbance of electrolyte with BV-based photoanodes under irradiation.

As demonstrated in Figure S32, the UV/vis-SEC platform was utilized to examine the absorption of K_4_[Fe(CN)_6_]. Generally, the absorbance varies among different systems during the reactions. These findings indicate that the photogenerated holes can be readily transferred from the BV directly to the FeNiOOH surface via the CoO_x_/MnO_x_ heterointerface.


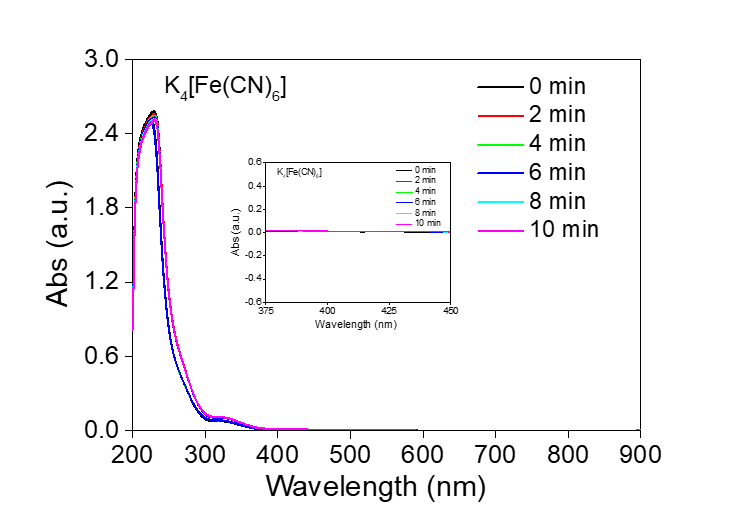


**Figure S33.** Time-dependent absorbance of K_4_[Fe(CN)_6_] without photoanodes.


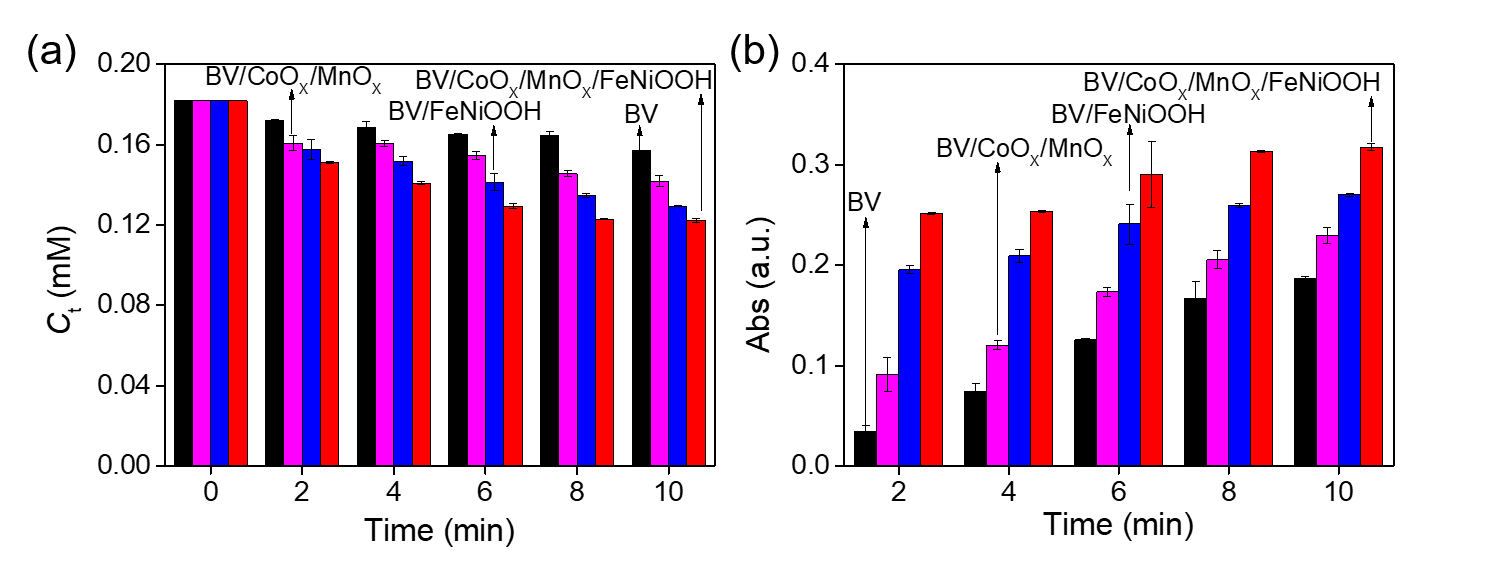


**Figure S34.** (a) Time-dependent concentration values (220 nm). (b) Time-dependent absorbance values (400 nm).


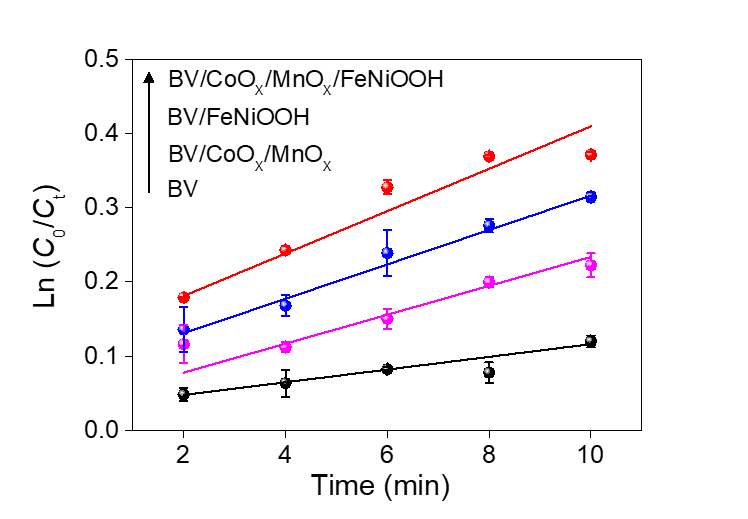


**Figure S35.** Time-dependent concentration of K_4_[Fe(CN)_6_] during oxidation process for different photoanodes.

From the analysis of hole transfer behavior, we can derive the corresponding hole transfer kinetics (denoted as *K*_h_) using a first-order kinetic model (Figure S35).^[12]^


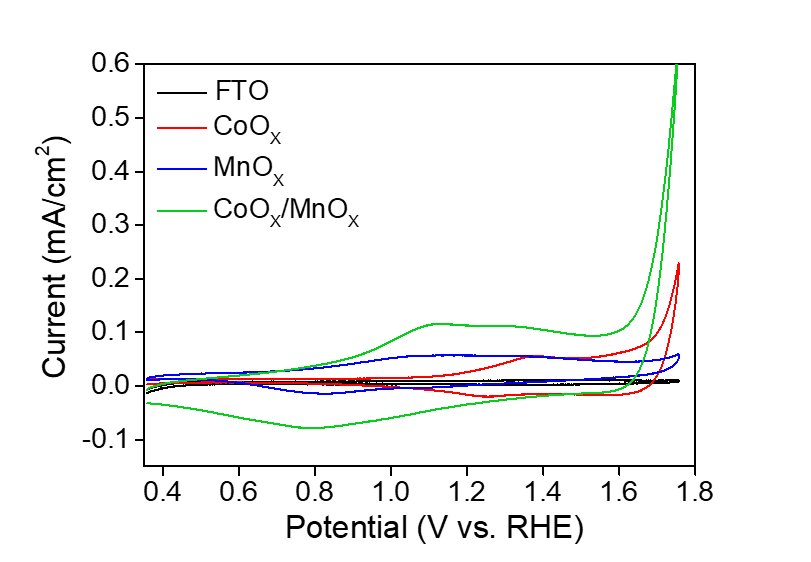


**Figure S36.** Cycle voltammetry curves of different samples.


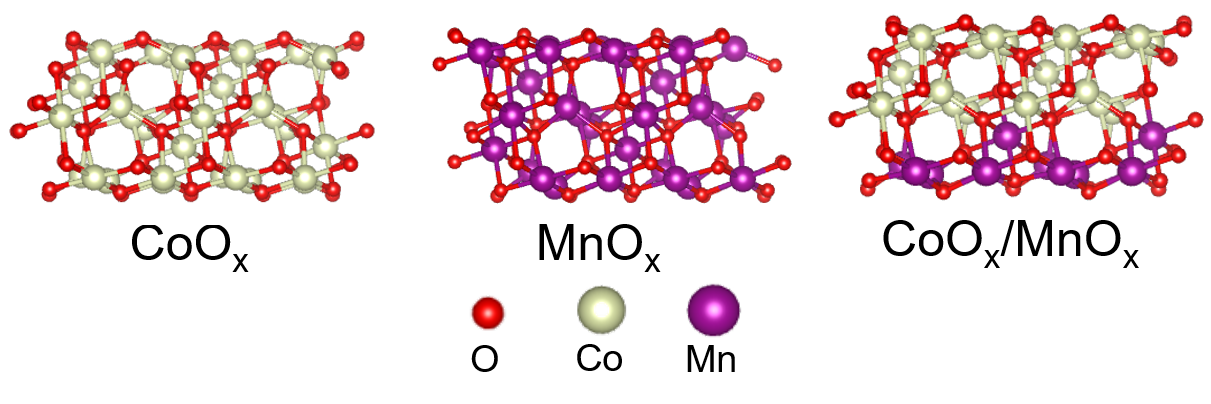


**Figure S37.** Optimized structure model of CoO_x_, MnO_x_, and CoO_x_/MnO_x_.


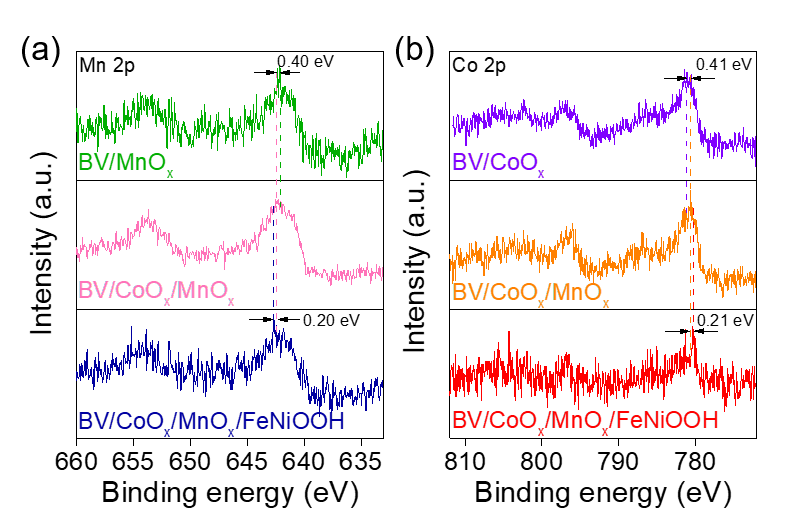


**Figure S38.** (a) XPS spectra of Co 2p for different samples. (b) XPS spectra of Mn 2p for different photoanodes.


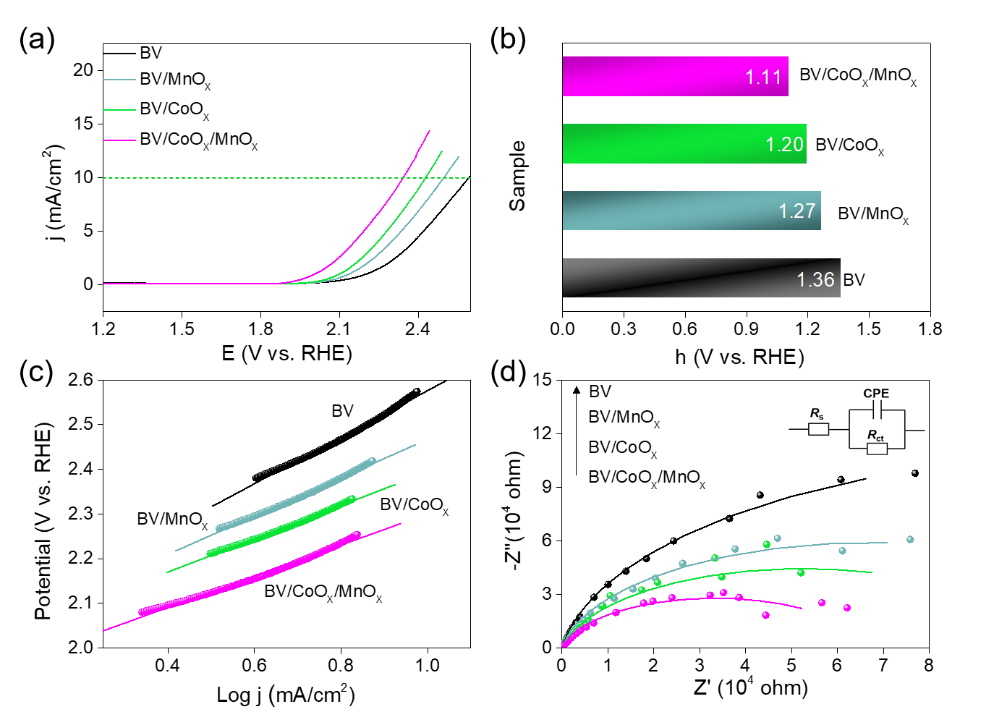


**Figure S39.** (a) LSV curves of different samples in dark. (b) The overpotentials of different samples at 10 mA/cm^2^. (c) Tafel slope results of different samples. (d) Electrochemical impedance spectroscopy of different samples under dark.


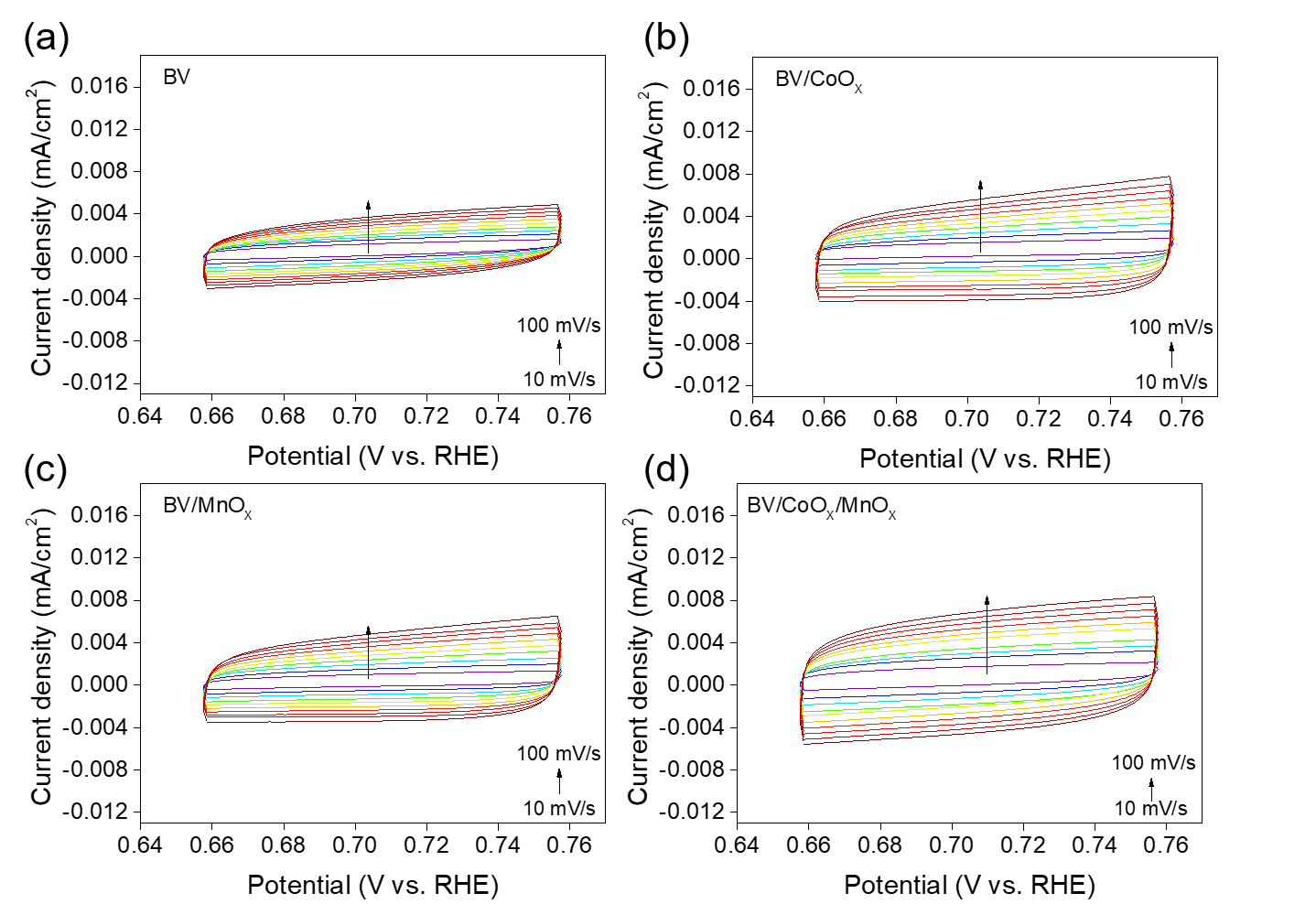


**Figure S40.** Voltammograms of different samples under dark. (c) Electrochemically active surface area (ECSA) evaluation.


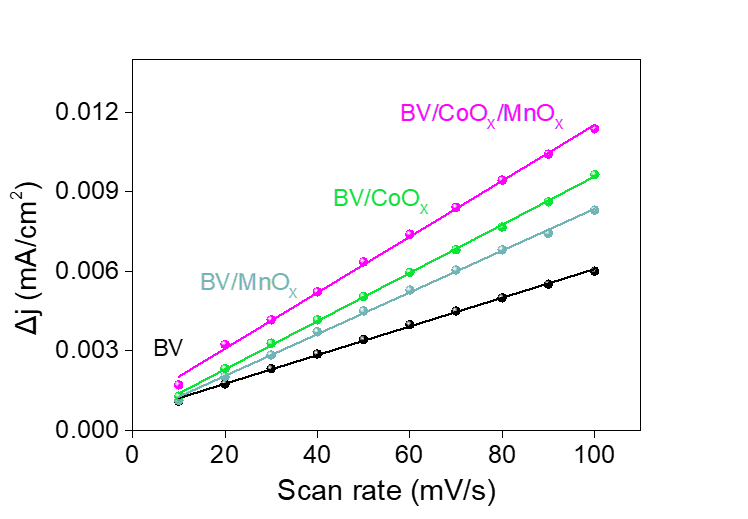


**Figure S41.** ECSA evaluation.


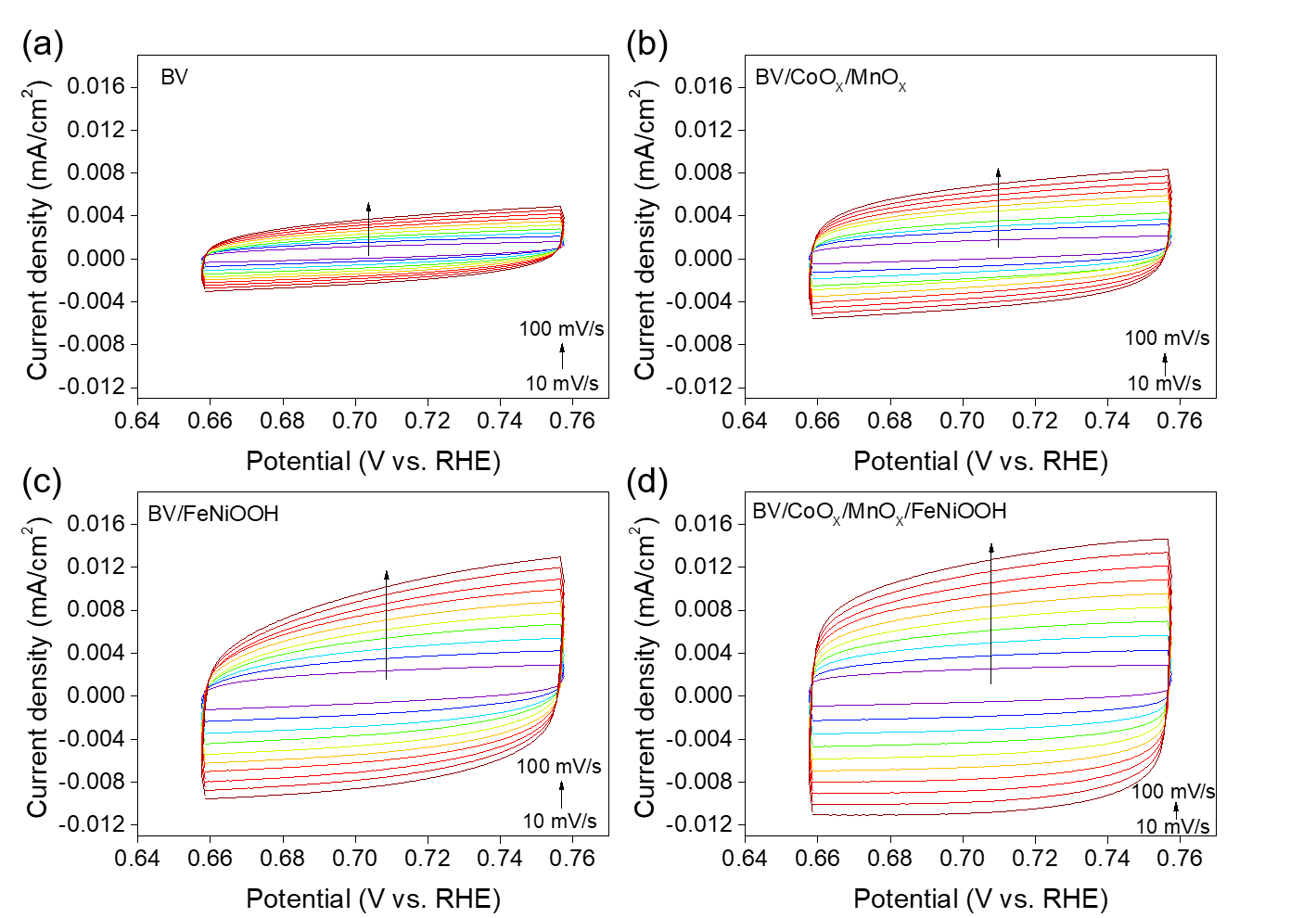


**Figure S42.** Voltammograms of different samples under dark.


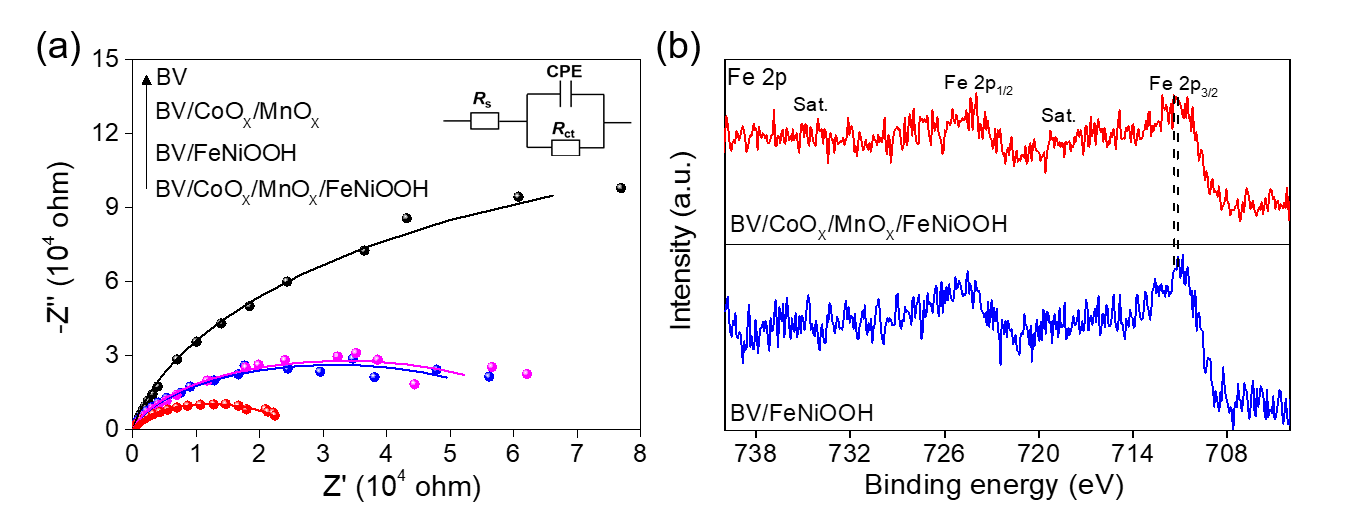


**Figure S43.** (a) Electrochemical impedance spectroscopy of different samples under dark. (b) XPS spectra of Fe 2p for different samples.

In Figure S43b, when comparing the Fe 2p results of the two samples, it can be observed that Fe 2p shows a significant shift towards higher binding energies. This indicates that the introduction of FeNiOOH induces an interfacial interaction, causing electron transfer.


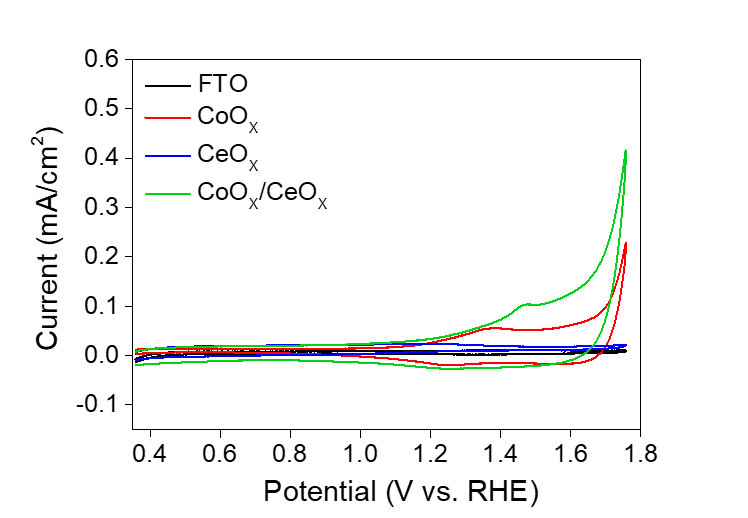


**Figure S44.** Cycle voltammetry curves of different samples.


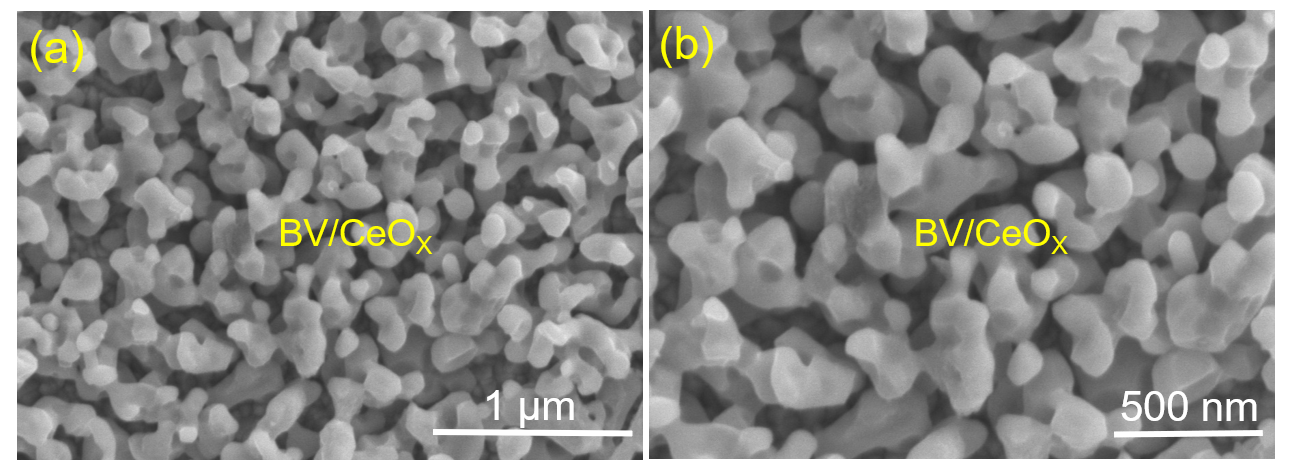


**Figure S45.** SEM images of BV/CeO_x_.


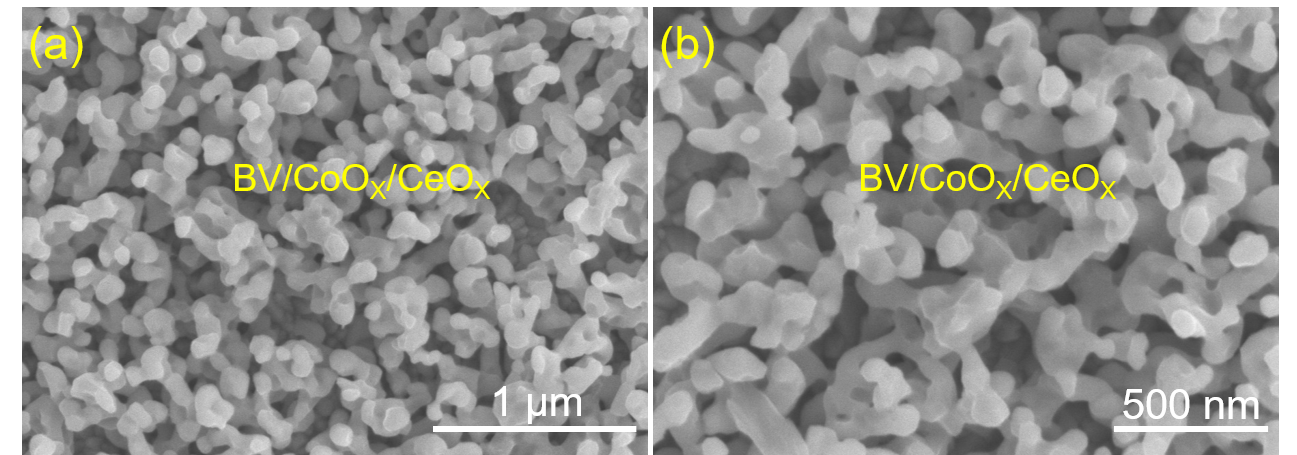


**Figure S46.** SEM images of BV/CoO_x_/CeO_x_.


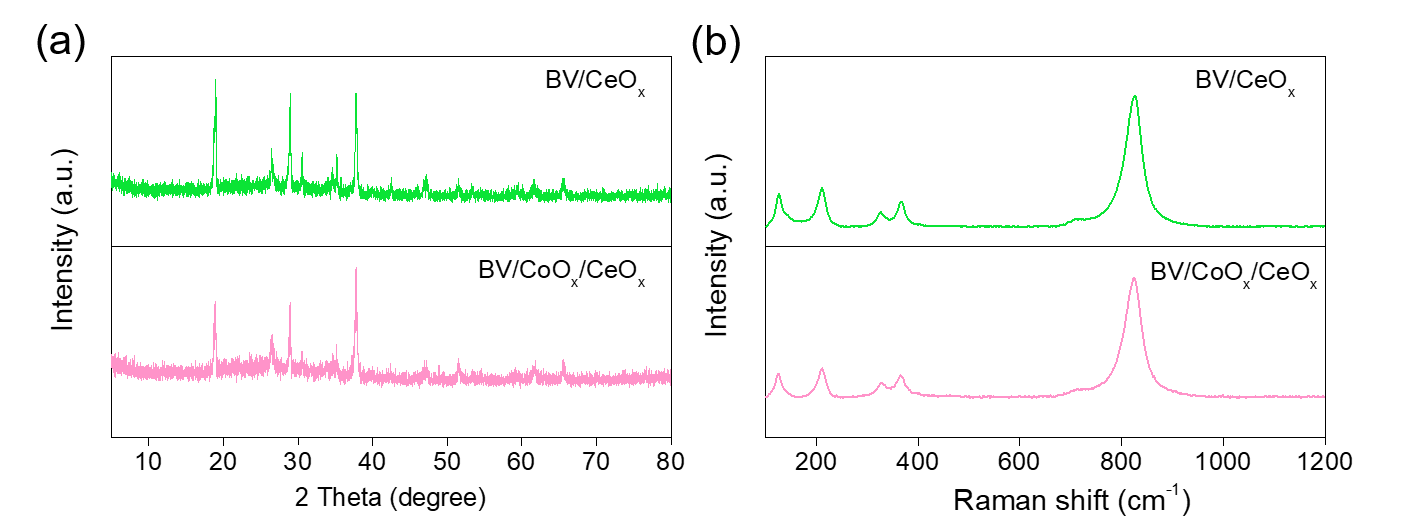


**Figure S47.** (a) XRD patterns and (b) Raman spectra of BV/CeO_x_ and BV/CoO_x_/CeO_x_.


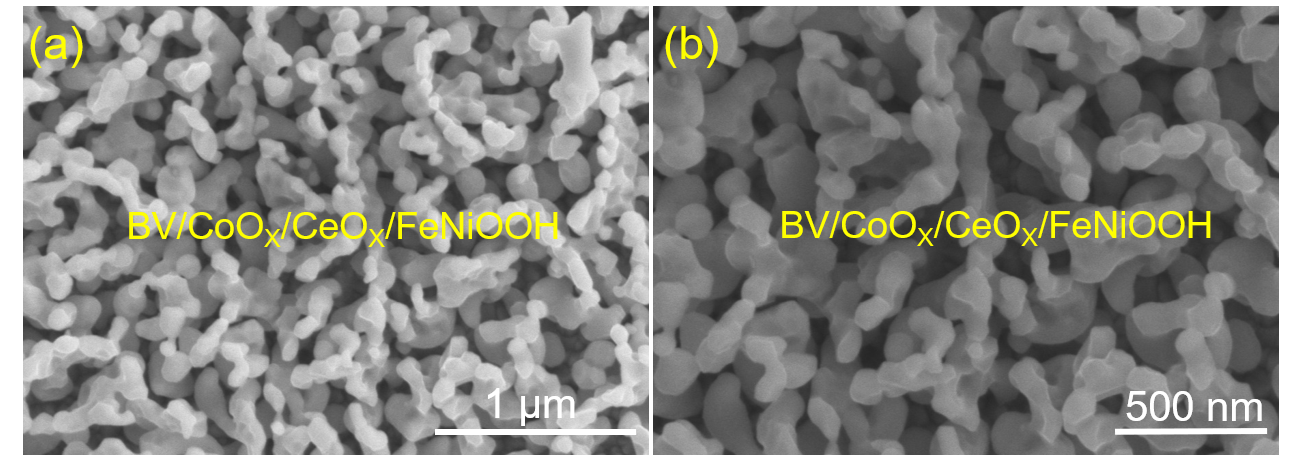


**Figure** **S48.** SEM images of BV/CoO_x_/CeO_x_/FeNiOOH.


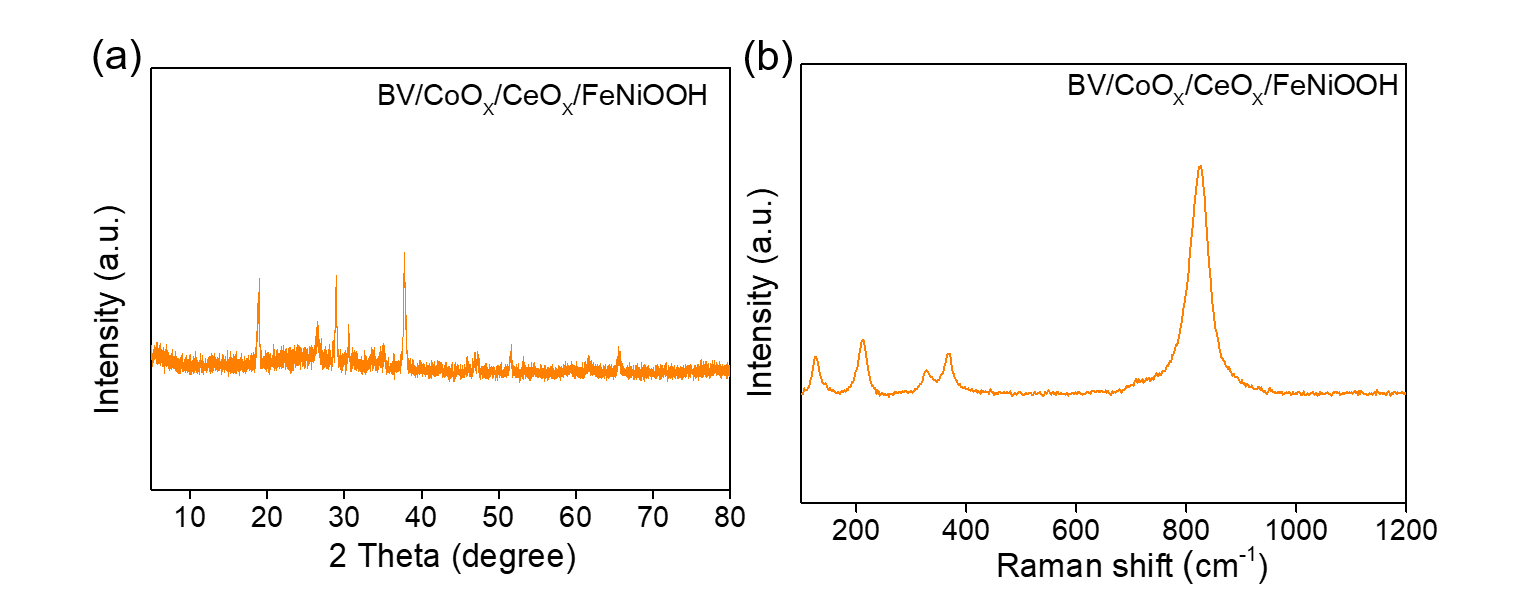


**Figure S49.** (a) XRD patterns and (b) Raman spectra of BV/CoO_x_/CeO_x_/FeNiOOH.

In contrast to the pure BV photoanode, no significant changes in the peaks were detected in the XRD and Raman spectra of BV/CoO_x_/CeO_x_/FeNiOOH (Figure S49), which can be attributed to their extremely thin thickness and uniform distribution.


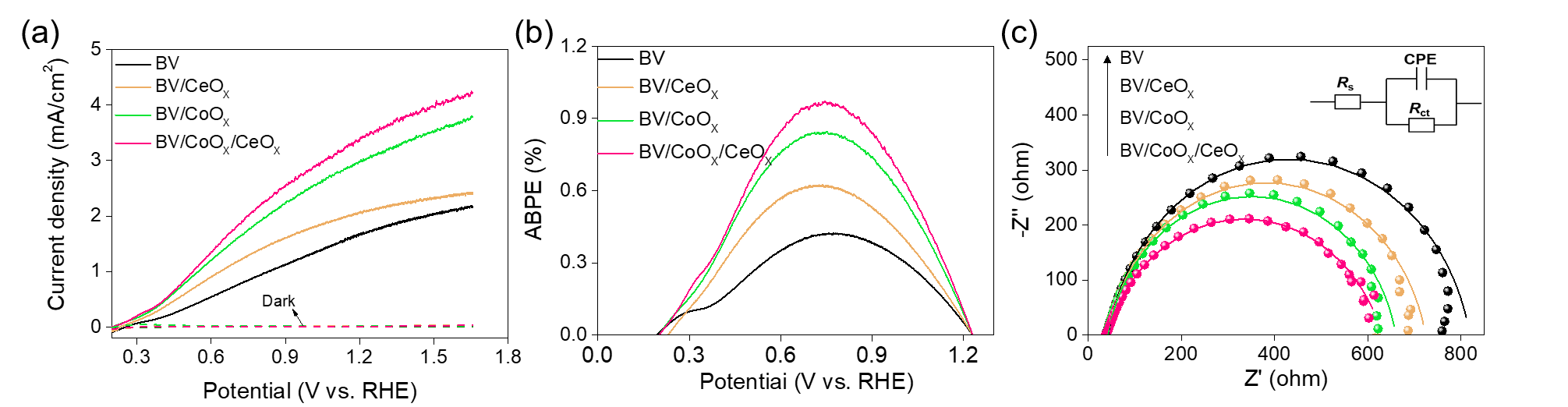


**Figure S50.** (a) LSV curves. (b) ABPE. (c) EIS for different photoanodes.

As presented in Figure S50, the photocurrent density obtained for the BV/CoO_x_/MnO_x_ photoanode is the highest among that of BV/CoO_x_, BV/CeO_x_, and pristine BV, which can also be confirmed by the result of ABPE and EIS. The BV/CoO_x_/CeO_x_ exhibits a lower charge-transfer resistance (*R*_ct_, 592 Ω) than that of BV (775 Ω), BV/CoO_x_ (618 Ω), and BV/CeO_x_ (682 Ω), indicating that the introduction of CoO_x_/CeO_x_ heterointerfaces enhances the charge transfer dynamics.


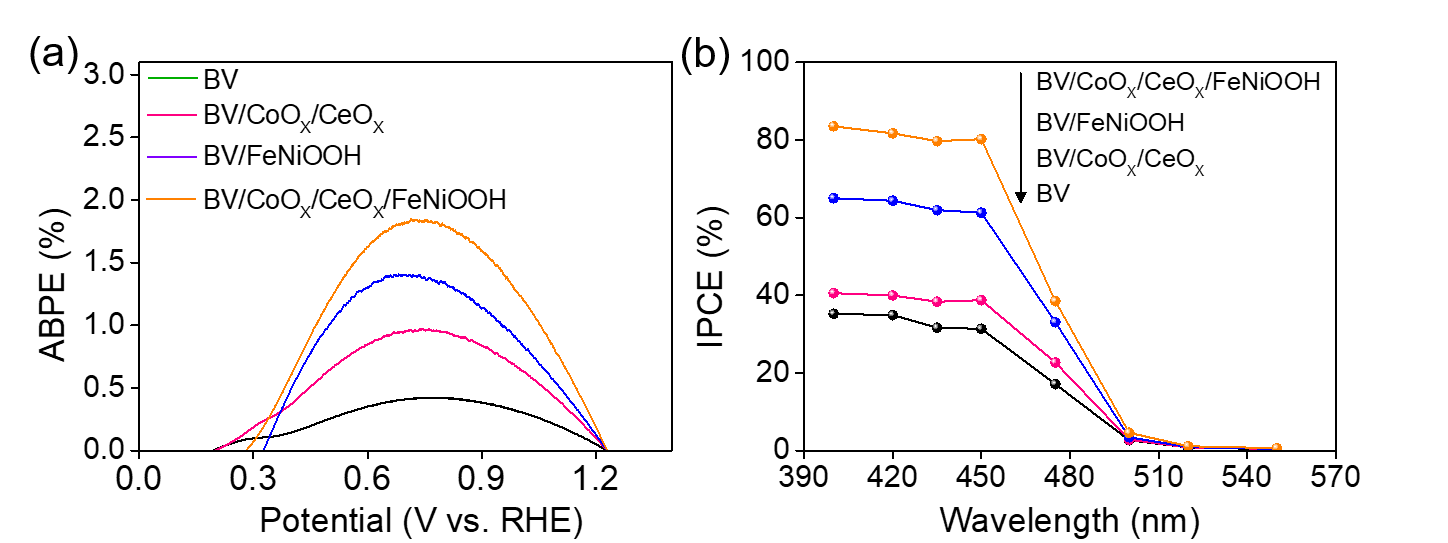


**Figure S51.** (a) ABPE and (b) IPCE of all photoanodes.

To clarify the impact of monochromatic light on the photocurrent density, the IPCE curves of the BV, BV/CoO_x_/CeO_x_, BV/FeNiOOH, and BV/CoO_x_/CeO_x_/FeNiOOH photoanodes were measured. As shown in Figure S51, the IPCE value of the BV/CoO_x_/CeO_x_/FeNiOOH photoanode is higher than those of the BV, BV/CoO_x_/CeO_x_, and BV/FeNiOOH, photoanodes.


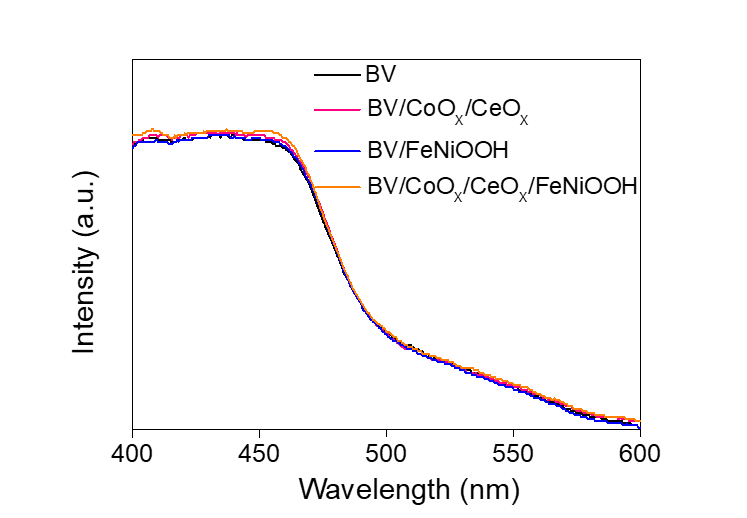


**Figure S52.** UV/Vis spectra of different samples.

The optical characters of photoanodes were evaluated by UV/Vis spectroscopy.As shown Figure S52, there is no obvious change upon light absorption, meaning that the influence of light absorption upon PEC activity by the incorporation of CoO_x_/CeO_x_ and FeNiOOH is almost negligible.


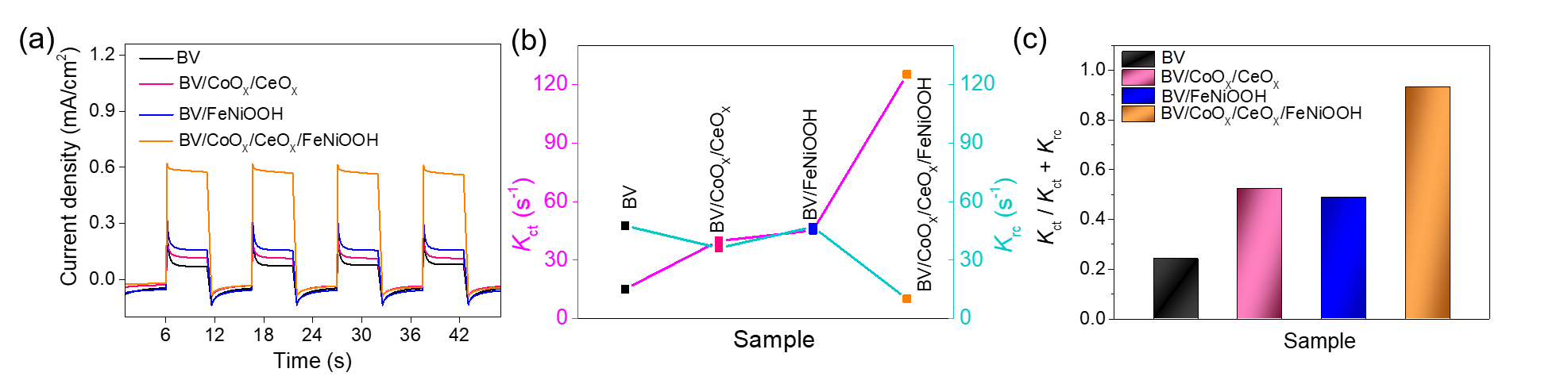


**Figure S53.** (a) Transient photocurrent curves of all photoanodes. (b) Charge transfer rate constant (*K*_ct_) and recombination rate constant (*K*_rc_) for different samples. (c) Charge transfer efficiency result.


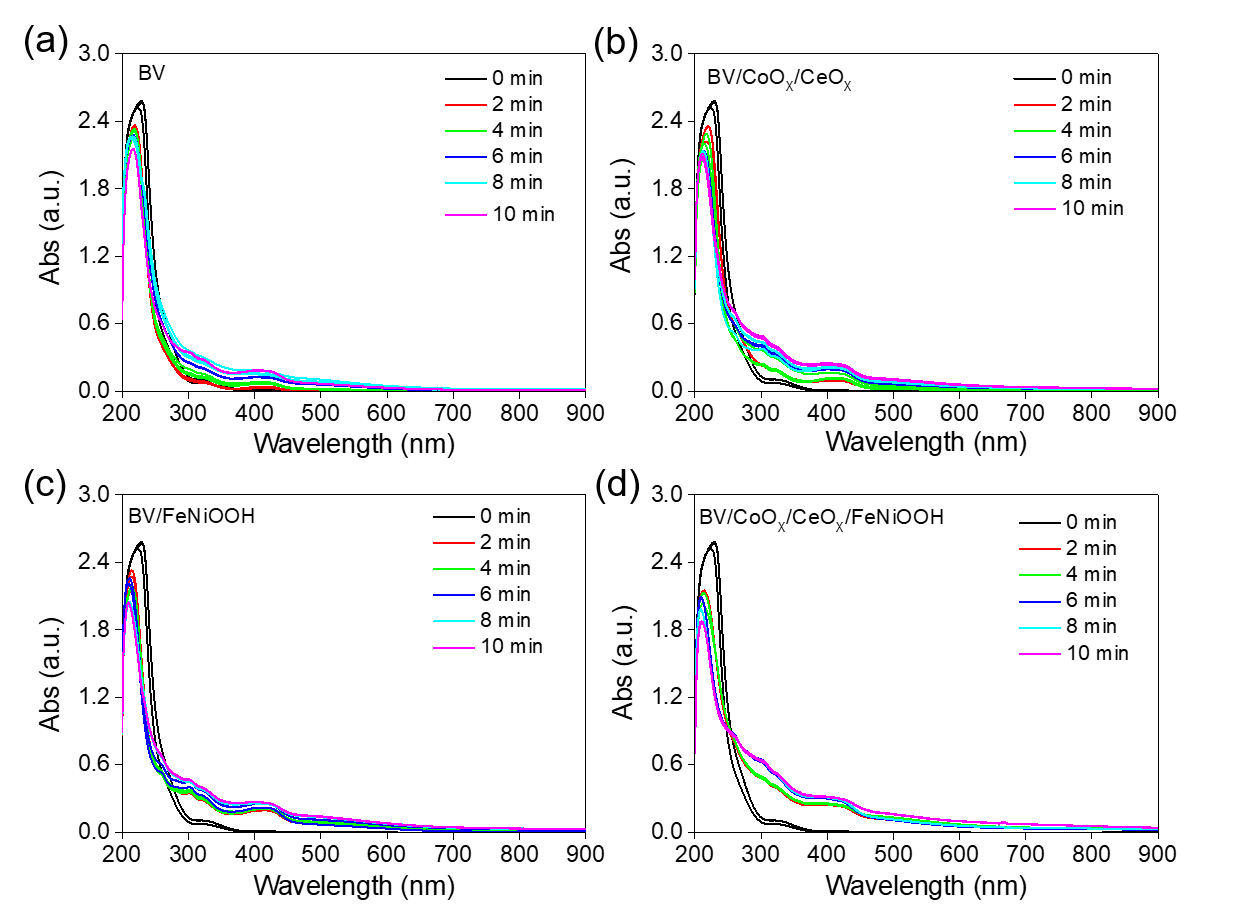


**Figure S54.** Time-dependent absorbance of electrolyte with BV-based photoanodes under irradiation.


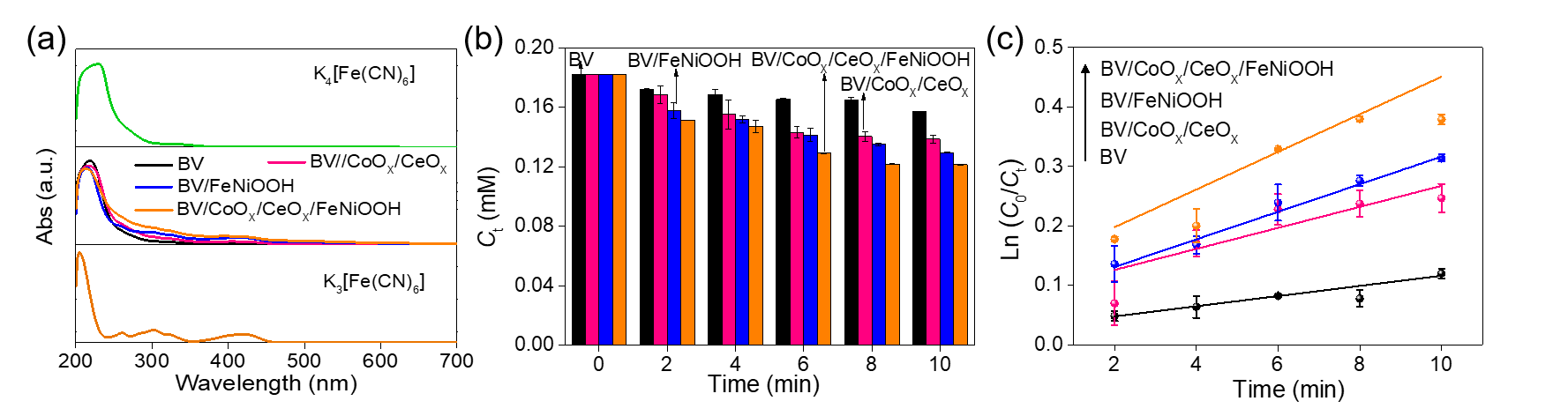


**Figure S55.** (a) Absorbance spectra for different samples. (b) Time-dependent concentration values (220 nm). (c) Potassium ferrocyanide oxidation during reactions.


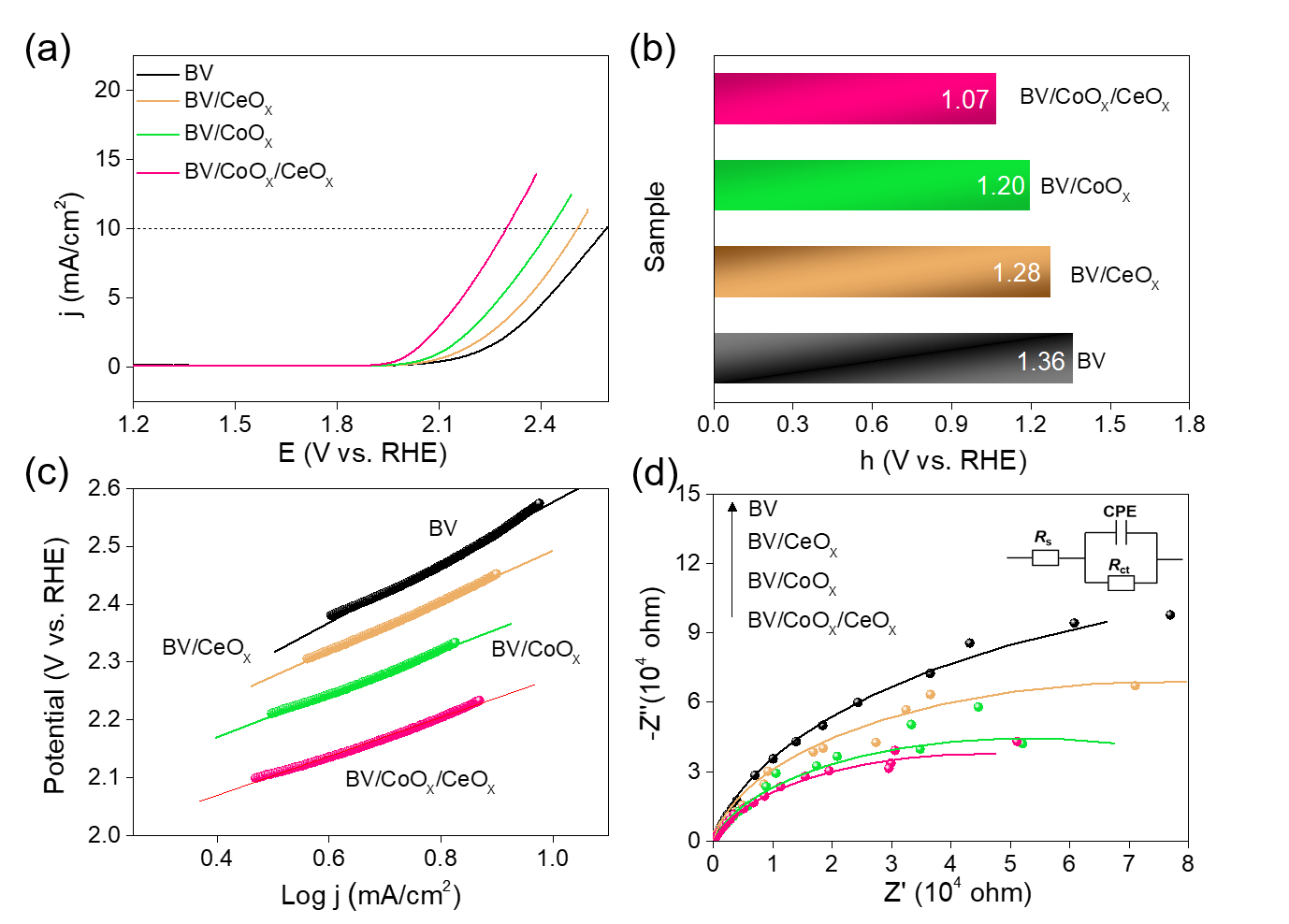


**Figure S56.** (a) LSV curves of different samples in dark. (b) The overpotentials of different samples at 10 mA/cm^2^. (b) Tafel slope results of different samples. (d) Electrochemical impedance spectroscopy of different samples under dark.


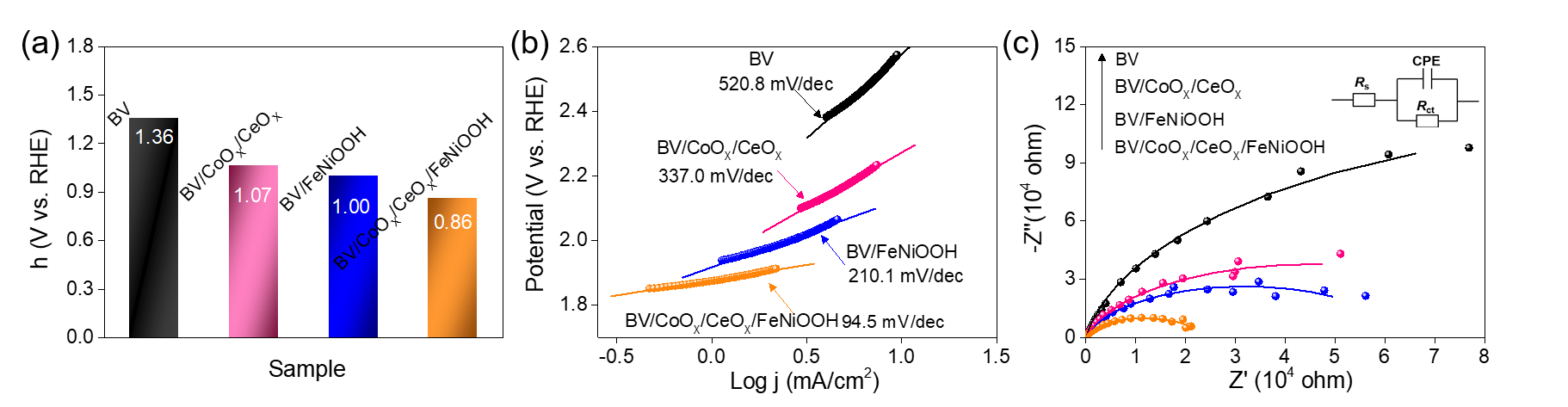


**Figure S57.** (a) The overpotentials of different samples at 10 mA/cm^2^. (b) Tafel slope results of different samples. (c) Electrochemical impedance spectroscopy of different samples under dark.


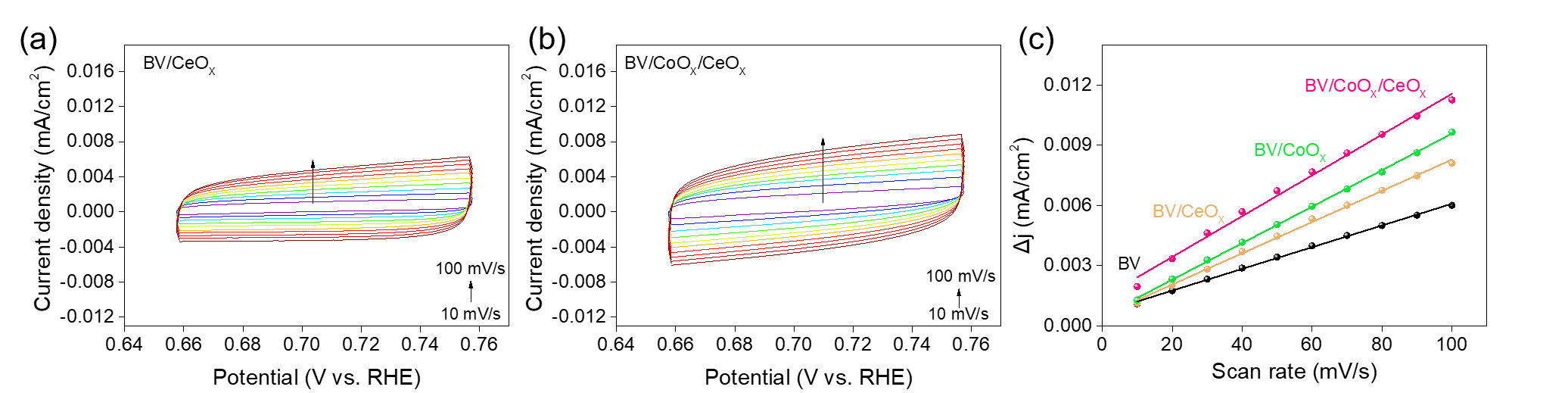


**Figure S58.** (a,b) Voltammograms of different samples under dark. (c) ECSA evaluation.


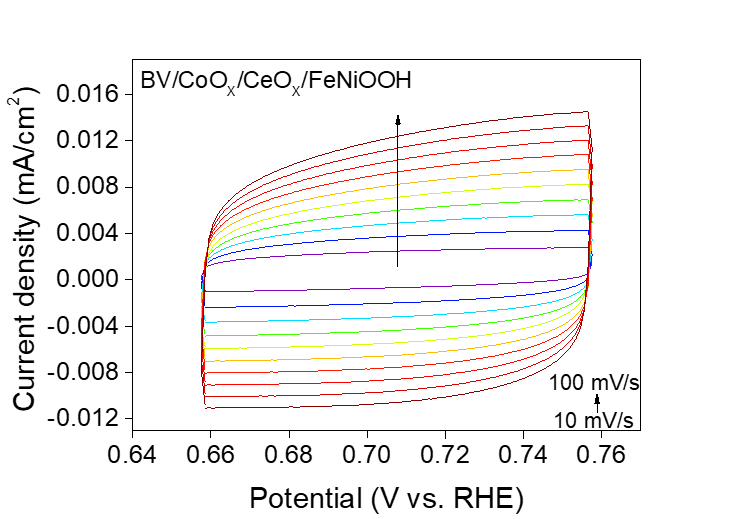


**Figure S59.** Voltammograms of BV/CoO_x_/CeO_x_/FeNiOOH samples under dark.

**Table S1.** Summary of recent significant progress of BV-based photoanodes.

| Photoanodes | Photocurrent at 1.23 V vs. RHE | Journal | Year | Ref |
| --- | --- | --- | --- | --- |
| BV/CoPi | 2.00 mA/cm^2^ | *Adv. Funct. Mater.* | 2016 | [13] |
| BV/Co-Bi | 3.20 mA/cm^2^ | *Angew. Chem. Int. Ed.* | 2017 | [14] |
| BV/CoFe-H | 2.48 mA/cm^2^ | *Adv. Funct. Mater.* | 2017 | [15] |
| BV/β-FeOOH | 4.30 mA/cm^2^ | *Angew. Chem. Int. Ed.* | 2018 | [16] |
| BV/LDH/pGO/CoPO_3_ | 4.45 mA/cm^2^ | *J. Am. Chem. Soc.* | 2018 | [17] |
| BV/FeCoO_x_ | 4.82 mA/cm^2^ | *Adv. Funct. Mater.* | 2018 | [18] |
| BV-VO/Co-Pi | 5.00 mA/cm^2^ | *Adv. Mater.* | 2019 | [19] |
| R-BV/CoPy/FN-H | 4.75 mA/cm^2^ | *Angew. Chem. Int. Ed.* | 2019 | [20] |
| BV/PE | 4.50 mA/cm^2^ | *Angew. Chem. Int. Ed.* | 2020 | [21] |
| BV/F_1_N_3_-H | 3.65 mA/cm^2^ | *Angew. Chem. Int. Ed.* | 2020 | [22] |
| BV/Ni-N_4_-O/FeOOH | 6.00 mA/cm^2^ | *J. Am. Chem. Soc.* | 2021 | [23] |
| Ov-BV/NiFe-MOFs | 5.30 mA/cm^2^ | *Angew. Chem. Int. Ed.* | 2021 | [24] |
| BV/Co(OH)_x_-Ag | 3.95 mA/cm^2^ | *Adv. Energy Mater.* | 2021 | [25] |
| BV/A-CoMoO_4-x_ | 3.50 mA/cm^2^ | *ACS Catal.* | 2022 | [26] |
| BV@Fh-MXene | 4.55 mA/cm^2^ | *Appl. Catal. B: Environ. Energy* | 2022 | [27] |
| C-BV/CQDs | 4.83 mA/cm^2^ | *Adv. Funct. Mater.* | 2022 | [28] |
| BV/ε-FeOOH | 5.40 mA/cm^2^ | *ACS Catal.* | 2023 | [29] |
| BV/VO_x_ | 6.29 mA/cm^2^ | *Angew. Chem. Int. Ed.* | 2023 | [30] |
| BV_6%_/NiFeO_x_ | 4.20 mA/cm^2^ | *Angew. Chem. Int. Ed.* | 2023 | [31] |
| BV/Fe_2_TiO_5_ | 3.03 mA/cm^2^ | *J. Am. Chem. Soc.* | 2024 | [32] |
| Tm-BV/Cu-RuO_2_ | 5.3 mA/cm^2^ | *Adv. Funct. Mater.* | 2024 | [33] |
| Ov-BV/MIL-101 | 5.91 mA/cm^2^ | *Adv. Mater.* | 2025 | [34] |
| BV/CoO_x_/MnO_x_/FeNiOOH | **6.75 mA/cm^2^** | **Present work** | | |

**Table S2.** EIS of different samples.

| **Samples** | **BV** | **BV/CoO_x_** | **BV/MnO_x_** | **BV/CoO_x_/MnO_x_** | **BV/FeNiOOH** | **BV/CoO_x_/MnO_x_/FeNiOOH** |
| --- | --- | --- | --- | --- | --- | --- |
| ***R*_s_ (Ω)** | 43.1 | 42.2 | 41.6 | 42.1 | 38.1 | 37.6 |
| ***R*_ct_ (Ω)** | 775 | 618 | 670 | 557 | 327 | 194 |

**Table S3.** The average fluorescence lifetimes of BV/CoO_x_/MnO_x_/FeNiOOH and BV.

| **Samples** | ***τ*_1_ (ns)** | ***τ*_2_ (ns)** | ***τ*_aver_ (ns)** |
| --- | --- | --- | --- |
| **BV** | 1.47 | 10.87 | 2.43 |
| **BV/CoO_x_/MnO_x_/FeNiOOH** | 2.82 | 16.08 | 4.45 |

**Table S4.** Transit time values of different samples.

| **Samples** | **BV** | **BV/CoO_x_/MnO_x_** | **BV/FeNiOOH** | **BV/CoO_x_/MnO_x_/FeNiOOH** |
| --- | --- | --- | --- | --- |
| ***f*_IMPS_ (Hz)** | 215.44 | 261.02 | 316.23 | 464.16 |
| ***τ*_d_ (ms)** | 0.74 | 0.61 | 0.50 | 0.34 |

**Table S5.** EIS of different samples under dark.

| **Samples** | **BV** | **BV/MnO_x_** | **BV/CoO_x_** | **BV/CoO_x_/MnO_x_** |
| --- | --- | --- | --- | --- |
| ***R*_s_ (Ω)** | 41.2 | 40.0 | 48.2 | 45.2 |
| ***R*_ct_ (KΩ)** | 243 | 135 | 105 | 66.7 |

**Table S6.** EIS of different samples under dark.

| **Samples** | **BV** | **BV/CoO_x_/MnO_x_** | **BV/FeNiOOH** | **BV/CoO_x_/MnO_x_/FeNiOOH** |
| --- | --- | --- | --- | --- |
| ***R*_s_ (Ω)** | 41.2 | 45.2 | 48.7 | 42.6 |
| ***R*_ct_ (KΩ)** | 243 | 66.7 | 63.7 | 24.6 |

**Table S7.** EIS of different samples.

| **Samples** | **BV** | **BV/CoO_x_** | **BV/CeO_x_** | **BV/CoO_x_/CeO_x_** | **BV/FeNiOOH** | **BV/CoO_x_/CeO_x_/FeNiOOH** |
| --- | --- | --- | --- | --- | --- | --- |
| ***R*_s_ (Ω)** | 43.1 | 42.2 | 42.0 | 38.9 | 38.1 | 41.5 |
| ***R*_ct_ (Ω)** | 775 | 618 | 682 | 592 | 327 | 234 |

**Table S8.** Transit time values of different samples.

| **Samples** | **BV** | **BV/CoO_x_/CeO_x_** | **BV/FeNiOOH** | **BV/CoO_x_/CeO_x_/FeNiOOH** |
| --- | --- | --- | --- | --- |
| ***f*_IMPS_ (Hz)** | 215.44 | 261.02 | 316.23 | 383.12 |
| ***τ*_d_ (ms)** | 0.74 | 0.61 | 0.50 | 0.42 |

**Table S9.** EIS of different samples under dark.

| **Samples** | **BV** | **BV/CoO_x_** | **BV/CeO_x_** | **BV/CoO_x_/CeO_x_** | **BV/FeNiOOH** | **BV/CoO_x_/CeO_x_/FeNiOOH** |
| --- | --- | --- | --- | --- | --- | --- |
| ***R*_s_ (Ω)** | 41.2 | 48.2 | 47.1 | 40.8 | 48.7 | 41.4 |
| ***R*_ct_ (KΩ)** | 243 | 105 | 152 | 91.1 | 63.7 | 23.5 |

# References

[1] H.-J. Ahn, K.-Y. Yoon, M. Sung, H. Yoo, H. Ahn, B. H. Lee, J. Lee, J.-H. Jang, *ACS Energy Lett.* **2023**, *8*, 2595-2602.

[2] K. Song, H. Hou, D. Zhang, F. He, W. Yang, *Appl. Catal. B Environ. Energy* **2023**, *330*, 122630.

[3] Y. Sun, H. Li, Y. Hu, J. Wang, A. Li, P. F.-X. Corvini, *Appl. Catal. B Environ. Energy* **2024**, *340*, 123269.

[4] aG. Kresse, J. Furthmüller, *Comp. Mater. Sci.* **1996**, *6*, 15-50; bG. Kresse, J. Furthmüller, *Phys. Rev. B* **1996**, *54*, 11169-11186.

[5] M. Ernzerhof, J. P. Perdew, *J. Chem. Phys.* **1998**, *109*, 3313-3320.

[6] S. Grimme, J. Antony, S. Ehrlich, H. Krieg, *J. Chem. Phys.* **2010**, *132*, 154104.

[7] J. Rossmeisl, A. Logadottir, J. K. Nørskov, *Chemical Physics* **2005**, *319*, 178-184.

[8] a)S. Oh, S. Jung, Y. H. Lee, J. T. Song, T. H. Kim, D. K. Nandi, S.-H. Kim, J. Oh, *ACS Catal.* **2018**, *8*, 9755-9764; b) F. Hu, Y. Peng, J. Chen, S. Liu, H. Song, J. Li, *Appl. Catal. B Environ. Energy* **2019**, 240, 329-336.

[9] H. Wu, L. Zhang, A. Du, R. Irani, R. van de Krol, F. F. Abdi, Y. H. Ng, *Nat. Commun.* **2022**, *13*, 6231.

[10] S. Gao, K. Geng, *Nano Energy* **2014**, 6, 44-50.

[11] B. Zhang, S. Yu, Y. Dai, X. Huang, L. Chou, G. Lu, G. Dong, Y. Bi, *Nat. Commun.* **2021**, *12*, 6969.

[12] Y. Ma, C. A. Mesa, E. Pastor, A. Kafizas, L. Francàs, F. Le Formal, S. R. Pendlebury, J. R. Durrant, *ACS Energy Lett.* **2016**, *1*, 618-623.

[13] Y. Ma, A. Kafizas, S. R. Pendlebury, F. Le Formal, J. R. Durrant, *Adv. Funct. Mater.* **2016**, *26*, 4951-4960.

[14] S. Wang, P. Chen, J. H. Yun, Y. Hu, L. Wang, *Angew. Chem. Int. Ed.* **2017**, *129*, 8620-8624.

[15] W. Liu, H. Liu, L. Dang, H. Zhang, X. Wu, B. Yang, Z. Li, X. Zhang, L. Lei, S. Jin, *Adv. Funct. Mater.* **2017**, *27*, 1603904.

[16] B. Zhang, L. Wang, Y. Zhang, Y. Ding, Y. Bi, *Angew. Chem. Int. Ed.* **2018**, *57*, 2248-2252.

[17] S. Ye, C. Ding, R. Chen, F. Fan, P. Fu, H. Yin, X. Wang, Z. Wang, P. Du, C. Li, *J. Am. Chem. Soc.* **2018**, *140*, 3250-3256.

[18] S. Wang, T. He, J. H. Yun, Y. Hu, M. Xiao, A. Du, L. Wang, *Adv. Funct. Mater.* **2018**, *28*, 1802685.

[19] H. Ren, T. Dittrich, H. Ma, J. N. Hart, S. Fengler, S. Chen, Y. Li, Y. Wang, F. Cao, M. Schieda, *Adv. Mater.* **2019**, *31*, 1807204.

[20] X. Ning, B. Lu, Z. Zhang, P. Du, H. Ren, D. Shan, J. Chen, Y. Gao, X. Lu, *Angew. Chem. Int. Ed.* **2019**, *58*, 16800-16805.

[21] S. Feng, T. Wang, B. Liu, C. Hu, L. Li, Z. J. Zhao, J. Gong, *Angew. Chem. Int. Ed.* **2020**, *132*, 2060-2064.

[22] X. Ning, P. Du, Z. Han, J. Chen, X. Lu, *Angew. Chem. Int. Ed.* **2021**, *133*, 3546-3551.

[23] X. Zhang, P. Zhai, Y. Zhang, Y. Wu, C. Wang, L. Ran, J. Gao, Z. Li, B. Zhang, Z. Fan, *J. Am. Chem. Soc.* **2021**, *143*, 20657-20669.

[24] J. B. Pan, B. H. Wang, J. B. Wang, H. Z. Ding, W. Zhou, X. Liu, J. R. Zhang, S. Shen, J. K. Guo, L. Chen, *Angew. Chem. Int. Ed.* **2021**, *60*, 1433-1440.

[25] X. Ning, D. Yin, Y. Fan, Q. Zhang, P. Du, D. Zhang, J. Chen, X. Lu, *Adv. Energy Mater.* **2021**, *11*, 2100405.

[26] S. Ren, M. Sun, X. Guo, X. Liu, X. Zhang, L. Wang, *ACS Catal.* **2022**, *12*, 1686-1696.

[27] W. Bai, Y. Zhou, G. Peng, J. Wang, A. Li, P. F.-X. Corvini, *Appl. Catal. B Environ. Energy* **2022**, *315*, 121606.

[28] Y. Wang, D. Chen, J. Zhang, M. S. Balogun, P. Wang, Y. Tong, Y. Huang, *Adv. Funct. Mater.* **2022**, *32*, 2112738.

[29] Y.-F. Hu, Y.-F. Li, Z.-P. Liu, *ACS Catal.* **2023**, *13*, 10167-10176.

[30] B. Liu, X. Wang, Y. Zhang, L. Xu, T. Wang, X. Xiao, S. Wang, L. Wang, W. Huang, *Angew. Chem. Int. Ed.* **2023**, *62*, e202217346.

[31] N. Yang, S. Zhang, Y. Xiao, Y. Qi, Y. Bao, P. Xu, S. Jin, F. Zhang, *Angew. Chem. Int. Ed.* **2023**, *62*, e202308729.

[32] Q. Wang, L. I. Oldham, A. Giner-Requena, Z. Wang, D. Benetti, S. Montilla-Verdú, R. Chen, D. Du, T. Lana-Villarreal, U. Aschauer, N. Guijarro, J. R. Durrant, J. Luo, *J. Am. Chem. Soc.* **2024**, *146*, 34681-34689

[33] K. Tian, L. Jin, A. Mahmood, H. Yang, P. An, J. Zhang, Y. Ji, Y. Li, D. Li, S. Liu, J. Yan, *Adv. Funct. Mater.* **2024**, *34*, 2410548.

[34] Y. Xin, J. Tian, X. Xiong, C. Wu, S A. Carabineiro, X. Yang, Z. Chen, Y. Xia, Y. Jin, *Adv. Mater.* **2025**, 2417589.
